# Lipid Profiles of the Heads of Four Shrimp Species by UPLC–Q–Exactive Orbitrap/MS and Their Cardiovascular Activities

**DOI:** 10.3390/molecules27020350

**Published:** 2022-01-06

**Authors:** Yongqiang Zhu, Peihai Li, Ronghua Meng, Xiaobin Li, Yuezi Qiu, Lizheng Wang, Shanshan Zhang, Xuanming Zhang, Houwen Lin, Hongbin Zhai, Kechun Liu

**Affiliations:** 1 Engineering Research Center of Zebrafish Models for Human Diseases and Drug Screening of Shandong Province, Key Laboratory for Biosensor of Shandong Province, Biology Institute, Qilu University of Technology, Shandong Academy of Sciences, Jinan 250103, China; m17864181498@163.com (Y.Z.); lipeihaihg@sina.com (P.L.); qyz970516@163.com (Y.Q.); wlzh1106@126.com (L.W.); qingshuibaikai@126.com (S.Z.); lenghanxing@163.com (X.Z.); 2 Bioengineering Technology Innovation Center of Shandong Province, Qilu University of Technology, Shandong Academy of Sciences, Heze 274000, China; 3 Physical and Chemical Examination Division, Zoucheng Center for Disease Control and Prevention, Zoucheng 273500, China; mrh777@163.com; 4 Research Center for Marine Drugs, State Key Laboratory of Oncogenes and Related Genes, Department of Pharmacy, School of Medicine, Shanghai Jiao Tong University, Shanghai 200127, China; franklin67@126.com; 5 Key Laboratory of Chemical Genomics, School of Chemical Biology and Biotechnology, Shenzhen Graduate School of Peking University, Shenzhen 518055, China; zhaihb@pku.edu.cn

**Keywords:** lipidomics, zebrafish, pro-angiogenesis, anti-inflammatory, antithrombosis, cardioprotective

## Abstract

Lipids are key factors in nutrition, structural function, metabolic features, and other biological functions. In this study, the lipids from the heads of four species of shrimp (*Fenneropenaeus chinensis* (FC), *Penaeus japonicus* (PJ), *Penaeus vannamei* (PV), and *Procambarus clarkia* (PCC)) were compared and characterized based on UPLC–Q–Exactive Orbitrap/MS. We compared the differences in lipid composition of four kinds of shrimp head using multivariate analysis. In addition, a zebrafish model was used to evaluate pro-angiogenic, anti-inflammatory, anti-thrombotic, and cardioprotective activities of the shrimp head lipids. The lipids from the four kinds of shrimp head had different degrees of pro-angiogenic activities, and the activities of PCC and PJ shrimp lipids were more significant than those of the other two species. Four lipid groups displayed strong anti-inflammatory activities. For antithrombotic activity, only PCC (25 μg/mL) and PV (100 μg/mL) groups showed obvious activity. In terms of cardioprotective activity, the four kinds of lipid groups significantly increased the zebrafish heart rhythms. The heart distances were shortened, except for those of the FC (100 μg/mL) and PJ (25 μg/mL) groups. Our comprehensive lipidomics analysis and bioactivity study of lipids from different sources could provide a basis for the better utilization of shrimp.

## 1. Introduction

Lipid is a general term used for fats and lipoids, compounds that are insoluble in water but soluble in nonpolar solvents (e.g., alcohol, ether, chloroform, or benzene) Fats and lipoids are important biological organic compounds since they are key factors in nutrition, structural function, metabolic function, and other biological functions [1]. From the perspective of chemical structure, lipids can be divided into eight categories: fatty acyls (FA), glycerolipids (GL), glycerophospholipids (GP), sphingolipids (SP), sterol lipids (ST), prenolipids (PR), saccharolipids (SL), and polyketides (PK) [2]. Various lipid structures endow them with a variety of important biological functions, including energy conversion, material transport, cell development and differentiation, identification and delivery information, and apoptosis [3,4,5,6]. In addition, lipids also have a variety of biological activities, such as cardiovascular protective activity [7,8], pro-angiogenic activity [9], hepatic protective activity [10], and anti-inflammatory activity [11,12]. Therefore, the identification of lipids using a high-throughput and sensitive mass spectrometry method is crucial for elucidating the role of lipids in cell function and disease treatment.

Following the development of transcriptomics and proteomics, the new research field of lipidomics was formally proposed in 2003 [13]. Lipidomics is an independent discipline for the systematic study of lipids. As a methodology for large-scale qualitative and quantitative study of lipids and for understanding their functions and changes under different physiological and pathological conditions, lipidomics can accurately and comprehensively provide spectra of biological samples under different physiological conditions [14]. Moreover, lipidomics comprises the identification and qualitative and quantitative analysis of lipids in a given biological system, including how lipid molecules affect lipid metabolism and the function of the entire system [15]. The process of lipidomics includes the extraction, separation, analysis, and identification of lipids and the corresponding bioinformatics techniques. The current identification techniques of lipidomics include the shotgun method [16], liquid chromatography–mass spectrometry (LC–MS) [17,18,19], matrix-assisted laser desorption mass spectrometry (MALDI–TOF–MS) [20], nuclear magnetic resonance (NMR) [21], thin layer chromatography (TLC) [22], and gas chromatography–mass spectrometry (GC–MS) [23]. Rapid and high-precision analysis and detection of various trace lipids in biological samples have been realized with the development of soft ionization technology and high-resolution mass spectrometry technology [24].

Shrimp have high nutritional and economic value, accounting for a large proportion of the aquaculture industry [25]. Shrimp heads, comprising more than one third of the shrimp body weight, are often used as processing waste or in feed production, which not only pollutes the environment but also wastes resources [26,27]. Early studies indicated that shrimp heads were rich in lipid resources [28,29]. Consequently, the utilization of shrimp heads as a source of lipids can increase economic value, decrease resource waste, and reduce environmental pressure.

Herein, the lipids extracted from the heads of four species of shrimp (*Fenneropenaeus chinensis* (FC), *Penaeus japonicus* (PJ), *Penaeus vannamei* (PV), and *Procambarus clarkia* (PCC), were systematically studied and comprehensively compared based on the UPLC–Q–Exactive Orbitrap/MS method. These four species of shrimp are the most common representative shrimp in Shandong Province, China. Currently, there are few reports concerning the lipidomics of shrimp heads. In this study, we conducted quantitative and qualitative analysis of these four species of shrimp head lipids and compared the differences in lipid molecules. The clarification of differences will be beneficial for researchers and others to obtain various lipids more efficiently and to develop and utilize different shrimp head resources in a targeted manner. In addition, zebrafish models were used to evaluate the activities of the lipids contained in shrimp head extracts toward pro-angiogenesis, anti-inflammation, anti-thrombus, and cardioprotective effects. The zebrafish is an important model vertebrate in current biological research [30]. Compared with other animal models, zebrafish have many advantages, such as higher homology with human genes, good permeability to small molecules, and a cardiovascular system similar to that of mammals in anatomical structure and physiological function [31,32]. Our goal is to analyze and compare the composition, distribution, and activities of four representative local shrimp head lipids, consequently providing a basis for development and utilization of local shrimp resources.

## 2. Results

### 2.1. Lipid Fingerprint Identification

**Figure 1:**
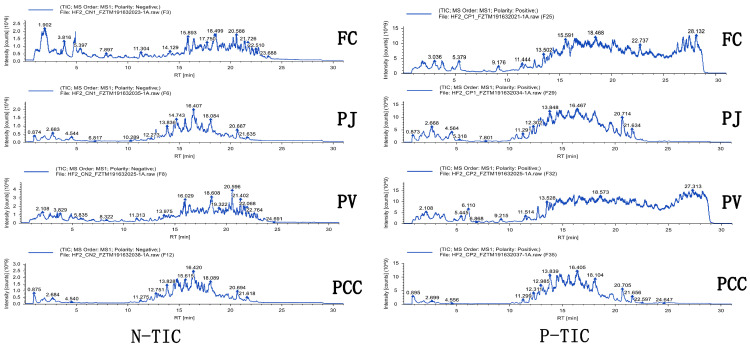
Total ion chromatograms (TICs) of lipid extracts from *Fenneropenaeus chinensis* (FC), *Penaeus japonicus* (PJ), *Penaeus vannamei* (PV), and *Procambarus clarkia* (PCC). Left: negative ion mode, right: positive ion mode.

**Figure 2:**
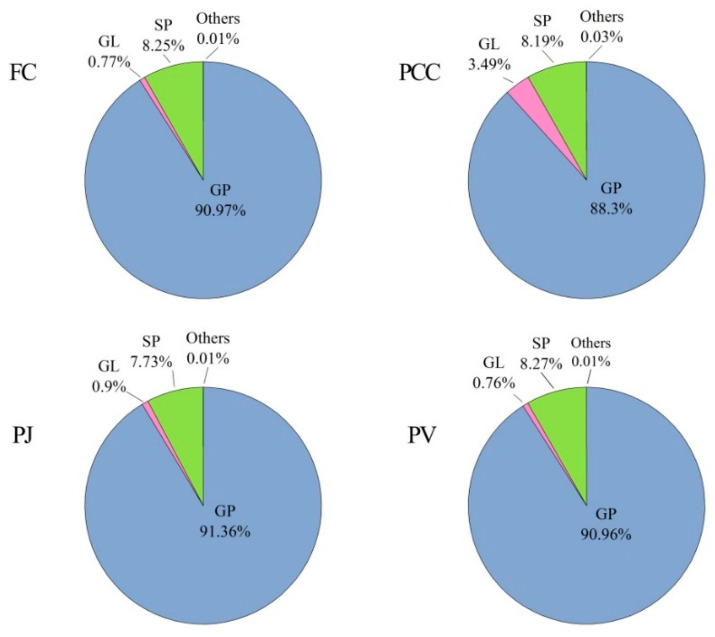
Lipid profiles of four shrimp head species (n = 3) (percentage of total lipids). “Others” including SL and FA.

Four lipid extracts from different sources were analyzed by UPLC–Q–Exactive Orbitrap/MS in positive and negative ion modes. A total of 997 lipids were detected (Table S1 and Figure 1). The extracts comprised five types of lipids, with 761 GP, 2 SL, 23 FA, 193 SP, and 18 GL. As shown in Table S1, the number of carbon atoms in the fatty acids from the heads of the four species of shrimp was between 4 and 38. The fatty acyl chains of GPs, GLs, SLs, and SPs contained 1–26 carbons and 0–6 double bonds per chain. Table S1 showed total molecular ions, exact mass, molecular weight, molecular formulas, retention times (RT), hit scores, mass tolerance, and normalized peak area percentages of five lipid classes in four samples. The relative quantification of lipid molecules was calculated by comparison with the total peak area of lipid molecules. Figure 2 shows the lipid distributions in FC, PCC, PJ, and PV, where FC, PJ, and PV are marine shrimp and PCC is a land shrimp. Clearly, the shrimp heads were rich in GPs, accounting for 90.97% in FC, 88.30% in PCC, 91.36% in PJ, and 90.96% in PV. GP was significantly less in PCC than that in the other three kinds of shrimp heads, suggesting that marine shrimp are richer in phospholipids than land shrimp. Moreover, the GL content of 3.49% in PCC was significantly higher than that of the other three shrimp head lipids. There was no significant difference in the contents of SP in the four shrimp heads, accounting for 8.25% in FC, 8.19% in PCC, 7.73% in PJ, and 8.27% in PV, respectiAAAvely. For other lipid types, including SL and FA, all the contents in the four kinds of shrimp heads were very low (less than 0.1%).

**Figure 3:**
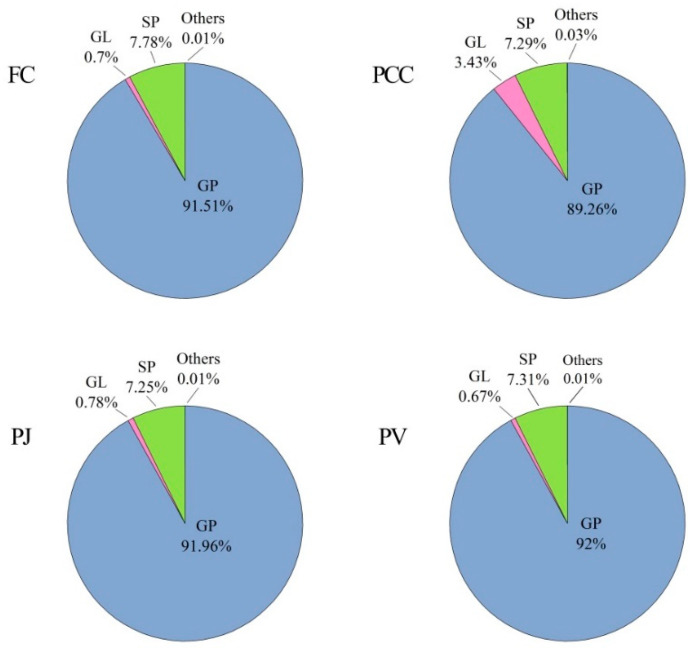
Unsaturated lipid profiles of four shrimp species (n = 3) (percentages of total unsaturated lipids). Others including SL and FA.

The distribution of unsaturated lipids in the heads of FC, PCC, PJ, and PV are shown in Figure 3. The unsaturated GP was the highest proportion of total unsaturated fat in the four types of shrimp heads, accounting for 91.51% in FC, 89.26% in PCC, 91.96% in PJ, and 92% in PV. The proportion of unsaturated GL in PCC, 3.43%, was more than that in FC, PJ, and PV, which accounted for 0.70%, 0.78%, and 0.67%, respectively. The content of unsaturated SP in FC, PCC, PJ, and PV showed little difference, with 7.78% in FC, 7.29% in PCC, 7.25% in PJ, and 7.31% in PV, respectively. Moreover, the contents of unsaturated SL and FA were the lowest (less than 0.1%). The above results can provide the basis for the development and utilization of different kinds of lipids.

### 2.2. Multivariate Analysis

**Figure 4:**
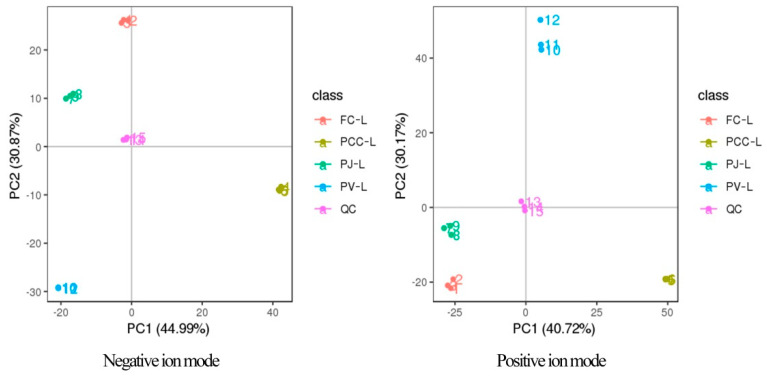
PCA score plots for groups of FC, PJ, PV, and PCC (negative ion mode in the left frame and positive ion mode in the right frame).

MetaX software was applied for multivariate statistical analysis of lipids from four sources [33]. First, PCA was performed on the total sample to determine the lipid differences. As shown in Figure 4, four groups of samples were distributed in different regions, indicating the significant differences in lipids of four different shrimp heads. Furthermore, the lipid compositions of FC and PJ were more similar, being located in the same quadrant.

Secondly, PLS–DA was employed for variance analysis and lipid screening to highlight the differences in lipids. There was a significant separation in the PLS–DA scatter point diagram (Figure S1A,C). The model evaluation parameters (R2Y, Q2Y) were obtained from the PLS–DA sequencing verification diagram (Figure S1B,D). R2Y was greater than Q2Y, indicating that the model was stable and reliable. The lipid screening used VIP > 1.0 from the PLS–DA model and a *p*-value < 0.05 from the *t*-test to identify the differences in lipids among the groups. As a result, 390 lipids showed significant differences between FC and PCC groups, while 203 lipids between FC and PJ, 325 between FC and PV, 400 between PJ and PCC, 337 between PJ and PV, and 328 between PV and PCC were also screened under the same standard. Table 1 lists the top five most discriminating lipids, representing the most significant differences between pairs of groups.

**Figure 5:**
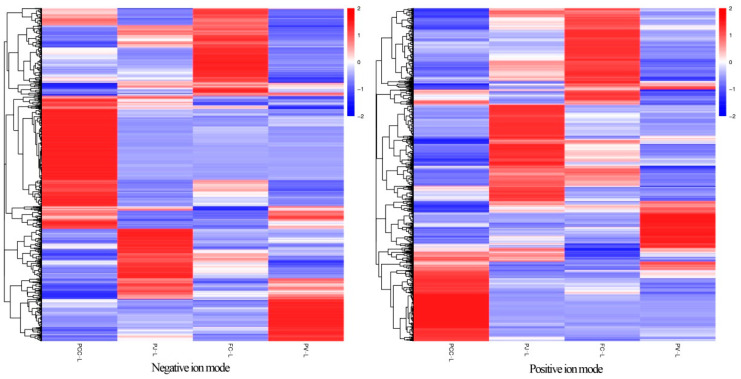
Clustering heat map of total differential metabolites (negative ion mode on the left and positive ion mode on the right).

Figure 5 shows a heat map of total differential metabolites. The longitudinal axis shows the clustering of samples, while the transverse is the clustering of metabolites. The shorter the clustering branches, the higher the similarity. The clustering of metabolite content between groups can be seen by the horizontal comparison. Figure 5 and Figure S2 indicate that the lipid compositions of each shrimp head sample were clearly different.

### 2.3. Pro-Angiogenic Activity

**Figure 6:**
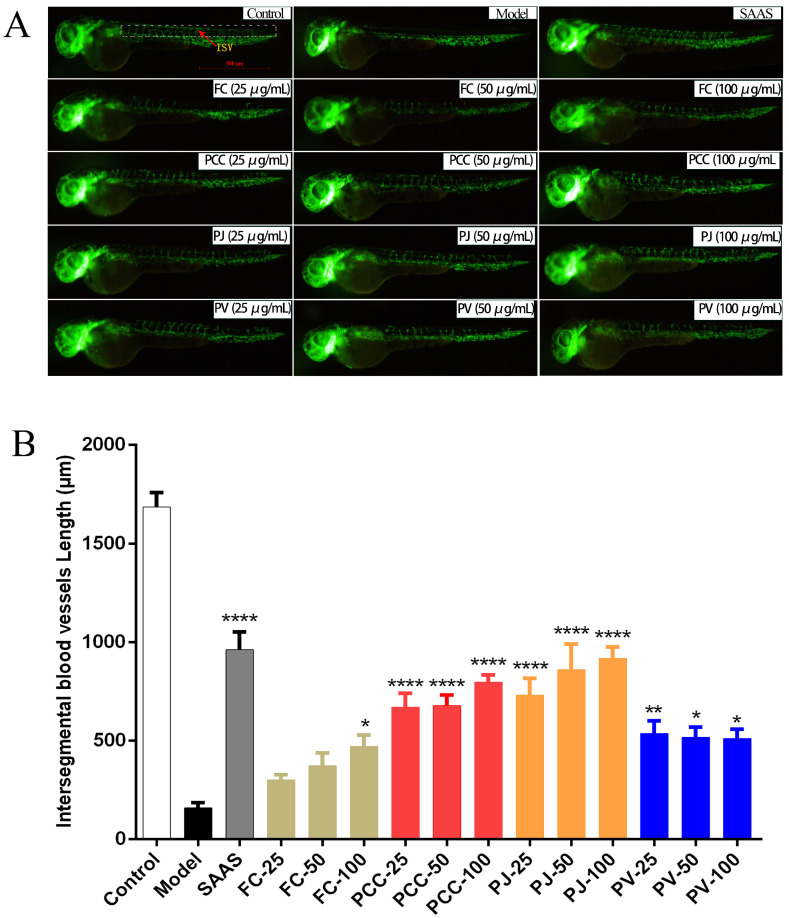
Angiogenic activities of lipid extracts. (**A**) In vivo observation of the intersegmental blood vessel length of zebrafish; (**B**) Statistical analysis results for zebrafish intersegmental blood vessels (ISV) for all treated groups. Comparisons with model group. * *p* < 0.05, ** *p* < 0.01, **** *p* < 0.0001.

In this study, the model of zebrafish by PTK787 was used to study the angiogenesis-promoting activity of lipids from the heads of four species of shrimp [34]. All lipid groups had different degrees of angiogenesis-promoting activities. In PCC, PJ, and PV groups, the lipids showed significant activity at low, medium, and high concentrations. PJ had the strongest activity, expressed in a dose-dependent manner. The activity of FC lipids was relatively weak and showed a certain pro-angiogenic activity only at the high concentration (100 μg/mL) (Figure 6).

### 2.4. Anti-Inflammatory Activity

**Figure 7:**
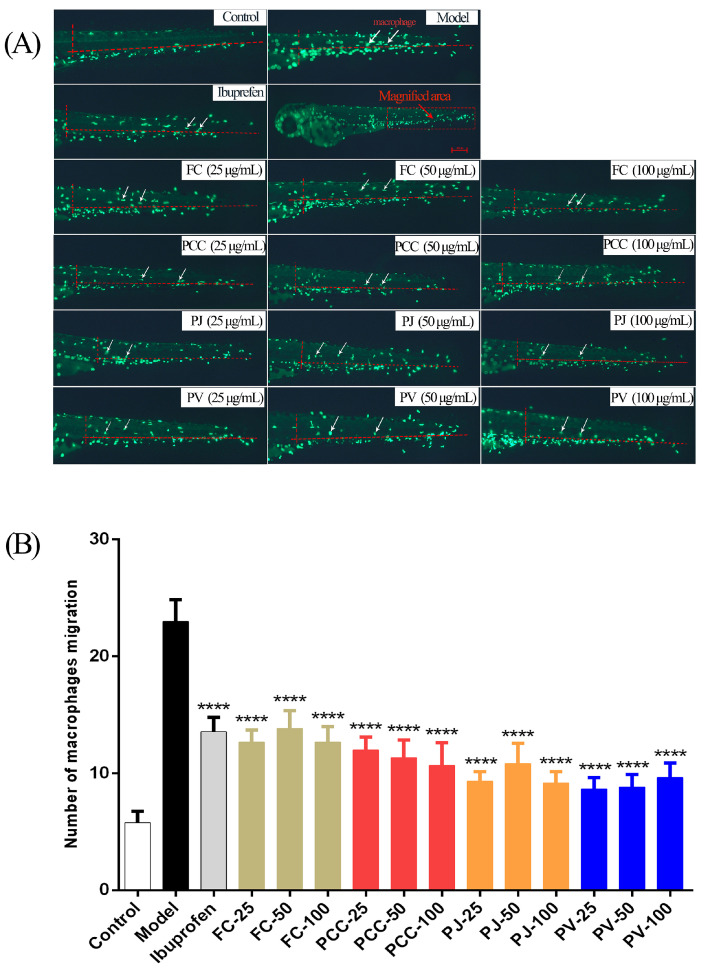
Anti-inflammatory activities of lipid extracts (n = 8, $\bar{x}$ ± s). (**A**) In vivo observation of macrophage migration in zebrafish; (**B**) Statistical analysis results of macrophage migration numbers in each group in comparison with the model group. **** *p* < 0.0001.

CuSO_4_ can induce a strong inflammatory response in zebrafish lateral line nerve mast cells and mechanosensory cells, thereby stimulating the infiltration of macrophages [35]. Compared with the CuSO_4_ group, all shrimp head lipids significantly reduced the migration of macrophages (Figure 7), indicating their anti-inflammatory activity (*p* < 0.01). Except for the 50 μg/mL FC group, the anti-inflammatory activities of the other groups were superior to that of the positive group. The activity of the PV group was the strongest.

### 2.5. Antithrombotic Activity

**Figure 8:**
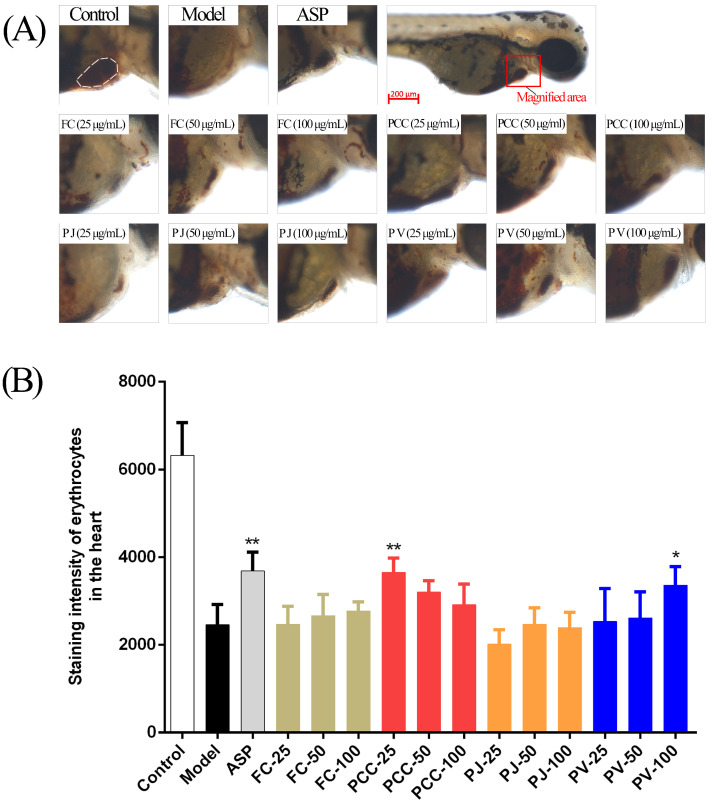
Antithrombotic activities of lipid extracts (n = 10, $\bar{x}$ ± s). (**A**) Observation of erythrocytes in the zebrafish heart in vivo in each group; (**B**) Statistics of the staining area of red blood cells in each group in comparison with the model group. * *p* < 0.05, ** *p* < 0.01.

As illustrated in Figure 8, the staining intensity (SI) of zebrafish heart erythrocytes in the model group (80 μM AA) was significantly decreased, indicating an increase in caudal vein thrombosis [36]. Both PCC (25 μg/mL) and PV (100 μg/mL) groups showed significant antithrombotic activity (*p* < 0.05). The other groups had no antithrombotic activity.

### 2.6. Cardiac Protective Activity

**Figure 9:**
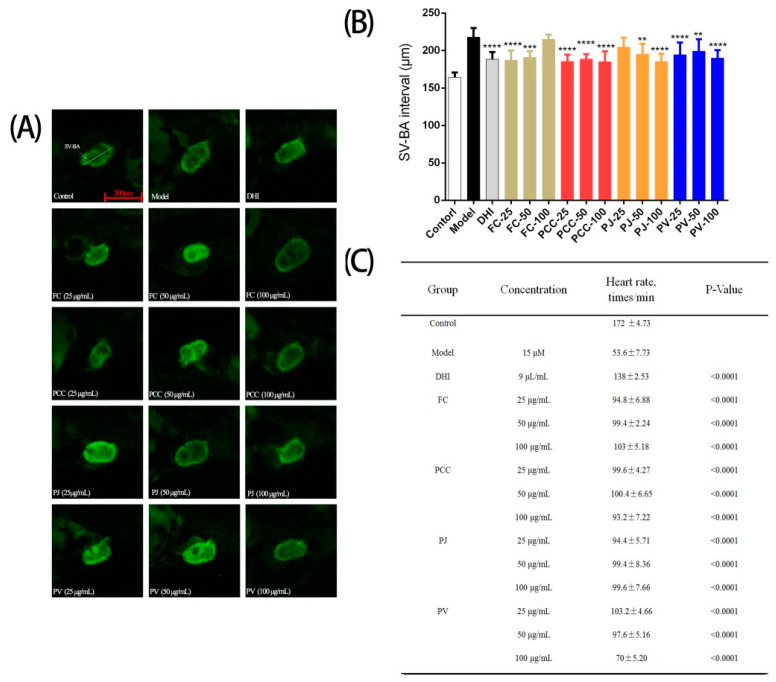
Cardiac protective activities of lipid extracts (n = 10, $\bar{x}$ ± s) (**A**) In vivo observation of zebrafish hearts in each group; (**B**) Heart rate measurement results of zebrafish in each group; (**C**) SV-BA statistics of heart in each group in comparison with the model group. ** *p* < 0.01, *** *p* < 0.001, **** *p* < 0.0001.

We studied the cardiac protective activities of shrimp head lipids via the zebrafish heart injury model (using 15 μM terfenadine) and evaluated the effect by measuring the heart rate and sinus venosus and bulb arterial (SV-BA) distance of zebrafish [37]. The results (Figure 9) showed that heart injury symptoms appeared in the model group, while the positive control and lipids from the four species significantly reduced the above symptoms. In detail, compared with the blank control group, the heart rate of the zebrafish in the model group was significantly reduced, and the SV–BA distance in the heart was remarkably increased (*p* < 0.01), indicating that terfenadine caused heart injury in the zebrafish. Compared with the model group, the heart rates of zebrafish in the positive control group (Danhong injection (DHI)) and the lipid groups were increased, and the heart SV–BA interval was shortened. In general, both indexes indicated that shrimp head lipids had significant cardioprotective activity, but different indicators showed different intensities of activity. For example, the heart rate of the FC (100 μg/mL) group was notably increased compared to the model group, while the shortening range of SV–BA distance was not significant.

## 3. Discussion

Lipids are considered to be important nutrients for human beings, and they are widely used in food, medical treatment, health care, and basic research. It has been reported that shrimp heads are rich in lipid components [38]. At present, the study on the lipids of shrimp heads has attracted increasing attention. In our previous study, we used lipidomics to compare the differences between lipids from shrimp heads and other sources [39]. We isolated phosphatidylcholine (PC), phosphatidylethanolamine (PE), phosphatidylserine (PS), phosphatidylinositol (PI), sphingomyelin (SM), and other types of phospholipids from Penaeus vannamei and comprehensively evaluated their cardiovascular activities. There were significant differences in the composition, distribution, and bioactivity of lipids from different sources. PC showed clear pro-angiogenesis activity [25]. In addition, the structural characteristics of lipids in aquatic products are rich in polyunsaturated fatty acids or polyunsaturated fatty acid side chains [40], which was also confirmed in this paper. Here, the lipid composition and bioactivities of shrimp heads of different species were compared, thereby providing a basis for the further rational utilization of shrimp head resources.

We carried out a composition analysis and cardiovascular activity evaluation for the lipids from four kinds of representative shrimp heads via the UPLC–Q–Exactive Orbitrap/MS approach and zebrafish models. This is the first truly comprehensive comparison and characterization of these lipid sources. A total of 997 lipid structures were identified, with 761 GP, 23 FA, 2 SL, 193 SP, and 18 GL. The most important lipids rich in shrimp heads were GP. The unsaturated lipids in shrimp heads were mainly distributed in GP, GL, and SP types. Multivariate statistical analysis showed that there were significant differences in lipid composition among the four species of shrimp heads and that FC and PJ had relatively similar lipid distributions. Meanwhile, the in vivo cardiovascular activities of lipid samples were evaluated by using the zebrafish models. The lipids of each group showed pro-angiogenesis, anti-inflammatory, and cardioprotective activities, and the pro-angiogenic activities of PCC and PJ lipids were stronger than those of the other two species. For antithrombotic activity, only FC (25 μg/mL) and PV (100 μg/mL) groups showed certain activities. In fact, there is no related literature on whether consuming shrimp reduces cardiovascular risk in humans. According to our experimental results, lipids in shrimp heads can reduce the risk of cardiovascular disease in humans.

In summary, our findings can provide a lipid atlas for aquatic products with a reliable and detailed research basis and a foundation for the targeted development of lipids from different sources. In the future, it will be necessary to separate more lipids from the shrimp head resources and identify their active components.

## 4. Materials and Methods

### 4.1. Chemicals

The chemicals and solvents used for sample extraction and preparation in the experiment were of high purity and analytical grade. HPLC grade solvents, including acetonitrile (CAN), pure water, isopropyl alcohol (IPA), methanol, dichloromethane, formic acid, ammonium acetate, and methyl tert-butyl ether (MTBE), were supplied by Thermo. PTK787 (vascular endothelial cell growth factor receptor inhibitor, purchased from Abcam), salvianolic acid A sodium, aspirin, and ibuprofen were obtained from Bioleaf Biotech Co., Ltd. (Shanghai, China). Terfenadine, Danhong injection (DHI), and arachidonic acid (AA) were purchased from Solarbio (Beijing, China).

### 4.2. Experimental Materials

The shrimp species used in this experiment (FC, PJ, PV, and PCC) are the four most common types of shrimp in Shandong Province, China, and were all purchased from the local seafood markets. The heads were randomly removed from each of the four species.

### 4.3. Lipid Extraction

The four kinds of shrimp heads required for the experiment were stored in the laboratory of the Institute of Biology, Qilu University of Technology (Shandong Academy of Sciences) at −20 °C. Shrimp heads were homogenized separately, and then total lipids were extracted from each sample according to the method of Bligh and Dyer [41].

### 4.4. Sample Processing before Machine Analysis

100 mg of each lipid sample was added to a glass centrifuge tube with a Teflon-lined cap; 0.75 mL of pre-cooled methanol was added, and the tube was vortexed. Then, 2.5 mL of MTBE was added, and the mixture was incubated for 1 h at room temperature in a shaker. Phase separation was induced by adding 0.625 mL of MS-grade water. After standing for 10 min, the sample was centrifuged at 1500 rpm for 10 min. The upper (organic) phase was collected, and the lower phase was reextracted with 1 mL of the solvent mixture (MTBE/methanol/water, 10:3:2.5, *v*/*v*/*v*). Combined organic phases were dried with a sample concentrator (Termovap). To speed up sample drying, 100 μL of MS-grade methanol was added to the organic phase after 25 min of centrifugation. The extracted lipids were stored in 100 μL isopropanol/methanol/water (60:30:4.5, *v*/*v*/*v*) and then analyzed by an LC–MS/MS system. An equal amount of supernatant from each processed sample was taken and mixed as the QC sample.

### 4.5. UPLC–Q–Exactive Orbitrap/MS Analysis

The analysis of lipids was performed using a Thermo Vanquish^TM^ UPLC form Thermo (Waltham, MA, USA). Samples were injected into a Thermo Accucore C30 column using a 31 min linear gradient at a flow rate of 0.35 mL/min. The column temperature was set at 40 °C. Mobile phase buffer A was acetonitrile/water (6/4) with 10 mM ammonium acetate and 0.1% formic acid, whereas buffer B was acetonitrile/isopropanol (1/9) with 10 mM ammonium acetate and 0.1% formic acid. The solvent gradient was set as follows: 30% B, initial; 43% B, 8 min; 50% B, 8.1 min; 70% B, 17 min; 99% B, 24 min; 30% B, 27.1 min; 30% B, 31 min. After each analysis, the column was rebalanced with 30% B for 5 min, and then the next analysis was performed. The Q Exactive mass series spectrometer was operated in positive and negative polarity modes with sheath gas: 20 arbitrary units, sweep gas: 1 arbitrary unit, auxiliary gas rate: 5 (Anion: 7), spray voltage: 3 kV, capillary temperature: 350 °C, heater temperature: 400 °C, S-Lens RF level: 50, resolving power (full scan): 120,000, scan range: 114–1700 *m*/*z*, automatic gain control target: 1 × 10^6^, resolving power (MS^2^): 30,000 (Top20), normalized collision energy: 25; 30 (negative ions 20; 24; 28), injection time: 100 ms, isolation window: 1 *m*/*z*, automatic gain control target (MS^2^): 1 × 10^5^, dynamic exclusion: 15 s.

### 4.6. Experimental Animals

Transgenic zebrafish Tg (FLI1-EGFP), Tg (LYZ: EGFPJS7), AB, and Tg (CMLC2: GFP) strains were used to study the activities of lipids toward pro-angiogenesis, anti-inflammation, anti-thrombus, and cardiac protection, respectively. Zebrafishes from the Key Laboratory of Drug Screening Technology of Shandong Academy of Sciences (Jinan, China) were incubated under a 14 h light/10 h dark cycle at 28 ± 0.5 °C. Embryos were obtained from natural oviposition, and the fertilized eggs were collected, washed three times with fish culture water, and cultured in a light incubator at 28.5 °C. The fertilized embryos were selected for the activity evaluation experiment. The activity assays were conducted according to the standard ethical guidelines. The procedures were approved by the Ethics Committee of the Biology Institute of Shandong Academy of Science.

### 4.7. Activity Assays of Zebrafish

Pro-angiogenic activity assay: 24 hpf (hours post fertilization) Tg (FLI1–EGFP) zebrafish embryos had their egg membranes removed with pronase E and were randomly placed in a 24-well cell culture plate with 10 larvae in each well (two replicate wells for each sample group). The exposure components were divided into seven groups: the vehicle control group (DMSO, 0.1%, *v*/*v*), the model group (PTK787, 0.25 μg/mL), the positive control group (0.25 μg/mL PTK787 + 100 μg/mL SAAS), the FC group (0.25 μg/mL PTK787 + 25, 50, 100 μg/mL FC), the PCC group (0.25 μg/mL PTK787 + 25, 50, 100 μg/mL PCC), the PJ group (0.25 μg/mL PTK787 + 25, 50, 100 μg/mL PJ), and the PV group (0.25 μg/mL PTK787 + 25, 50, 100 μg/mL PV). The samples were mixed, covered, and placed in a constant temperature and light incubator at 28 °C. After 24 h of administration, the intersegmental blood vessels (ISVs) were observed and photographed under a fluorescence microscope. An Image-Pro Plus 5.1 was used to measure the total length of ISVs of zebrafish.

Anti-inflammatory activity assay: 72 hpf zebrafish embryos Tg (LYZ: EGFPJS7) were transferred to a 24-well culture plate, with two duplicate wells in each group (10 larvae per well). Lipid samples from four kinds of shrimp heads (FC, PJ, PV, and PCC) with concentrations of 25, 50, and 100 μg/mL were added to the different drug groups, and ibuprofen (20 μM) was added with culture water to 2.0 mL as the positive control group. All groups were placed in the light incubator (28 °C) for the embryos to continue to develop. After 6 h, 20 μM of CuSO_4_ was used to treat the zebrafish in each group for 1 h except for the vehicle control group. Then, the inflammatory response was observed under a fluorescence microscope to calculate the number of inflammatory cells migrating to the zebrafish tail notochord, and the statistical analysis was carried out with Image-Pro Plus 5.1.

Antithrombotic activity assay: 72 hpf zebrafish (AB) were placed in a 24-well cell culture plate with 10 larvae per well. Except for the different model drug (AA 80 μM) and the positive control (aspirin 22.5 μg/mL), the same design was used to divide the exposure groups as used in the anti-inflammatory activity assay. All the groups were incubated at 28 °C for 1.5 h and then stained in the dark with 1 mg/mL O-dianisidine dye liquor for 10 min. After washing three times with fish culture water, the zebrafish larvae in each group were placed under a microscope to observe venous thrombosis, and the erythrocyte staining areas of the hearts were quantitatively analyzed with the Image-Pro Plus 5.1.

Cardiac protective activity assay: 48 hpf cardiac fluorescent zebrafish embryos Tg (CLMC2: GFP) were placed in a solution of pronase E at 1 mg/mL for about 1 min to remove their outer egg membranes. The exposed group was divided into a vehicle control group, a model group (15 μM terfenadine), a positive control group (15 μM terfenadine + 9 μL/mL danhong injection), and drug groups (15 μM terfenadine + 25, 50, 100 μg/mL lipids from shrimp heads). After incubation at 28 °C for 24 h, the heart rate (n = 6) was counted under a fluorescence microscope. After anesthesia with triamine (0.16%, *w*/*v*), Image Pro-Plus software was used to calculate the distance between the sinus venous and bulb arterial (SV–BA) of each zebrafish.

### 4.8. Data Analysis

The raw data files generated by UPLC–MS/MS were processed using Compound Discoverer 3.0 (Thermo Fisher) to perform peak alignment, peak picking, and quantitation for each metabolite. The main parameters were set as follows: retention time tolerance, 0.2 min; actual mass tolerance, 5 ppm; signal intensity tolerance, 30%; signal/noise ratio, 3; and minimum intensity, 100,000. After that, peak intensities were normalized to the total spectral intensity. The normalized data were used to predict the molecular formulae based on additive ions, molecular ion peaks, and fragment ions. The peaks were matched with the Lipidmaps and Lipidblast databases to obtain accurate qualitative and relative quantitative results. Then, the compound with a CV (Coefficient of Variation) value less than 30% in the QC sample was selected as the final identification result for subsequent analysis [42]. Statistical analyses were performed using the statistical software R (R version R-3.4.3), Python (Python 2.7.6 version), and CentOS (CentOS release 6.6). When data were not normally distributed, normal transformations were attempted using the area normalization method. Principal component analysis (PCA) and partial least squares discriminant analysis (PLS–DA) were performed with metaX. We used univariate analysis (t-tests) to calculate statistical significance. Lipids with VIP > 1 and *p*-value < 0.05 were considered as differential lipids. For the clustering heatmaps, the data were normalized using the Z-score of the differential metabolite strength region, and the heatmaps were drawn using the R-language Pheatmap package.

All activity tests were repeated in triplicate and results are presented as means ± standard deviation. Image-Pro Plus 5.1 was used for statistical analyses. One-way ANOVA was used to analyze differences among groups, and *p* < 0.05 was considered statistically significant.

## Figures and Tables

**Table 1:** Top five lipids showing significant differences between every two groups.

No.	Phospholipid Species	VIP Score	*p*-Value
FC vs. PCC			
1	PC (18:2_18:3)	3.154	1.02 × 10^−3^
2	PC (19:0_16:1)	3.037	2.01 × 10^−2^
3	PC (24:1_22:6)	3.021	1.64 × 10^−4^
4	PE (18:1_24:1)	2.790	1.40 × 10^−5^
5	PC (14:0e_20:4)	2.749	1.98 × 10^−4^
FC vs. PJ			
1	TG (12:0_15:1_18:3)	3.217	1.20 × 10^−4^
2	PC (22:6e_4:0)	3.111	3.46 × 10^−4^
3	TG (18:3_18:3_18:3)	2.973	2.47 × 10^−3^
4	PC (22:6e_2:0)	2.942	1.52 × 10^−4^
5	SM (d29:2_12:1)	2.702	6.30 × 10^−3^
FC vs. PV			
1	PC (14:0e_18:2)	3.766	1.39 × 10^−2^
2	PC (19:0_18:1)	3.607	2.91 × 10^−2^
3	PC (16:0e_3:0)	3.566	7.88 × 10^−5^
4	PC (21:2_20:3)	3.456	4.03 × 10^−2^
5	PC (18:2_18:3)	3.422	4.44 × 10^−4^
PJ vs. PCC			
1	TG (18:3_18:3_18:3)	3.287	1.60 × 10^−4^
2	PC (20:5e_16:4)	3.127	5.37 × 10^−6^
3	PC (17:2_16:3)	2.984	1.50 × 10^−7^
4	PC (18:3_18:2)	2.911	7.21 × 10^−3^
5	PC (19:0_16:1)	2.521	2.48 × 10^−2^
PJ vs. PV			
1	PC (14:0e_18:2)	3.536	1.22 × 10^−2^
2	PC (18:0e_14:0)	3.503	1.04 × 10^−6^
3	PC (18:4e_16:4)	3.437	1.99 × 10^−3^
4	PC (19:0_18:1)	3.290	3.58 × 10^−2^
5	PC (14:0e_18:1)	3.174	7.09 × 10^−3^
PV vs. PCC			
1	PC (21:1_21:1)	3.554	1.75 × 10^−6^
2	PC (24:0_18:1)	3.318	3.94 × 10^−4^
3	PC (21:0_22:1)	3.047	1.53 × 10^−4^
4	TG (18:3_18:3_18:3)	3.042	3.89 × 10^−6^
5	PC (18:1_24:1)	2.849	1.25 × 10^−2^

## Data Availability

Not applicable.

## Supplementary Materials

The following supporting information can be downloaded: Table S1. Lipids molecular species of shrimp heads identified by UPLC–Q–Exactive Orbitrap/MS; Figure S1. Distribution point diagram of PLS–DA and sequencing verification diagram; Figure S2. Clustering heat map of different metabolites in each group (negative ion mode on the left and positive ion mode on the right).

## References

[1] Burdge G.C., Calder P.C. (2015). Introduction to fatty acids and lipids. World Rev. Nutr. Diet..

[2] Fahy E., Subramaniam S., Brown H.A., Glass C.K., Merrill A.H., Murphy R.C., Raetz C.R., Russell D.W., Seyama Y., Shaw W. (2005). A comprehensive classification system for lipids. J. Lipid. Res..

[3] Cuvillier O. (2002). Sphingosine in apoptosis signaling. BBA—Mol.Cell Biol. L.

[4] Di Paolo G., De Camilli P. (2006). Phosphoinositides in cell regulation and membrane dynamics. Nature.

[5] Zu Heringdorf D.M., Jakobs K.H. (2007). Lysophospholipid receptors: Signalling, pharmacology and regulation by lysophospholipid metabolism. BBA—Biomembranes.

[6] Morales A., Lee H., Goni F.M., Kolesnick R., Fernandez-Checa J.C. (2007). Sphingolipids and cell death. Apoptosis.

[7] Bunea R., Farrah K.E., Deutsch L. (2005). Evaluation of the effects of neptune krill oil on the clinical course of hyperlipidemia. Altern. Med. Rev..

[8] Hirafuji M., Machida T., Hamaue N., Minami M. (2003). Cardiovascular protective effects of n-3 polyunsaturated fatty acids with special emphasis on docosahexaenoic acid. J. Pharmacol. Sci..

[9] Arshad A., Chung W.Y., Steward W., Metcalfe M.S., Dennison A.R. (2013). Reduction in circulating pro-angiogenic and pro-inflammatory factors is related to improved outcomes in patients with advanced pancreatic cancer treated with gemcitabine and intravenous omega-3 fish oil. HPB.

[10] Hsu D.Z., Chen K.T., Chien S.P., Li Y.H., Huang B.M., Chuang Y.C., Liu M.Y. (2006). Sesame oil attenuates acute iron-induced lipid peroxidation-associated hepatic damage in mice. Shock.

[11] Deutsch L. (2007). Evaluation of the effect of neptune krill oil on chronic inflammation and arthritic symptoms. J. Am. Coll. Nutr..

[12] Li Q., Liang X., Zhao L., Zhang Z., Xue X., Wang K., Wu L. (2017). Uplc-Q-exactive orbitrap/MS-based lipidomics approach to characterize lipid extracts from bee pollen and their in vitro anti-inflammatory properties. J. Agric. Food Chem..

[13] Han X., Gross R.W. (2005). Shotgun lipidomics: Electrospray ionization mass spectrometric analysis and quantitation of cellular lipidomes directly from crude extracts of biological samples. Mass Spectrom. Rev..

[14] Tang H.Y., Wang C.H., Ho H.Y., Wu P.T., Hung C.L., Huang C.Y., Wu P.R., Yeh Y.H., Cheng M.L. (2018). Lipidomics reveals accumulation of the oxidized cholesterol in erythrocytes of heart failure patients. Redox Biol..

[15] Blanksby S.J., Mitchell T.W. (2010). Advances in mass spectrometry for lipidomics. Annu. Rev. Anal. Chem..

[16] Wang J., Han X. (2019). Analytical challenges of shotgun lipidomics at different resolution of measurements. TrAC Trend Anal. Chem..

[17] Dunn W.B., Broadhurst D., Begley P., Zelena E., Francis-McIntyre S., Anderson N., Brown M., Knowles J.D., Halsall A., Haselden J.N. (2011). Procedures for large-scale metabolic profiling of serum and plasma using gas chromatography and liquid chromatography coupled to mass spectrometry. Nat. Protoc..

[18] Li L., Chang M., Tao G., Wang X., Liu Y., Liu R., Jin Q., Wang X. (2016). Analysis of phospholipids in *Schizochytrium* sp. S31 by using UPLC-Q-TOF-MS. Anal. Methods.

[19] Cajka T., Fiehn O. (2014). Comprehensive analysis of lipids in biological systems by liquid chromatography-mass spectrometry. TrAC Trend Anal. Chem..

[20] Fernández J.A., Ochoa B., Fresnedo O., Giralt M.T., Rodríguez-Puertas R. (2011). Matrix-assisted laser desorption ionization imaging mass spectrometry in lipidomics. Anal. Bioanal. Chem..

[21] Li J., Vosegaard T., Guo Z. (2017). Applications of nuclear magnetic resonance in lipid analyses: An emerging powerful tool for lipidomics studies. Prog. Lipid Res..

[22] Li M., Zhou Z., Nie H., Bai Y., Liu H. (2011). Recent advances of chromatography and mass spectrometry in lipidomics. Anal. Bioanal. Chem..

[23] Quehenberger O., Armando A.M., Dennis E.A. (2011). High sensitivity quantitative lipidomics analysis of fatty acids in biological samples by gas chromatography-mass spectrometry. BBA—Mol.Cell Biol. L.

[24] Shen Q., Yang Q., Cheung H.Y. (2015). Hydrophilic interaction chromatography based solid-phase extraction and MALDI TOF mass spectrometry for revealing the influence of Pseudomonas fluorescens on phospholipids in salmon fillet. Anal. Bioanal. Chem..

[25] Zhang M., Li P., Wang F., Zhang S., Li H., Zhang Y., Wang X., Liu K., Li X. (2021). Separation, identification and cardiovascular activities of phospholipid classes from the head of *Penaeus vannamei* by lipidomics and zebrafish models. Food Funct..

[26] Cobos M.A., Garcia L.E., González S., Barcena J.R., Hernández D.S., Sato M.P. (2002). The effect of shrimp shell waste on ruminal bacteria and performance of lambs. Anim. Feed Sci. Technol..

[27] Coward-Kelly G., Agbogbo F.K., Holtzapple M.T. (2006). Lime treatment of shrimp head waste for the generation of highly digestible animal feed. Bioresour. Technol..

[28] Yang X., Zu T.H., Zheng Q.W., Zhang Z.S. (2013). Supercritical carbon dioxide extraction of the fatty acids from pacific white shrimp waste (*Litopenaeus vannamei*). Adv. Mater. Res..

[29] Scurria A., Tixier A.S.F., Lino C., Pagliaro M., D’Agostino F., Avellone G., Chemat F., Ciriminna R. (2020). High yields of shrimp oil rich in omega-3 and natural astaxanthin from shrimp waste. ACS Omega.

[30] Asnani A., Peterson R.T. (2014). The zebrafish as a tool to identify novel therapies for human cardiovascular disease. Dis. Model. Mech..

[31] Xin S.C., Zhao Y.Q., Li S., Lin S., Zhong H.B. (2012). Application of zebrafish models in drug screening. Genetics.

[32] Bournele D., Beis D. (2016). Zebrafish models of cardiovascular disease. Heart Fail. Rev..

[33] Wen B., Mei Z., Zeng C., Liu S. (2017). MetaX: A flexible and comprehensive software for processing metabolomics data. BMC Bioinform..

[34] Tal T.L., Mccollum C.W., Harris P.S., Olin J., Kleinstreuer N., Wood C.E., Hans C., Shah S., Merchant F.A., Bondesson M. (2014). Immediate and long-term consequences of vascular toxicity during zebrafish development. Reprod. Toxicol..

[35] Gui Y.H., Jiao W.H., Zhou M., Zhang Y., Zeng D.Q., Zhu H.R., Liu K.C., Sun F., Chen H.F., Lin H.W. (2019). Septosones A–C, in vivo anti-inflammatory meroterpenoids with rearranged carbon skeletons from the marine sponge dysidea septosa. Org. Lett..

[36] Seuter F., Busse W.D. (1979). Arachidonic acid-induced mortality in animals—An appropriate model for the evaluation of antithrombotic drugs?. Agents Actions Suppl..

[37] Gyojeong G., Yirang N., Hyewon C., Hyeok S.S., Hae Y. (2017). Zebrafish larvae model of dilated cardiomyopathy induced by terfenadine. Korean Circ. J..

[38] Zhang X.G., Zhou A.M., Lin X.X., Chen Y.Q., Yang G.M. (2009). Comparative study of chemical compositions of white shrimp head and shell. Mod. Food Sci. Technol..

[39] Li X., Li C., Zhu Y., Shi Y., Zhang X., Zhang S., Wang L., Lin H.W., Hou H., Hsiao C.D. (2020). Lipids fingerprinting of different materials sources by UPLC-Q-exactive orbitrap/MS approach and their zebrafish-based activities comparison. J. Agric. Food Chem..

[40] Li X., He Q., Hou H., Zhang S., Zhang X., Zhang Y., Wang X., Han L., Liu K. (2018). Targeted lipidomics profiling of marine phospholipids from different resources by UPLC-Q-exactive orbitrap/MS approach. J. Chromatogr. B.

[41] Bligh E.G., Dyer W.J. (1959). A rapid method of total lipid extraction and purification. Can. J. Biochem. Physiol..

[42] Dai W., Xie D., Lu M., Li P., Lv H., Yang C., Peng Q., Zhu Y., Guo L., Zhang Y. (2017). Characterization of white tea metabolome: Comparison against green and black tea by a nontargeted metabolomics approach. Food Res. Int..

