# Supplementary material for: Lipid Profiles of the Heads of Four Shrimp Species by UPLC–Q–Exactive Orbitrap/MS and Their Cardiovascular Activities
Source: Molecules. 2022 Jan 6;27(2):350. doi: 10.3390/molecules27020350 (PMC8781101; doi:10.3390/molecules27020350)
Supplement: Supplementary file 1 [file molecules-27-00350-s001.zip › molecules-1510977-supplementary.pdf]

Supplementary Material

Lipid Profiles of the Heads of Four Shrimp Species by UPLC–Q–Exactive Orbitrap/MS and Their Cardiovascular Activities

Yongqiang Zhu [1,2,†], Peihai Li [1,†], Ronghua Meng [3], Xiaobin Li [1,2,*], Yuezi Qiu [1], Lizheng Wang [1], Shanshan Zhang [1], Xuanming Zhang [1], Houwen Lin [4], Hongbin Zhai [5] and Kechun Liu [1,*]

[1] Engineering Research Center of Zebrafish Models for Human Diseases and Drug Screening of Shandong Province, Key Laboratory for Biosensor of Shandong Province, Biology Institute, Qilu University of Technology, Shandong Academy of Sciences, Jinan 250103, China; m17864181498@163.com (Y.Z.); lipeihaihg@sina.com (P.L.); qyz970516@163.com (Y.Q.); wlzh1106@126.com (L.W.); qingshuibaikai@126.com (S.Z.); lenghanxing@163.com (X.Z.)
[2] Bioengineering Technology Innovation Center of Shandong Province, Qilu University of Technology, Shandong Academy of Sciences, Heze 274000, China
[3] Physical and Chemical Examination Division, Zoucheng Center for Disease Control and Prevention, Zoucheng 273500, China; mrh777@163.com
[4] Research Center for Marine Drugs, State Key Laboratory of Oncogenes and Related Genes, Department of Pharmacy, School of Medicine, Shanghai Jiao Tong University, Shanghai 200127, China; franklin67@126.com
[5] Shenzhen Graduate School of Peking University, Shenzhen 518055, China; zhaihb@pku.edu.cn
* Correspondence: lixb@sdas.org (X.L.); hliukch@sdas.org (K.L.)
† These authors contributed equally to this work.

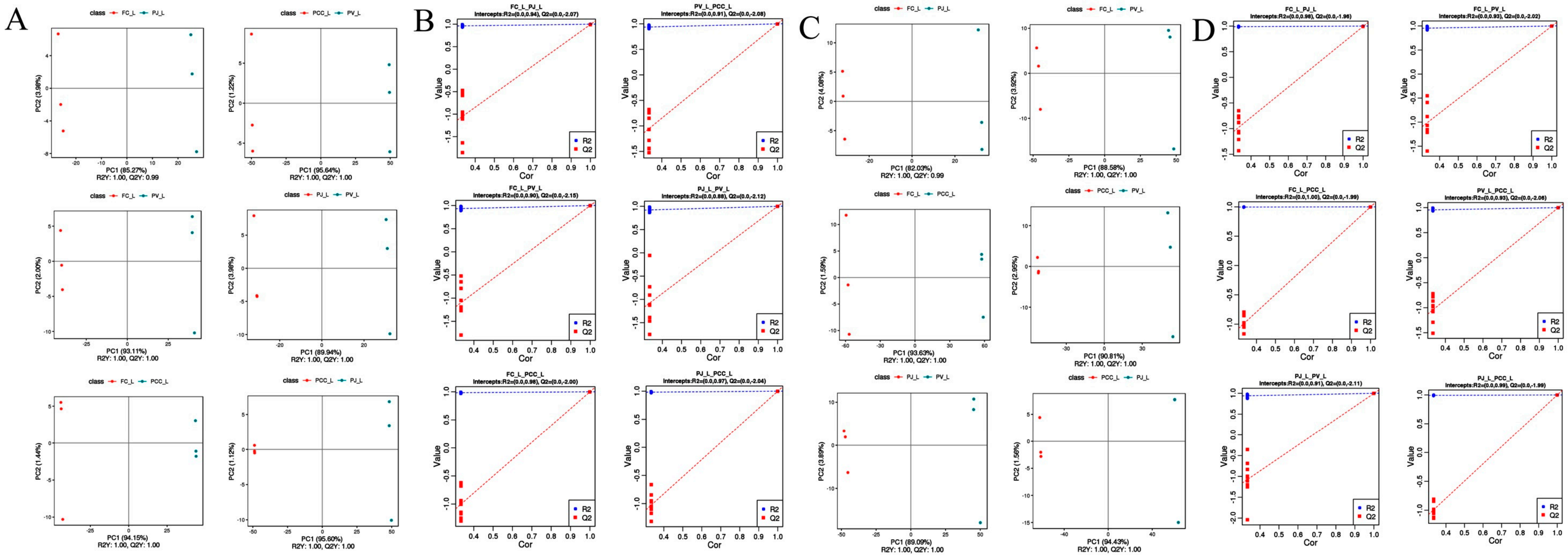

Figure S1. Distribution point diagram of PLS_DA (A, C) and sequencing verification diagram (B,D)(A,B are positive ion modes;C and D are the negative ion modes)

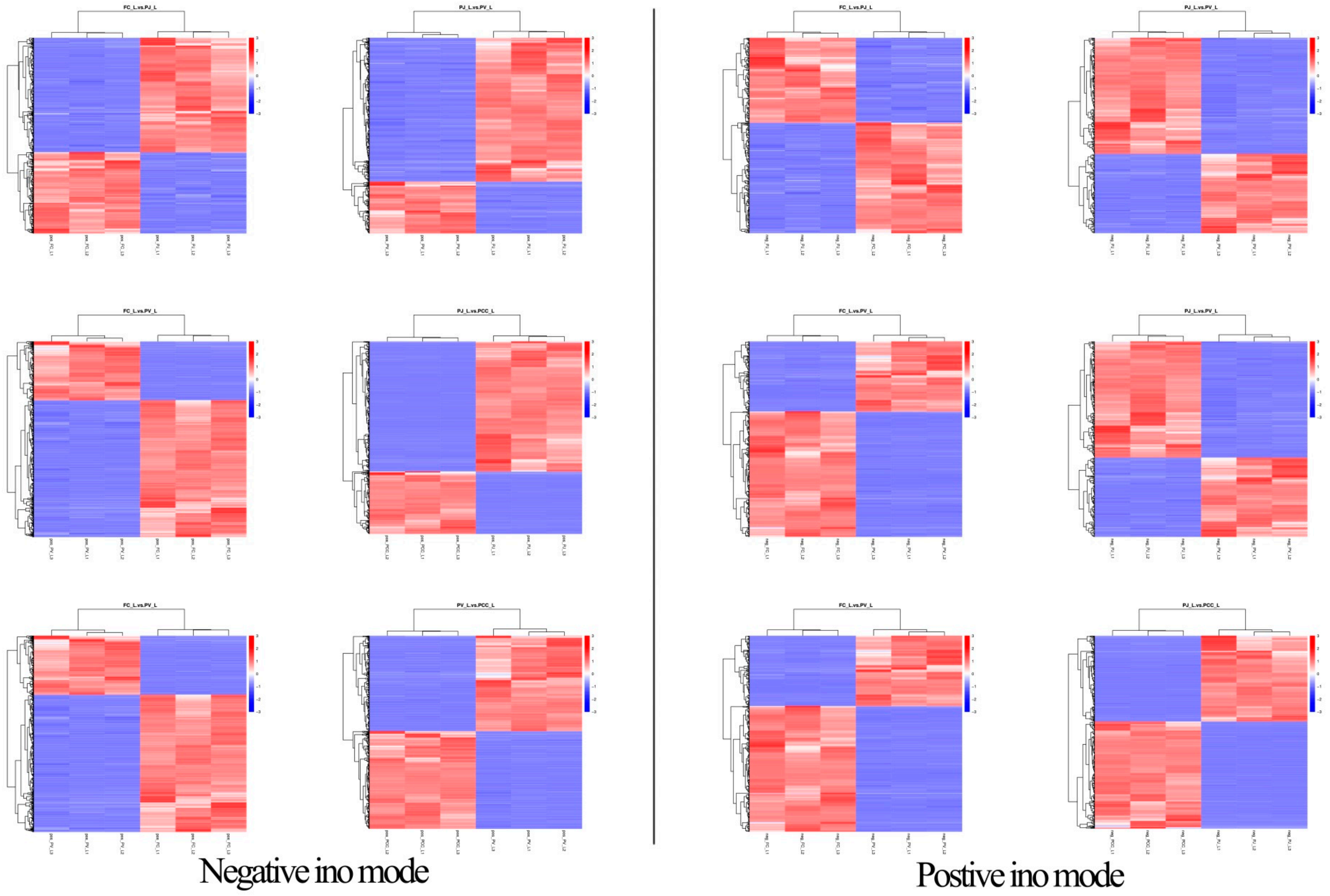

Figure S2. Clustering heat map of different metabolites in each group (negative ion mode on the left and positive ion mode on the right).

Table S1. Lipids Molecular Species of Shrimp Heads Identified By UPLC_Q_Exactive Orbitrap_MS

Molecular species	Exact mass	Molecular Weight		Retention time (min)	Ion mode	Class	ppm	Hit scores	Formula	Peak area percentge (%)			
										FC	PCC	PJ	PV
FAHFA (22:6_6:0)	441.3026123	442.30989	M-H	8.268	neg	Fatty Acyls	3.5726	54.9	C28 H42 O4	0.0005	0.0003	0.0002	0.0002
FAHFA (18:2_2:0)	337.2395325	338.24675	M-H	4.146	neg	Fatty Acyls	3.0760	57	C20 H34 O4	0.0002	0.0045	0.0001	0.0004
FAHFA (16:1_2:0)	311.223999	312.23131	M-H	3.609	neg	Fatty Acyls	4.0050	61.2	C18 H32 O4	0.0002	0.0006	0.0002	0.0001
FAHFA (20:5_5:0)	401.2711487	402.27843	M-H	5.769	neg	Fatty Acyls	3.5307	62.2	C25 H38 O4	0.0003	0.0004	0.0003	0.0001
FAHFA (22:6_5:0)	427.2865906	428.29387	M-H	6.903	neg	Fatty Acyls	2.8257	65.9	C27 H40 O4	0.0004	0.0001	0.0004	0.0001
FAHFA (16:0_18:0)	537.4901733	538.498	M-H	22.658	neg	Fatty Acyls	3.5087	65.9	C34 H66 O4	0.0002	0.0007	0.0001	0.0000
FAHFA (16:0_2:0)	313.2394104	314.24676	M-H	5.814	neg	Fatty Acyls	3.3427	67.6	C18 H34 O4	0.0005	0.0035	0.0003	0.0004
FAHFA (16:0_7:0)	383.3178711	384.32542	M-H	12.478	neg	Fatty Acyls	3.7992	69.8	C23 H44 O4	0.0001	0.0006	0.0001	0.0000
FAHFA (16:1_16:0)	507.4434814	508.45073	M-H	19.923	neg	Fatty Acyls	3.0870	73.7	C32 H60 O4	0.0002	0.0005	0.0000	0.0000
FAHFA (18:1_2:0)	339.2549744	340.26222	M-H	6.279	neg	Fatty Acyls	2.5286	75.8	C20 H36 O4	0.0004	0.0019	0.0003	0.0003
FAHFA (20:1_3:0)	381.3023682	382.30879	M-H	11.310	neg	Fatty Acyls	1.2560	78.6	C23 H42 O4	0.0002	0.0001	0.0002	0.0000
FAHFA (16:0_16:0)	509.4590149	510.46642	M-H	21.374	neg	Fatty Acyls	3.1531	81.4	C32 H62 O4	0.0003	0.0007	0.0000	0.0000
FAHFA (20:5_3:0)	373.2398376	374.24715	M-H	3.653	neg	Fatty Acyls	3.8489	82.6	C23 H34 O4	0.0018	0.0004	0.0018	0.0011
FAHFA (18:2_6:0)	393.3025208	394.30984	M-H	9.821	neg	Fatty Acyls	3.8807	83.5	C24 H42 O4	0.0001	0.0007	0.0001	0.0003
FAHFA (18:2_3:0)	351.2552795	352.26254	M-H	5.627	neg	Fatty Acyls	3.3508	84.1	C21 H36 O4	0.0001	0.0005	0.0000	0.0003
FAHFA (18:1_5:0)	381.3023682	382.3097	M-H	10.657	neg	Fatty Acyls	3.6363	84.2	C23 H42 O4	0.0001	0.0005	0.0001	0.0001
FAHFA (18:1_16:0)	535.4749756	536.48221	M-H	21.452	neg	Fatty Acyls	3.2610	84.6	C34 H64 O4	0.0007	0.0016	0.0001	0.0002
FAHFA (18:1_18:0)	563.5062256	564.51387	M-H	22.704	neg	Fatty Acyls	3.7366	85.3	C36 H68 O4	0.0004	0.0010	0.0001	0.0001
FAHFA (18:1_3:0)	353.2713318	354.27805	M-H	7.957	neg	Fatty Acyls	2.9364	86.3	C21 H38 O4	0.0006	0.0019	0.0005	0.0005
FAHFA (16:0_6:0)	369.302124	370.3094	M-H	11.446	neg	Fatty Acyls	2.9440	88	C22 H42 O4	0.0003	0.0016	0.0002	0.0002
FAHFA (18:1_7:0)	409.3335266	410.34081	M-H	12.636	neg	Fatty Acyls	2.9245	88.6	C25 H46 O4	0.0001	0.0007	0.0000	0.0001
FAHFA (20:4_18:0)	585.4914551	586.49868	M-H	21.576	neg	Fatty Acyls	4.3809	89.8	C38 H66 O4	0.0002	0.0001	0.0000	0.0000
FAHFA (18:2_18:0)	561.4904785	562.4979	M-H	21.829	neg	Fatty Acyls	3.1812	90	C36 H66 O4	0.0001	0.0008	0.0000	0.0001
PC (18:0_20:5)	808.5872803	807.57858	M+H	17.856	pos	glycerophospholipids	0.9594	76.4	C46 H82 N O8 P	3.1921	0.9467	2.1707	1.9726
PC (20:4e_16:0)	768.588562	767.58333	M+H	15.786	pos	glycerophospholipids	0.5725	83	C44 H82 N O7 P	0.0026	0.0047	0.0021	0.0006
PC (15:1_18:2)	742.5374146	741.52953	M+H	18.072	pos	glycerophospholipids	1.7869	75.9	C41 H76 N O8 P	0.0150	0.0057	0.0193	0.0140
PC (19:0_20:3)	826.6342163	825.62157	M+H	12.623	pos	glycerophospholipids	3.8582	71.2	C47 H88 N O8 P	0.0000	0.0001	0.0000	0.0000
PC (16:0e_21:1)	788.6525879	787.64662	M+H	22.002	pos	glycerophospholipids	1.4336	80.2	C45 H90 N O7 P	0.0148	0.0048	0.0203	0.0144

PC (16:0_16:1)	732.5557861	731.54723	M+H	16.643	pos	glycerophospholipids	0.9909	75.3	C40 H78 N O8 P	2.9727	1.9048	2.3967	0.7940
PC (16:0e_19:0)	762.630249	761.63289	M+H	21.825	pos	glycerophospholipids	4.0035	80.5	C43 H88 N O7 P	0.0054	0.0123	0.0048	0.0140
PC (15:1_20:5)	764.538147	763.51695	M+H	12.836	pos	glycerophospholipids	2.2855	72.4	C43 H74 N O8 P	0.0138	0.0024	0.0351	0.0021
PC (18:0e_19:0)	790.6555176	789.66448	M+H	21.337	pos	glycerophospholipids	4.2285	80.9	C45 H92 N O7 P	0.0000	0.0002	0.0000	0.0000
PC (13:0_14:0)	664.4936523	663.48454	M+H	13.163	pos	glycerophospholipids	0.9573	71.5	C35 H70 N O8 P	0.0358	0.0082	0.0357	0.0055
PC (20:3e_11:0)	700.5178833	699.52092	M+H	13.705	pos	glycerophospholipids	0.9001	77.2	C39 H74 N O7 P	0.0006	0.0032	0.0006	0.0009
PC (18:4e_20:3)	790.5766602	789.56784	M+H	15.878	pos	glycerophospholipids	0.7592	82.8	C46 H80 N O7 P	0.1793	0.0815	0.2031	0.0933
PC (15:0_15:1)	704.5244751	703.51863	M+H	16.753	pos	glycerophospholipids	4.8685	69.7	C38 H74 N O8 P	0.2060	0.0926	0.1022	0.0709
PC (21:0_20:5)	850.6310425	849.62672	M+H	20.720	pos	glycerophospholipids	2.3123	74.8	C49 H88 N O8 P	0.0271	0.0044	0.0219	0.0415
PC (20:3_22:5)	858.6002808	857.59368	M+H	16.362	pos	glycerophospholipids	0.2620	73.5	C50 H84 N O8 P	0.0080	0.0046	0.0126	0.0060
PC (20:2_22:6)	858.5874634	857.59394	M+H	17.086	pos	glycerophospholipids	0.5652	78.5	C50 H84 N O8 P	0.1036	0.0272	0.1067	0.0449
PC (14:1e_26:4)	822.6323242	821.6319	M+H	20.814	pos	glycerophospholipids	2.5063	80.4	C48 H88 N O7 P	0.0118	0.0850	0.0188	0.0670
PC (18:0_20:3)	812.6260986	811.60821	M+H	19.668	pos	glycerophospholipids	1.1032	80.5	C46 H86 N O8 P	0.1298	0.0435	0.1017	0.2308
PC (19:0_20:5)	822.5982056	821.59405	M+H	18.892	pos	glycerophospholipids	0.7239	84.5	C47 H84 N O8 P	0.2134	0.0687	0.1657	0.0755
PC (18:1_17:2)	770.5673828	769.56011	M+H	19.652	pos	glycerophospholipids	2.6576	68.8	C43 H80 N O8 P	0.0002	0.0006	0.0002	0.0009
PC (22:5e_12:0)	738.5442505	737.53679	M+H	14.762	pos	glycerophospholipids	1.1519	82.7	C42 H76 N O7 P	0.3009	0.4436	0.8955	0.4458
PC (18:1_20:4)	808.5848999	807.5782	M+H	17.121	pos	glycerophospholipids	0.4889	73.9	C46 H82 N O8 P	1.3562	0.3988	1.1364	0.2642
PC (19:2_18:3)	794.5698242	793.56102	M+H	18.186	pos	glycerophospholipids	1.4304	70.9	C45 H80 N O8 P	0.0027	0.0029	0.0016	0.0018
PC (19:0_18:2)	800.6326294	799.60994	M+H	19.930	pos	glycerophospholipids	1.0438	76.4	C45 H86 N O8 P	0.3808	0.1879	0.5440	0.4333
PC (18:2_19:2)	796.5844727	795.57561	M+H	19.891	pos	glycerophospholipids	2.7593	71.2	C45 H82 N O8 P	0.0019	0.0009	0.0017	0.0018
PC (13:1_18:3)	712.4890747	711.4856	M+H	14.469	pos	glycerophospholipids	2.3826	77.5	C39 H70 N O8 P	0.0007	0.0060	0.0005	0.0005
PC (18:4_18:4)	774.5064697	773.50029	M+H	11.332	pos	glycerophospholipids	0.9504	74.6	C44 H72 N O8 P	0.0019	0.0024	0.0066	0.0006
PC (16:2_16:2)	726.5168457	725.50012	M+H	12.751	pos	glycerophospholipids	0.7789	76.3	C40 H72 N O8 P	0.0353	0.0246	0.0716	0.0072
PC (22:5e_16:1)	792.5926514	791.58312	M+H	15.628	pos	glycerophospholipids	0.2898	83.2	C46 H82 N O7 P	0.0428	0.0181	0.0596	0.0180
PC (21:2_20:4)	848.6151733	847.60897	M+H	21.615	pos	glycerophospholipids	0.1597	70.7	C49 H86 N O8 P	0.0042	0.0003	0.0025	0.0004
PC (16:3_16:3)	722.4778442	721.46997	M+H	10.041	pos	glycerophospholipids	2.3774	70.3	C40 H68 N O8 P	0.0003	0.0015	0.0004	0.0002
PC (20:2_20:2)	838.6419067	837.6258	M+H	20.787	pos	glycerophospholipids	1.2471	77.1	C48 H88 N O8 P	0.0740	0.0473	0.0790	0.0454
PC (21:2_22:6)	872.6186523	871.61009	M+H	16.159	pos	glycerophospholipids	1.1297	70.7	C51 H86 N O8 P	0.0067	0.0005	0.0126	0.0008
PC (16:0e_3:0)	538.3862305	537.37898	M+H	3.939	pos	glycerophospholipids	0.8556	50.7	C27 H56 N O7 P	0.0965	0.0024	0.0341	0.0007
PC (17:0_18:4)	768.5564575	767.54808	M+H	18.096	pos	glycerophospholipids	2.0519	71.1	C43 H78 N O8 P	0.0136	0.0015	0.0019	0.0019
PC (18:1e_13:1)	702.5454102	701.53725	M+H	15.404	pos	glycerophospholipids	1.8668	82.8	C39 H76 N O7 P	0.0100	0.0066	0.0316	0.0024
PC (16:1e_17:2)	728.5506592	727.55297	M+H	16.023	pos	glycerophospholipids	1.8961	82	C41 H78 N O7 P	0.0253	0.1238	0.0458	0.0464

PC (18:4e_19:0)	782.6054077	781.5991	M+H	19.178	pos	glycerophospholipids	0.7156	82.6	C45 H84 N O7 P	0.4288	1.0610	0.3604	2.0596
PC (18:2e_22:6)	816.5923462	815.58487	M+H	16.313	pos	glycerophospholipids	2.4270	81	C48 H82 N O7 P	0.0057	0.0055	0.0052	0.0561
PC (14:1_15:1)	688.4929199	687.48564	M+H	12.838	pos	glycerophospholipids	2.5239	57	C37 H70 N O8 P	0.0002	0.0000	0.0001	0.0000
PC (21:1_21:1)	870.6937866	869.68968	M+H	23.311	pos	glycerophospholipids	2.6726	75.2	C50 H96 N O8 P	0.0119	0.0014	0.0175	0.2947
PC (22:5e_14:1)	764.5608521	763.55238	M+H	14.215	pos	glycerophospholipids	1.0340	81.1	C44 H78 N O7 P	0.0091	0.0044	0.0159	0.0011
PC (17:0_20:4)	796.5841675	795.57808	M+H	18.008	pos	glycerophospholipids	0.3454	76.5	C45 H82 N O8 P	0.5789	0.1853	0.4024	0.1397
PC (20:2_21:2)	852.6470947	851.63762	M+H	21.288	pos	glycerophospholipids	3.2707	63.9	C49 H90 N O8 P	0.0025	0.0014	0.0009	0.0003
PC (14:0e_17:1)	704.555603	703.55287	M+H	16.463	pos	glycerophospholipids	1.8187	80.4	C39 H78 N O7 P	0.0505	0.1301	0.0822	0.0246
PC (15:1_22:6)	790.5401001	789.53141	M+H	17.299	pos	glycerophospholipids	0.7029	76.6	C45 H76 N O8 P	0.0999	0.0183	0.1301	0.0373
PC (22:3e_19:2)	836.6454468	835.64668	M+H	21.057	pos	glycerophospholipids	1.4230	76.6	C49 H90 N O7 P	0.0018	0.0030	0.0036	0.0291
PC (14:0e_22:4)	768.5922241	767.58416	M+H	18.185	pos	glycerophospholipids	1.6538	83.3	C44 H82 N O7 P	0.6581	1.7244	0.7301	0.9226
PC (22:6_22:6)	878.5662842	877.56265	M+H	14.460	pos	glycerophospholipids	0.5639	82.9	C52 H80 N O8 P	0.5799	0.0143	0.7852	0.2706
PC (16:0e_19:1)	760.6112671	759.61556	M+H	20.578	pos	glycerophospholipids	1.8026	83.3	C43 H86 N O7 P	0.0550	0.0793	0.0590	0.0773
PC (16:1_16:4)	724.4906616	723.48362	M+H	12.949	pos	glycerophospholipids	0.3937	51.4	C40 H70 N O8 P	0.0006	0.0009	0.0008	0.0009
PC (22:5e_11:0)	724.5296021	723.52137	M+H	13.726	pos	glycerophospholipids	1.4922	81.4	C41 H74 N O7 P	0.0390	0.1639	0.0770	0.0614
PC (19:0_20:2)	828.6374512	827.64001	M+H	21.078	pos	glycerophospholipids	0.4778	70.8	C47 H90 N O8 P	0.2547	0.0039	0.1055	0.0388
PC (20:0_21:2)	856.6738281	855.67213	M+H	22.301	pos	glycerophospholipids	0.4960	69.1	C49 H94 N O8 P	0.5219	0.0134	0.2826	0.1181
PC (22:4e_10:0)	712.5175171	711.52142	M+H	14.657	pos	glycerophospholipids	1.5877	82.4	C40 H74 N O7 P	0.0131	0.0405	0.0411	0.0102
PC (22:0_24:4)	922.7280273	921.72013	M+H	23.362	pos	glycerophospholipids	1.5994	67.5	C54 H100 N O8 P	0.0118	0.0064	0.0150	0.0761
PC (18:3e_21:2)	808.6205444	807.61425	M+H	18.663	pos	glycerophospholipids	0.0734	79	C47 H86 N O7 P	0.0042	0.0005	0.0020	0.0017
PC (18:0e_11:0)	678.5453491	677.53746	M+H	16.483	pos	glycerophospholipids	2.2428	81.5	C37 H76 N O7 P	0.0116	0.0834	0.0146	0.0078
PC (18:5e_19:2)	776.5606689	775.55339	M+H	16.042	pos	glycerophospholipids	2.3203	82.1	C45 H78 N O7 P	0.0251	0.0132	0.0516	0.0121
PC (22:3_26:4)	944.7093506	943.70294	M+H	22.941	pos	glycerophospholipids	0.0696	60	C56 H98 N O8 P	0.0167	0.0003	0.0123	0.0031
PC (22:3e_17:1)	810.6390991	809.6315	M+H	20.949	pos	glycerophospholipids	2.0493	74.3	C47 H88 N O7 P	0.0025	0.0524	0.0031	0.0218
PC (22:1_22:1)	898.7228394	897.71948	M+H	23.770	pos	glycerophospholipids	0.9181	74.6	C52 H100 N O8 P	0.5645	0.0141	0.7870	0.4892
PC (14:0e_18:1)	718.5745239	717.56702	M+H	17.102	pos	glycerophospholipids	0.3073	83.8	C40 H80 N O7 P	0.0586	0.0021	0.0478	0.0007
PC (18:0_19:1)	802.6323242	801.6254	M+H	21.089	pos	glycerophospholipids	0.8041	71.2	C45 H88 N O8 P	0.9679	0.2063	0.7515	0.3535
PC (20:1_21:2)	854.6665649	853.65766	M+H	21.405	pos	glycerophospholipids	1.8795	63	C49 H92 N O8 P	0.0180	0.0050	0.0158	0.0291
PC (14:0_16:3)	700.4897461	699.48564	M+H	11.621	pos	glycerophospholipids	2.4806	71.1	C38 H70 N O8 P	0.0004	0.0122	0.0013	0.0007
PC (14:1e_15:0)	676.5216675	675.52125	M+H	15.432	pos	glycerophospholipids	1.4206	81.5	C37 H74 N O7 P	0.0036	0.0063	0.0040	0.0035
PC (22:6e_16:3)	786.5446777	785.53654	M+H	14.037	pos	glycerophospholipids	0.7633	76	C46 H76 N O7 P	0.0040	0.0033	0.0207	0.0239
PC (14:0e_22:3)	770.5975952	769.59852	M+H	18.391	pos	glycerophospholipids	0.0268	82.7	C44 H84 N O7 P	0.0010	0.0098	0.0013	0.0137

PC (18:5e_2:0)	542.3256836	541.31801	M+H	14.596	pos	glycerophospholipids	2.1623	79.2	C28 H48 N O7 P	0.0004	0.0008	0.0013	0.0009
PC (18:5e_3:0)	556.3410645	555.33326	M+H	2.053	pos	glycerophospholipids	1.3873	67.3	C29 H50 N O7 P	0.0175	0.0133	0.0312	0.0106
PC (22:3e_10:0)	714.5454712	713.53819	M+H	15.620	pos	glycerophospholipids	3.1527	82.8	C40 H76 N O7 P	0.0051	0.0063	0.0200	0.0017
PC (20:1_20:1)	842.6625366	841.65628	M+H	21.775	pos	glycerophospholipids	0.2667	77.3	C48 H92 N O8 P	0.9712	0.0249	0.9154	0.4784
PC (17:2_16:3)	738.5092773	737.50172	M+H	14.964	pos	glycerophospholipids	2.9357	69.8	C41 H72 N O8 P	0.0004	0.0618	0.0002	0.0033
PC (19:1_18:2)	798.5997925	797.59459	M+H	18.221	pos	glycerophospholipids	1.4227	73.3	C45 H84 N O8 P	0.0536	0.0527	0.0575	0.0925
PC (17:0_18:3)	770.567627	769.564	M+H	16.882	pos	glycerophospholipids	2.3973	81.7	C43 H80 N O8 P	0.0297	0.2147	0.0164	0.0865
PC (18:4e_16:4)	732.4956055	731.49024	M+H	10.936	pos	glycerophospholipids	1.7086	78.1	C42 H70 N O7 P	0.0003	0.0001	0.0030	0.0000
PC (21:2_20:3)	850.6264038	849.62381	M+H	19.670	pos	glycerophospholipids	1.1127	70.7	C49 H88 N O8 P	0.0429	0.0008	0.0160	0.0011
PC (20:5e_14:1)	736.5203857	735.52105	M+H	13.670	pos	glycerophospholipids	1.0328	79.4	C42 H74 N O7 P	0.0296	0.0083	0.0737	0.0175
PC (13:1_22:6)	762.5075684	761.50031	M+H	15.219	pos	glycerophospholipids	0.9916	72.6	C43 H72 N O8 P	0.0240	0.0046	0.0561	0.0046
PC (14:0_14:0)	678.5060425	677.4995	M+H	14.225	pos	glycerophospholipids	0.0810	81.1	C36 H72 N O8 P	0.2053	0.0650	0.2016	0.0614
PC (22:6e_15:1)	776.560791	775.55277	M+H	18.435	pos	glycerophospholipids	1.5209	83.9	C45 H78 N O7 P	0.0015	0.0004	0.0004	0.0015
PC (20:5_22:5)	854.571167	853.5642	M+H	14.206	pos	glycerophospholipids	2.3957	75.1	C50 H80 N O8 P	0.0617	0.0063	0.2567	0.0366
PC (17:0_20:5)	794.5718384	793.56318	M+H	16.795	pos	glycerophospholipids	1.2915	78.6	C45 H80 N O8 P	0.8160	0.2158	0.5367	0.4889
PC (17:1_17:1)	758.5608521	757.56368	M+H	17.114	pos	glycerophospholipids	2.0128	77.2	C42 H80 N O8 P	0.8186	4.1931	0.9216	4.4138
PC (14:0e_16:0)	692.5585938	691.55128	M+H	15.229	pos	glycerophospholipids	0.4489	82.3	C38 H78 N O7 P	0.0011	0.0026	0.0053	0.0009
PC (18:4e_22:0)	824.651001	823.64697	M+H	21.649	pos	glycerophospholipids	1.7959	76	C48 H90 N O7 P	0.0014	0.0372	0.0023	0.0079
PC (18:1_18:2)	784.5878296	783.57944	M+H	17.311	pos	glycerophospholipids	2.0863	72.9	C44 H82 N O8 P	0.2294	3.1410	0.2686	3.0589
PC (17:1_22:4)	822.6038818	821.59661	M+H	17.351	pos	glycerophospholipids	3.8398	71.7	C47 H84 N O8 P	0.0011	0.0018	0.0016	0.0014
PC (20:3_20:3)	834.6008301	833.59581	M+H	18.215	pos	glycerophospholipids	2.8248	77.1	C48 H84 N O8 P	0.2690	0.0166	0.3097	0.0308
PC (18:0_18:1)	788.6056519	787.60988	M+H	20.383	pos	glycerophospholipids	0.9836	75.7	C44 H86 N O8 P	4.3175	1.4407	4.3228	3.2280
PC (18:5e_20:3)	788.5608521	787.55339	M+H	14.108	pos	glycerophospholipids	2.2850	79	C46 H78 N O7 P	0.0015	0.0125	0.0036	0.0656
PC (24:0_20:5)	892.6811523	891.67143	M+H	22.762	pos	glycerophospholipids	0.3091	67.5	C52 H94 N O8 P	0.0406	0.0031	0.0369	0.0524
PC (16:0_22:0)	818.6629639	817.65633	M+H	22.807	pos	glycerophospholipids	0.3357	78.3	C46 H92 N O8 P	0.0408	0.0024	0.0331	0.0376
PC (19:0_19:1)	816.6494751	815.63963	M+H	22.470	pos	glycerophospholipids	0.9507	70.3	C46 H90 N O8 P	0.0759	0.0014	0.0101	0.0048
PC (19:2_19:2)	810.6004639	809.59411	M+H	19.012	pos	glycerophospholipids	0.8087	77.6	C46 H84 N O8 P	1.7579	0.9873	1.2092	0.8369
PC (20:1_22:6)	860.6022339	859.60939	M+H	19.002	pos	glycerophospholipids	0.3311	78.8	C50 H86 N O8 P	0.2148	0.0092	0.3428	0.0086
PC (18:1_26:2)	896.7124023	895.70334	M+H	23.138	pos	glycerophospholipids	0.3732	73.5	C52 H98 N O8 P	0.0304	0.0039	0.0568	0.2201
PC (4:0_16:3)	560.3365479	559.32933	M+H	1.258	pos	glycerophospholipids	3.4431	62.7	C28 H50 N O8 P	0.0040	0.0096	0.0033	0.0008
PC (14:0e_13:0)	650.5117798	649.5045	M+H	14.386	pos	glycerophospholipids	0.2160	75.5	C35 H72 N O7 P	0.0015	0.0026	0.0011	0.0008
PC (20:5e_16:4)	758.4987183	757.5046	M+H	11.767	pos	glycerophospholipids	0.0532	82.3	C44 H72 N O7 P	0.0318	0.0012	0.2951	0.0053

PC (22:6e_18:3)	814.5638428	813.56828	M+H	15.898	pos	glycerophospholipids	1.2777	80.1	C48 H80 N O7 P	0.1581	0.0072	0.1506	0.0272
PC (18:3e_17:2)	752.5574951	751.55251	M+H	15.865	pos	glycerophospholipids	1.2235	83.1	C43 H78 N O7 P	0.3529	1.8164	0.3417	0.8151
PC (22:4e_14:1)	766.5671997	765.56715	M+H	14.499	pos	glycerophospholipids	0.1183	83.2	C44 H80 N O7 P	0.0168	0.0022	0.0214	0.0011
PC (17:1_17:2)	756.5531616	755.54636	M+H	13.648	pos	glycerophospholipids	0.1920	70.4	C42 H78 N O8 P	0.0014	0.0027	0.0034	0.0011
PC (13:0_18:3)	714.5084229	713.50148	M+H	12.670	pos	glycerophospholipids	2.6981	71.9	C39 H72 N O8 P	0.0006	0.0068	0.0020	0.0016
PC (14:0_16:4)	698.4752808	697.46903	M+H	10.646	pos	glycerophospholipids	1.1115	68.2	C38 H68 N O8 P	0.0002	0.0006	0.0010	0.0001
PC (18:2e_16:0)	744.5921021	743.58245	M+H	17.268	pos	glycerophospholipids	0.5925	82.6	C42 H82 N O7 P	0.0232	0.0004	0.0161	0.0031
PC (14:0e_18:3)	714.545166	713.53703	M+H	14.557	pos	glycerophospholipids	1.5270	78.6	C40 H76 N O7 P	0.0013	0.0217	0.0037	0.0016
PC (20:4_22:5)	856.5869751	855.57746	M+H	13.947	pos	glycerophospholipids	0.4035	75.5	C50 H82 N O8 P	0.0212	0.0004	0.0244	0.0014
PC (17:0_17:0)	762.5908813	761.59439	M+H	20.189	pos	glycerophospholipids	1.2273	77.3	C42 H84 N O8 P	0.5713	0.1739	0.4717	0.8618
PC (14:0_15:1)	690.508728	689.50024	M+H	13.470	pos	glycerophospholipids	0.9936	72.1	C37 H72 N O8 P	0.0149	0.0063	0.0508	0.0045
PC (15:0_16:0)	720.5425415	719.54705	M+H	17.360	pos	glycerophospholipids	0.7573	82.6	C39 H78 N O8 P	0.4843	0.1672	0.1942	0.1398
PC (14:0e_16:2)	688.5375977	687.52342	M+H	13.114	pos	glycerophospholipids	4.5521	58.3	C38 H74 N O7 P	0.0001	0.0000	0.0000	0.0000
PC (19:0_20:0)	832.6670532	831.67291	M+H	23.336	pos	glycerophospholipids	1.4482	67.3	C47 H94 N O8 P	0.0169	0.0024	0.0131	0.0273
PC (22:6e_20:5)	838.5614014	837.56711	M+H	14.368	pos	glycerophospholipids	0.1558	80.9	C50 H80 N O7 P	0.0302	0.0027	0.0083	0.0009
PC (18:3e_22:0)	826.6674805	825.66417	M+H	20.823	pos	glycerophospholipids	3.6687	79.8	C48 H92 N O7 P	0.0034	0.0004	0.0030	0.0010
PC (18:0_20:1)	816.6370239	815.64183	M+H	21.810	pos	glycerophospholipids	1.7465	75.8	C46 H90 N O8 P	0.4518	0.0838	0.5038	0.3700
PC (22:3e_16:2)	794.6068115	793.59863	M+H	18.842	pos	glycerophospholipids	0.1126	81.1	C46 H84 N O7 P	0.0018	0.0036	0.0015	0.0019
PC (14:1e_24:4)	794.602356	793.59998	M+H	19.877	pos	glycerophospholipids	1.8137	83.4	C46 H84 N O7 P	0.0866	0.5503	0.0950	0.0709
PC (15:0_16:4)	712.4925537	711.48136	M+H	14.118	pos	glycerophospholipids	3.5768	71.4	C39 H70 N O8 P	0.0002	0.0005	0.0001	0.0003
PC (17:1_18:1)	772.5847168	771.57816	M+H	17.762	pos	glycerophospholipids	0.4598	76.6	C43 H82 N O8 P	0.2805	0.4926	0.2236	0.2197
PC (16:1_17:2)	742.5368042	741.53169	M+H	15.049	pos	glycerophospholipids	1.1260	71.9	C41 H76 N O8 P	0.0925	0.0776	0.0412	0.0651
PC (16:0e_15:0)	706.5846558	705.56779	M+H	16.220	pos	glycerophospholipids	0.7788	83.1	C39 H80 N O7 P	0.0005	0.0025	0.0007	0.0027
PC (18:0e_13:1)	704.5586548	703.55183	M+H	14.778	pos	glycerophospholipids	0.3405	83.1	C39 H78 N O7 P	0.0006	0.0003	0.0006	0.0001
PC (22:5e_19:2)	832.6206055	831.61333	M+H	17.641	pos	glycerophospholipids	1.0350	76.8	C49 H86 N O7 P	0.0005	0.0000	0.0001	0.0001
PC (15:0_16:1)	718.5291748	717.532	M+H	15.570	pos	glycerophospholipids	1.5957	76.6	C39 H76 N O8 P	0.7669	0.2887	0.7901	0.1184
PC (22:3e_2:0)	602.4197388	601.41194	M+H	7.616	pos	glycerophospholipids	1.9955	61.9	C32 H60 N O7 P	0.0006	0.0001	0.0003	0.0007
PC (16:0_19:0)	776.6051636	775.61028	M+H	20.970	pos	glycerophospholipids	1.5145	73.6	C43 H86 N O8 P	0.1603	0.0316	0.1209	0.1276
PC (18:3e_22:5)	816.5772095	815.58388	M+H	17.527	pos	glycerophospholipids	1.2131	79.5	C48 H82 N O7 P	0.0154	0.0098	0.0235	0.0208
PC (21:2_21:2)	866.6742554	865.65799	M+H	21.047	pos	glycerophospholipids	2.2347	75.8	C50 H92 N O8 P	0.0206	0.0039	0.0143	0.2495
PC (20:5e_16:3)	760.5141602	759.51976	M+H	13.102	pos	glycerophospholipids	0.6983	81.9	C44 H74 N O7 P	0.0286	0.0022	0.1890	0.0053
PC (17:0_20:2)	800.6181641	799.60823	M+H	19.603	pos	glycerophospholipids	1.0947	74.5	C45 H86 N O8 P	0.4291	0.1268	0.3489	0.2992

PC (22:3_22:3)	890.6629639	889.65683	M+H	21.283	pos	glycerophospholipids	0.8705	70.7	C52 H92 N O8 P	0.1391	0.0019	0.1609	0.0740
PC (6:0_16:3)	588.3677368	587.36046	M+H	1.446	pos	glycerophospholipids	2.9891	63.4	C30 H54 N O8 P	0.0018	0.0169	0.0026	0.0007
PC (16:0_18:1)	760.5871582	759.57893	M+H	18.548	pos	glycerophospholipids	1.4808	80.2	C42 H82 N O8 P	5.0062	7.2148	4.0965	5.2437
PC (16:1e_26:4)	850.6577759	849.66263	M+H	22.235	pos	glycerophospholipids	1.7526	74.7	C50 H92 N O7 P	0.0035	0.0099	0.0037	0.0089
PC (20:4_20:5)	828.5579224	827.54723	M+H	14.014	pos	glycerophospholipids	0.8760	74.8	C48 H78 N O8 P	1.0594	0.3582	1.6734	0.6060
PC (22:6e_18:0)	820.6092529	819.61501	M+H	18.096	pos	glycerophospholipids	0.9996	82.8	C48 H86 N O7 P	0.0205	0.0062	0.0091	0.0396
PC (14:1_14:1)	674.4772949	673.4685	M+H	11.816	pos	glycerophospholipids	0.3641	76.7	C36 H68 N O8 P	0.0107	0.0009	0.0041	0.0032
PC (22:6e_20:1)	846.6246948	845.63062	M+H	19.719	pos	glycerophospholipids	0.9215	83.4	C50 H88 N O7 P	0.0311	0.0047	0.0939	0.0346
PC (18:3_20:4)	804.5541382	803.54625	M+H	16.910	pos	glycerophospholipids	0.3175	54.7	C46 H78 N O8 P	0.0043	0.0064	0.0013	0.0013
PC (18:2e_15:0)	730.5765381	729.56915	M+H	16.973	pos	glycerophospholipids	2.6173	84.4	C41 H80 N O7 P	0.0130	0.3194	0.0202	0.0388
PC (17:0_17:1)	760.5872192	759.58027	M+H	13.316	pos	glycerophospholipids	3.2449	71	C42 H82 N O8 P	0.0004	0.0022	0.0002	0.0011
PC (22:3_24:4)	916.6782227	915.67231	M+H	20.658	pos	glycerophospholipids	0.6601	57	C54 H94 N O8 P	0.0015	0.0036	0.0050	0.0013
PC (22:5_22:6)	880.5720215	879.57811	M+H	15.748	pos	glycerophospholipids	0.3465	78.9	C52 H82 N O8 P	0.0320	0.0028	0.0221	0.0042
PC (20:3e_22:6)	842.6143188	841.59957	M+H	17.986	pos	glycerophospholipids	1.2231	80.6	C50 H84 N O7 P	0.0522	0.0034	0.0682	0.0054
PC (18:5e_18:0)	766.5768433	765.56874	M+H	17.863	pos	glycerophospholipids	1.9586	81.2	C44 H80 N O7 P	0.0585	0.2937	0.0387	0.0366
PC (20:3e_8:0)	658.482666	657.47546	M+H	13.227	pos	glycerophospholipids	3.2242	56.6	C36 H68 N O7 P	0.0009	0.0003	0.0008	0.0008
PC (18:1_18:1)	786.5986328	785.59483	M+H	19.168	pos	glycerophospholipids	1.7499	77.4	C44 H84 N O8 P	0.5937	1.6675	0.6942	4.1497
PC (14:0_14:1)	676.4887695	675.48149	M+H	13.452	pos	glycerophospholipids	3.5750	65.1	C36 H70 N O8 P	0.0001	0.0000	0.0001	0.0000
PC (20:1_18:2)	812.6188354	811.60826	M+H	19.030	pos	glycerophospholipids	1.0416	73.4	C46 H86 N O8 P	0.0814	0.3449	0.1635	0.5646
PC (14:1_18:1)	730.538208	729.53057	M+H	18.385	pos	glycerophospholipids	0.3907	77.3	C40 H76 N O8 P	0.0043	0.0083	0.0020	0.0007
PC (22:5e_15:0)	780.5925903	779.58453	M+H	17.645	pos	glycerophospholipids	2.1029	82.7	C45 H82 N O7 P	0.1217	0.1667	0.1296	0.2420
PC (16:1e_21:1)	786.6393433	785.62712	M+H	19.957	pos	glycerophospholipids	3.4632	82.2	C45 H88 N O7 P	0.0349	0.0041	0.0438	0.0067
PC (18:5e_22:6)	810.543396	809.53779	M+H	13.052	pos	glycerophospholipids	2.2848	81.3	C48 H76 N O7 P	0.0038	0.0010	0.0101	0.0024
PC (14:1e_24:2)	798.637085	797.62686	M+H	19.438	pos	glycerophospholipids	3.7371	82.4	C46 H88 N O7 P	0.2325	0.0052	0.1697	0.0049
PC (13:0_14:1)	662.477478	661.46932	M+H	11.395	pos	glycerophospholipids	1.6104	67.7	C35 H68 N O8 P	0.0021	0.0009	0.0054	0.0007
PC (22:1_24:4)	920.7099609	919.7003	M+H	22.625	pos	glycerophospholipids	2.9420	63.2	C54 H98 N O8 P	0.0035	0.0004	0.0009	0.0003
PC (21:0_22:3)	882.690918	881.68901	M+H	22.606	pos	glycerophospholipids	1.8763	65.1	C51 H96 N O8 P	0.0150	0.0411	0.0164	0.0337
PC (24:1_20:4)	892.6811523	891.67208	M+H	22.010	pos	glycerophospholipids	0.4199	71.3	C52 H94 N O8 P	0.0645	0.0029	0.0897	0.0300
PC (19:1_20:5)	820.5778809	819.58094	M+H	19.659	pos	glycerophospholipids	3.8249	71.2	C47 H82 N O8 P	0.0011	0.0006	0.0017	0.0006
PC (22:6e_20:4)	840.5890503	839.58159	M+H	15.960	pos	glycerophospholipids	1.5491	76.5	C50 H82 N O7 P	0.0039	0.0017	0.0007	0.0003
PC (16:3_18:3)	750.5044556	749.49922	M+H	13.737	pos	glycerophospholipids	0.4468	67.1	C42 H72 N O8 P	0.0020	0.0053	0.0010	0.0033
PC (15:0_15:0)	706.5274048	705.53337	M+H	15.979	pos	glycerophospholipids	3.5647	77.8	C38 H76 N O8 P	0.0499	0.0189	0.0326	0.0200

PC (22:6e_17:2)	802.5772705	801.567	M+H	18.916	pos	glycerophospholipids	0.3001	79	C47 H80 N O7 P	0.0001	0.0013	0.0001	0.0001
PC (16:1e_19:2)	756.5812378	755.58458	M+H	19.080	pos	glycerophospholipids	2.2359	78.9	C43 H82 N O7 P	0.0054	0.0847	0.0056	0.0132
PC (19:1_20:4)	822.5997925	821.59359	M+H	18.154	pos	glycerophospholipids	0.1640	74.2	C47 H84 N O8 P	0.3729	0.0281	0.2184	0.0651
PC (22:3e_14:0)	770.5975952	769.60064	M+H	19.215	pos	glycerophospholipids	2.7278	79.4	C44 H84 N O7 P	0.0081	0.1411	0.0082	0.0261
PC (16:0_20:3)	784.5865479	783.58025	M+H	11.251	pos	glycerophospholipids	3.1200	71.7	C44 H82 N O8 P	0.0000	0.0001	0.0001	0.0000
PC (22:1_22:6)	888.6469116	887.64069	M+H	20.430	pos	glycerophospholipids	0.3205	76.2	C52 H90 N O8 P	0.0279	0.0010	0.0382	0.0057
PC (19:0_22:5)	850.6342163	849.62495	M+H	20.090	pos	glycerophospholipids	0.2290	76	C49 H88 N O8 P	0.0737	0.0029	0.0609	0.0161
PC (18:5e_21:2)	804.5921631	803.5843	M+H	18.470	pos	glycerophospholipids	1.7539	81.9	C47 H82 N O7 P	0.0123	0.0172	0.0282	0.0058
PC (19:0_22:4)	852.6467285	851.64063	M+H	19.215	pos	glycerophospholipids	0.2636	69.9	C49 H90 N O8 P	0.0194	0.0004	0.0099	0.0034
PC (18:3e_19:2)	780.5892334	779.58385	M+H	17.310	pos	glycerophospholipids	1.2307	83	C45 H82 N O7 P	0.2097	2.2290	0.2808	1.5264
PC (21:0_21:1)	872.7125854	871.70397	M+H	23.568	pos	glycerophospholipids	1.1062	69	C50 H98 N O8 P	0.0018	0.0005	0.0013	0.0005
PC (20:4_20:4)	830.5714722	829.56531	M+H	10.106	pos	glycerophospholipids	3.8030	74.7	C48 H80 N O8 P	0.0000	0.0000	0.0000	0.0000
PC (18:1_24:1)	870.696167	869.68792	M+H	22.817	pos	glycerophospholipids	0.6489	78	C50 H96 N O8 P	1.9355	0.0241	1.3879	1.2475
PC (18:4e_16:3)	734.5114136	733.50541	M+H	17.442	pos	glycerophospholipids	1.0494	79.7	C42 H72 N O7 P	0.0018	0.0009	0.0015	0.0004
PC (20:4_22:6)	854.5847168	853.56297	M+H	14.859	pos	glycerophospholipids	0.9546	75	C50 H80 N O8 P	0.6840	0.1124	0.8246	0.3899
PC (22:6e_4:0)	624.4022827	623.39501	M+H	5.005	pos	glycerophospholipids	0.1281	69.1	C34 H58 N O7 P	0.0001	0.0001	0.0030	0.0001
PC (23:0_18:1)	858.6968384	857.68813	M+H	23.444	pos	glycerophospholipids	0.9028	78.7	C49 H96 N O8 P	0.0591	0.0035	0.0834	0.1227
PC (22:3e_4:0)	630.4512939	629.44327	M+H	10.776	pos	glycerophospholipids	1.9541	59.4	C34 H64 N O7 P	0.0008	0.0002	0.0005	0.0012
PC (20:0_20:5)	836.6154175	835.61007	M+H	16.221	pos	glycerophospholipids	1.1544	74.9	C48 H86 N O8 P	0.0021	0.0019	0.0090	0.0015
PC (13:1_20:5)	736.4907227	735.48456	M+H	14.344	pos	glycerophospholipids	0.8908	70.5	C41 H70 N O8 P	0.0171	0.0064	0.0506	0.0025
PC (12:0_13:0)	636.461792	635.4525	M+H	11.004	pos	glycerophospholipids	0.1648	68.1	C33 H66 N O8 P	0.0015	0.0002	0.0068	0.0002
PC (22:2_22:2)	894.6852417	893.68911	M+H	22.317	pos	glycerophospholipids	1.9630	71.8	C52 H96 N O8 P	0.0423	0.0057	0.0263	0.2114
PC (19:2_22:6)	844.5864258	843.57735	M+H	13.801	pos	glycerophospholipids	0.5396	70.9	C49 H82 N O8 P	0.0152	0.0031	0.0095	0.0023
PC (18:2_22:6)	830.5841064	829.56361	M+H	15.204	pos	glycerophospholipids	1.7538	79.1	C48 H80 N O8 P	0.5390	0.6085	0.8508	2.4318
PC (22:3e_25:0)	924.7744141	923.76714	M+H	24.256	pos	glycerophospholipids	3.8444	56.3	C55 H106 N O7 P	0.0002	0.0014	0.0001	0.0001
PC (14:1e_21:1)	758.6074219	757.60055	M+H	20.345	pos	glycerophospholipids	2.6523	83	C43 H84 N O7 P	0.0208	0.1046	0.0198	0.0077
PC (18:4e_19:2)	778.5765991	777.56857	M+H	16.704	pos	glycerophospholipids	1.7098	83	C45 H80 N O7 P	0.0899	0.0963	0.1024	0.1577
PC (18:2e_20:0)	800.6547852	799.64655	M+H	21.507	pos	glycerophospholipids	1.3245	82	C46 H90 N O7 P	0.0103	0.0088	0.0192	0.0561
PC (19:0_18:1)	802.6212158	801.6246	M+H	19.444	pos	glycerophospholipids	0.1939	74.4	C45 H88 N O8 P	0.2680	0.0075	0.1864	0.0033
PC (16:0_19:2)	772.5830078	771.57573	M+H	11.351	pos	glycerophospholipids	2.6896	72.5	C43 H82 N O8 P	0.0000	0.0000	0.0000	0.0000
PC (15:0_16:3)	714.5089111	713.50115	M+H	15.250	pos	glycerophospholipids	2.2356	70.7	C39 H72 N O8 P	0.0116	0.0209	0.0181	0.0034
PC (13:0_16:3)	686.4738159	685.46654	M+H	8.306	pos	glycerophospholipids	2.5016	75.4	C37 H68 N O8 P	0.0001	0.0002	0.0000	0.0000

PC (18:4e_13:0)	698.5223389	697.50514	M+H	13.540	pos	glycerophospholipids	0.7164	79.5	C39 H72 N O7 P	0.0009	0.0013	0.0039	0.0005
PC (16:1e_21:2)	784.6176758	783.6162	M+H	19.859	pos	glycerophospholipids	2.5641	77.9	C45 H86 N O7 P	0.0026	0.0047	0.0018	0.0085
PC (24:1_22:6)	916.6780396	915.6719	M+H	21.795	pos	glycerophospholipids	0.2123	61.4	C54 H94 N O8 P	0.0912	0.0007	0.0435	0.0158
PC (11:0_20:5)	710.4777222	709.46932	M+H	10.458	pos	glycerophospholipids	1.5015	69	C39 H68 N O8 P	0.0003	0.0009	0.0026	0.0003
PC (20:0_22:5)	864.642334	863.64094	M+H	21.428	pos	glycerophospholipids	0.6189	70.1	C50 H90 N O8 P	0.0791	0.0066	0.0598	0.0584
PC (18:0e_12:0)	692.5608521	691.55278	M+H	17.531	pos	glycerophospholipids	1.7201	82.3	C38 H78 N O7 P	0.0660	0.2086	0.0929	0.1309
PC (18:5e_18:5)	756.4829102	755.48693	M+H	11.973	pos	glycerophospholipids	2.7270	81	C44 H70 N O7 P	0.0002	0.0001	0.0016	0.0001
PC (21:2_20:5)	846.5953369	845.59429	M+H	15.089	pos	glycerophospholipids	0.9871	70.4	C49 H84 N O8 P	0.0312	0.0044	0.0263	0.0227
PC (16:1e_9:0)	620.4643555	619.45805	M+H	13.043	pos	glycerophospholipids	0.5810	69.2	C33 H66 N O7 P	0.0097	0.0005	0.0055	0.0027
PC (22:6e_14:1)	762.5332031	761.53668	M+H	15.354	pos	glycerophospholipids	0.9712	74.6	C44 H76 N O7 P	0.0085	0.0086	0.0146	0.0058
PC (16:1_22:6)	804.5571289	803.54564	M+H	15.355	pos	glycerophospholipids	1.0766	78.1	C46 H78 N O8 P	0.2485	0.0101	0.2081	0.0363
PC (18:1e_14:0)	718.5762329	717.56845	M+H	17.803	pos	glycerophospholipids	1.6855	84.8	C40 H80 N O7 P	0.4439	0.8053	0.4261	0.0742
PC (18:4_18:5)	772.4883423	771.48133	M+H	12.350	pos	glycerophospholipids	3.3375	70.7	C44 H70 N O8 P	0.0005	0.0001	0.0036	0.0002
PC (22:4e_19:2)	834.6482544	833.63016	M+H	18.938	pos	glycerophospholipids	0.3829	82.5	C49 H88 N O7 P	0.0003	0.0208	0.0006	0.0006
PC (16:1e_18:2)	742.5650635	741.5689	M+H	16.468	pos	glycerophospholipids	2.2378	81.7	C42 H80 N O7 P	0.0066	0.1436	0.0168	0.0662
PC (15:1_16:4)	710.4746704	709.46739	M+H	13.483	pos	glycerophospholipids	1.2189	69.9	C39 H68 N O8 P	0.0000	0.0001	0.0001	0.0000
PC (21:0_18:2)	828.6497803	827.6423	M+H	21.572	pos	glycerophospholipids	2.2891	74.7	C47 H90 N O8 P	0.0205	0.0144	0.0331	0.1904
PC (14:0e_24:4)	796.6229858	795.61466	M+H	20.102	pos	glycerophospholipids	0.5898	81.2	C46 H86 N O7 P	0.0449	0.4648	0.0738	0.3976
PC (18:0_18:0)	790.6170654	789.6261	M+H	14.112	pos	glycerophospholipids	1.7028	75.7	C44 H88 N O8 P	0.0001	0.0001	0.0001	0.0006
PC (22:6e_17:0)	806.6022339	805.59495	M+H	17.633	pos	glycerophospholipids	4.4571	79.9	C47 H84 N O7 P	0.0089	0.2006	0.0105	0.1600
PC (16:0_20:5)	780.5563965	779.54762	M+H	15.700	pos	glycerophospholipids	1.4302	85.7	C44 H78 N O8 P	3.8803	1.7031	3.3080	2.9269
PC (22:6e_9:0)	694.4821777	693.4749	M+H	14.663	pos	glycerophospholipids	2.2493	53.4	C39 H68 N O7 P	0.0004	0.0022	0.0005	0.0004
PC (21:0_22:2)	884.7097168	883.70325	M+H	23.301	pos	glycerophospholipids	0.2764	70.3	C51 H98 N O8 P	0.2270	0.0358	0.1545	0.0837
PC (20:3_20:4)	832.5872803	831.57969	M+H	16.247	pos	glycerophospholipids	2.2665	73.6	C48 H82 N O8 P	0.1849	0.3315	0.1636	0.3833
PC (19:2_18:5)	790.5427856	789.52695	M+H	15.557	pos	glycerophospholipids	4.9460	71.4	C45 H76 N O8 P	0.0002	0.0003	0.0000	0.0002
PC (14:0_20:5)	752.5222168	751.51559	M+H	13.556	pos	glycerophospholipids	0.5124	78.8	C42 H74 N O8 P	0.6331	0.0971	0.9128	0.2331
PC (20:1_22:4)	864.6507568	863.64269	M+H	20.107	pos	glycerophospholipids	2.6452	70.6	C50 H90 N O8 P	0.0782	0.0065	0.0677	0.0149
PC (18:2e_4:0)	576.4041748	575.3969	M+H	5.796	pos	glycerophospholipids	3.1460	78	C30 H58 N O7 P	0.0003	0.0001	0.0003	0.0001
PC (20:3e_20:3)	820.6308594	819.61461	M+H	20.256	pos	glycerophospholipids	0.5116	58	C48 H86 N O7 P	0.0006	0.0010	0.0007	0.0013
PC (17:1_20:4)	794.5701294	793.56292	M+H	19.662	pos	glycerophospholipids	0.9638	74.9	C45 H80 N O8 P	0.0216	0.0047	0.0286	0.0090
PC (18:2_22:5)	832.5953979	831.57861	M+H	14.145	pos	glycerophospholipids	0.9678	70.1	C48 H82 N O8 P	0.0089	0.0004	0.0099	0.0035
PC (20:2_22:5)	860.6185303	859.61097	M+H	17.637	pos	glycerophospholipids	2.1692	78.1	C50 H86 N O8 P	0.0151	0.0073	0.0139	0.0264

PC (19:0_20:4)	824.6185303	823.61023	M+H	19.969	pos	glycerophospholipids	1.3655	75.8	C47 H86 N O8 P	0.1613	0.0849	0.1279	0.0495
PC (22:5e_16:0)	794.5988159	793.59994	M+H	19.054	pos	glycerophospholipids	1.7633	82.5	C46 H84 N O7 P	0.0914	0.6320	0.2596	0.8057
PC (16:1_20:4)	780.5549927	779.54994	M+H	18.280	pos	glycerophospholipids	4.4063	69.1	C44 H78 N O8 P	0.0018	0.0020	0.0015	0.0007
PC (14:1_20:4)	752.5250244	751.51728	M+H	16.733	pos	glycerophospholipids	2.7611	72.3	C42 H74 N O8 P	0.0017	0.0030	0.0043	0.0015
PC (16:1_20:5)	778.5407715	777.53315	M+H	17.354	pos	glycerophospholipids	2.9516	77.2	C44 H76 N O8 P	0.0026	0.0019	0.0022	0.0005
PC (18:4e_3:0)	558.3553467	557.34879	M+H	2.692	pos	glycerophospholipids	1.1669	71.1	C29 H52 N O7 P	0.0104	0.0192	0.0214	0.0015
PC (14:1_20:5)	750.5064087	749.49913	M+H	15.376	pos	glycerophospholipids	0.5669	75.1	C42 H72 N O8 P	0.0063	0.0044	0.0105	0.0018
PC (22:6e_14:0)	764.5585327	763.55294	M+H	14.979	pos	glycerophospholipids	1.7674	85.5	C44 H78 N O7 P	0.5791	0.6098	1.1954	0.3754
PC (16:1e_12:0)	662.5139771	661.50506	M+H	15.236	pos	glycerophospholipids	0.6345	76.2	C36 H72 N O7 P	0.0013	0.0016	0.0007	0.0005
PC (17:0_18:5)	766.532959	765.5291	M+H	16.066	pos	glycerophospholipids	2.2926	68.6	C43 H76 N O8 P	0.0163	0.0065	0.0073	0.0171
PC (24:0_18:1)	872.7112427	871.70431	M+H	23.911	pos	glycerophospholipids	1.4962	80.8	C50 H98 N O8 P	0.1762	0.0041	0.2837	0.6137
PC (19:0_18:3)	798.6021729	797.59506	M+H	18.983	pos	glycerophospholipids	2.0120	75.5	C45 H84 N O8 P	0.0398	0.1340	0.0173	0.0308
PC (15:1_15:1)	702.5088501	701.50083	M+H	12.995	pos	glycerophospholipids	1.8177	76.5	C38 H72 N O8 P	0.0107	0.0348	0.0233	0.0219
PC (23:0_18:2)	856.6783447	855.67391	M+H	22.777	pos	glycerophospholipids	2.5762	78	C49 H94 N O8 P	0.0059	0.0051	0.0087	0.0572
PC (17:0_22:6)	820.5870361	819.57803	M+H	17.270	pos	glycerophospholipids	0.2743	77.3	C47 H82 N O8 P	0.6221	0.0261	0.4767	0.0654
PC (16:2_24:4)	834.602417	833.59565	M+H	14.679	pos	glycerophospholipids	2.6328	72.4	C48 H84 N O8 P	0.0007	0.0002	0.0001	0.0002
PC (16:0_17:1)	746.5714722	745.56423	M+H	18.268	pos	glycerophospholipids	2.7829	74	C41 H80 N O8 P	0.0649	0.0235	0.0457	0.0412
PC (24:2_22:6)	914.6652832	913.65844	M+H	20.690	pos	glycerophospholipids	2.6098	66.5	C54 H92 N O8 P	0.0031	0.0020	0.0031	0.0031
PC (16:1_17:1)	744.5534668	743.54839	M+H	15.340	pos	glycerophospholipids	2.5350	83.7	C41 H78 N O8 P	0.0319	0.0369	0.0303	0.0110
PC (18:2_18:5)	776.5089111	775.51592	M+H	12.189	pos	glycerophospholipids	0.9220	70.1	C44 H74 N O8 P	0.0023	0.0144	0.0092	0.0018
PC (13:0_13:0)	650.4647827	649.46865	M+H	12.085	pos	glycerophospholipids	0.6086	77.2	C34 H68 N O8 P	0.0194	0.0041	0.0370	0.0031
PC (22:3e_16:0)	798.6386719	797.6295	M+H	21.055	pos	glycerophospholipids	0.4272	80.1	C46 H88 N O7 P	1.6110	0.5720	1.6841	1.0271
PC (13:0_22:6)	764.5247803	763.51543	M+H	11.375	pos	glycerophospholipids	0.2947	74.9	C43 H74 N O8 P	0.0009	0.0007	0.0007	0.0002
PC (15:1_16:1)	716.5250854	715.51629	M+H	17.395	pos	glycerophospholipids	1.5164	76.8	C39 H74 N O8 P	0.0135	0.0090	0.0109	0.0019
PC (14:1e_20:0)	746.6069946	745.59877	M+H	19.420	pos	glycerophospholipids	0.3076	83.5	C42 H84 N O7 P	0.0113	0.0374	0.0110	0.0043
PC (16:2_18:5)	748.4906616	747.48552	M+H	13.794	pos	glycerophospholipids	2.1608	66	C42 H70 N O8 P	0.0000	0.0002	0.0000	0.0000
PC (18:1_22:6)	832.569397	831.57875	M+H	16.733	pos	glycerophospholipids	1.1361	77.2	C48 H82 N O8 P	0.9676	0.2572	0.8236	1.4202
PC (16:0_17:0)	748.5858154	747.57861	M+H	19.336	pos	glycerophospholipids	1.0765	78.6	C41 H82 N O8 P	0.3531	0.0811	0.2138	0.2430
PC (21:0_22:1)	886.7283325	885.7198	M+H	24.364	pos	glycerophospholipids	1.2918	54.8	C51 H100 N O8 P	0.0076	0.0003	0.0269	0.0278
PC (20:0_20:0)	846.7030029	845.68833	M+H	23.810	pos	glycerophospholipids	1.1521	73.6	C48 H96 N O8 P	0.0254	0.0017	0.0249	0.0368
PC (16:1_18:5)	750.5166626	749.49789	M+H	14.133	pos	glycerophospholipids	2.2213	70.6	C42 H72 N O8 P	0.0008	0.0011	0.0007	0.0006
PC (16:1e_24:4)	822.6533813	821.63068	M+H	19.257	pos	glycerophospholipids	1.0214	82.4	C48 H88 N O7 P	0.0137	0.0051	0.0041	0.0079

PC (19:1_22:6)	846.5870972	845.59516	M+H	18.876	pos	glycerophospholipids	2.0160	83.6	C49 H84 N O8 P	0.0099	0.0003	0.0047	0.0003
PC (16:4_22:6)	798.5213623	797.49749	M+H	13.421	pos	glycerophospholipids	2.5892	70.6	C46 H72 N O8 P	0.0007	0.0001	0.0004	0.0001
PC (20:5e_3:0)	584.3706055	583.36379	M+H	2.155	pos	glycerophospholipids	0.0005	52.3	C31 H54 N O7 P	0.0118	0.0010	0.0059	0.0006
PC (17:2_17:2)	754.5377197	753.53168	M+H	14.880	pos	glycerophospholipids	1.0948	77.5	C42 H76 N O8 P	0.5902	0.1999	0.4659	0.2599
PC (16:0_18:5)	752.524292	751.51451	M+H	14.996	pos	glycerophospholipids	0.9247	71.4	C42 H74 N O8 P	0.0025	0.0094	0.0016	0.0078
PC (16:3_22:4)	804.5444336	803.54601	M+H	12.771	pos	glycerophospholipids	0.6161	59.1	C46 H78 N O8 P	0.0004	0.0002	0.0001	0.0001
PC (14:0e_21:2)	758.6079102	757.60035	M+H	19.402	pos	glycerophospholipids	2.3883	81.7	C43 H84 N O7 P	0.0371	0.1135	0.0379	0.1548
PC (16:2e_19:2)	754.5742188	753.56814	M+H	17.132	pos	glycerophospholipids	1.1936	83.2	C43 H80 N O7 P	0.5238	2.8825	0.6990	2.4914
PC (22:4e_12:0)	740.5585938	739.55352	M+H	16.813	pos	glycerophospholipids	2.6091	81.4	C42 H78 N O7 P	0.0104	0.3595	0.0079	0.0288
PC (14:1_18:2)	728.5238037	727.51783	M+H	17.105	pos	glycerophospholipids	3.6082	75.6	C40 H74 N O8 P	0.0000	0.0002	0.0000	0.0000
PC (17:1_18:3)	768.5562744	767.5488	M+H	18.847	pos	glycerophospholipids	2.9899	66.5	C43 H78 N O8 P	0.0013	0.0008	0.0010	0.0002
PC (18:5e_14:1)	708.4983521	707.49016	M+H	11.474	pos	glycerophospholipids	1.6534	81.3	C40 H70 N O7 P	0.0013	0.0031	0.0094	0.0010
PC (21:1_22:6)	874.6311035	873.62563	M+H	19.961	pos	glycerophospholipids	1.0011	74.3	C51 H88 N O8 P	0.0158	0.0003	0.0366	0.0009
PC (22:2_24:4)	918.6934204	917.68845	M+H	22.513	pos	glycerophospholipids	1.1925	65.6	C54 H96 N O8 P	0.0403	0.0018	0.0669	0.0239
PC (22:5e_13:0)	752.5516357	751.55146	M+H	13.422	pos	glycerophospholipids	0.1736	82.9	C43 H78 N O7 P	0.0001	0.0002	0.0000	0.0002
PC (18:5e_20:1)	792.6005249	791.5805	M+H	15.307	pos	glycerophospholipids	3.0200	83.2	C46 H82 N O7 P	0.0038	0.0009	0.0070	0.0016
PC (18:0_16:1)	760.5872192	759.57819	M+H	16.480	pos	glycerophospholipids	0.5066	72.6	C42 H82 N O8 P	0.0174	0.0012	0.0137	0.0013
PC (22:4e_8:0)	684.4959106	683.48897	M+H	12.454	pos	glycerophospholipids	0.0296	74.1	C38 H70 N O7 P	0.0005	0.0001	0.0042	0.0001
PC (20:5e_22:3)	844.6178589	843.61058	M+H	17.897	pos	glycerophospholipids	4.2801	81.5	C50 H86 N O7 P	0.0021	0.0004	0.0034	0.0005
PC (19:0_22:6)	848.6156006	847.60945	M+H	19.648	pos	glycerophospholipids	0.4066	78.3	C49 H86 N O8 P	0.1910	0.0114	0.1744	0.0779
PC (18:2e_14:0)	716.559021	715.55238	M+H	17.574	pos	glycerophospholipids	1.1034	60.9	C40 H78 N O7 P	0.1707	0.1904	0.2012	0.0556
PC (19:1_19:1)	814.6213379	813.62679	M+H	20.881	pos	glycerophospholipids	2.5007	77.6	C46 H88 N O8 P	0.1080	0.1094	0.1561	0.4766
PC (20:3e_16:3)	764.5609741	763.55126	M+H	15.821	pos	glycerophospholipids	0.4328	81.7	C44 H78 N O7 P	0.2640	0.1381	0.2357	0.0359
PC (18:4e_21:2)	806.6052856	805.5996	M+H	18.799	pos	glycerophospholipids	1.3150	81.9	C47 H84 N O7 P	0.0573	0.0622	0.0645	0.2204
PC (22:3e_19:1)	838.664856	837.65758	M+H	22.198	pos	glycerophospholipids	4.2510	69.6	C49 H92 N O7 P	0.0126	0.0049	0.0264	0.1154
PC (14:0e_20:4)	740.5490112	739.55238	M+H	14.512	pos	glycerophospholipids	1.0676	82.6	C42 H78 N O7 P	0.0167	0.0002	0.0074	0.0003
PC (20:5_22:6)	852.5401001	851.54704	M+H	13.604	pos	glycerophospholipids	0.6282	72.4	C50 H78 N O8 P	1.3547	0.0868	2.2242	0.9695
PC (18:1_20:5)	806.5839233	805.56301	M+H	15.900	pos	glycerophospholipids	1.0612	78.6	C46 H80 N O8 P	2.7208	0.7251	1.7005	1.7374
PC (18:4e_21:1)	806.5708618	807.6162	M+H	20.036	pos	glycerophospholipids	2.4879	82.5	C47 H86 N O7 P	0.0077	0.0536	0.0100	0.0676
PC (16:3_20:4)	776.5216064	775.51563	M+H	11.010	pos	glycerophospholipids	0.5481	67.7	C44 H74 N O8 P	0.0000	0.0000	0.0000	0.0000
PC (14:0e_21:1)	760.6079102	759.61535	M+H	22.901	pos	glycerophospholipids	1.5261	53.3	C43 H86 N O7 P	0.0009	0.0030	0.0037	0.0009
PC (18:4e_17:2)	750.5291138	749.53579	M+H	14.394	pos	glycerophospholipids	0.2007	81.7	C43 H76 N O7 P	0.0361	0.0125	0.1015	0.0075

PC (17:2_18:5)	762.5088501	761.50142	M+H	13.811	pos	glycerophospholipids	2.4493	68.3	C43 H72 N O8 P	0.0001	0.0001	0.0001	0.0000
PC (16:2e_17:2)	726.5452881	725.53792	M+H	15.393	pos	glycerophospholipids	2.7285	81.5	C41 H76 N O7 P	0.0030	0.1119	0.0042	0.0045
PC (14:0e_22:2)	772.612793	771.61613	M+H	20.009	pos	glycerophospholipids	2.5133	84.2	C44 H86 N O7 P	0.0153	0.0387	0.0309	0.1691
PC (16:1_19:1)	772.5870972	771.57982	M+H	15.789	pos	glycerophospholipids	2.6113	73.6	C43 H82 N O8 P	0.0046	0.0072	0.0014	0.0010
PC (15:0_22:5)	794.5686035	793.56312	M+H	16.116	pos	glycerophospholipids	1.2158	72.2	C45 H80 N O8 P	0.2776	0.0601	0.3874	0.0727
PC (14:0_17:0)	720.5531006	719.54967	M+H	18.822	pos	glycerophospholipids	4.3985	71.8	C39 H78 N O8 P	0.0026	0.0007	0.0012	0.0003
PC (22:5e_10:0)	710.513855	709.50564	M+H	12.804	pos	glycerophospholipids	1.4090	82.3	C40 H72 N O7 P	0.0066	0.0058	0.0535	0.0014
PC (18:5e_19:1)	778.5770874	777.56877	M+H	14.281	pos	glycerophospholipids	1.9670	83	C45 H80 N O7 P	0.0001	0.0003	0.0001	0.0018
PC (22:6e_13:0)	750.5314941	749.5352	M+H	18.390	pos	glycerophospholipids	0.9878	65.8	C43 H76 N O7 P	0.0206	0.0083	0.0203	0.0154
PC (18:4_20:4)	802.5375366	801.53026	M+H	14.440	pos	glycerophospholipids	0.7424	62	C46 H76 N O8 P	0.0018	0.0137	0.0008	0.0044
PC (18:5_22:6)	824.5244751	823.51225	M+H	14.327	pos	glycerophospholipids	3.5882	70.7	C48 H74 N O8 P	0.0001	0.0001	0.0000	0.0001
PC (16:2e_8:0)	604.4353027	603.42702	M+H	9.104	pos	glycerophospholipids	1.0441	78.7	C32 H62 N O7 P	0.0005	0.0002	0.0003	0.0012
PC (16:0_16:0)	734.5603638	733.5633	M+H	18.321	pos	glycerophospholipids	1.5607	84.1	C40 H80 N O8 P	1.3465	0.8179	0.8094	1.2069
PC (22:6e_18:1)	818.605957	817.59968	M+H	17.818	pos	glycerophospholipids	1.3935	82.8	C48 H84 N O7 P	0.0273	0.0298	0.0293	0.1788
PC (19:1_18:3)	796.5839233	795.57665	M+H	12.530	pos	glycerophospholipids	1.4520	67.3	C45 H82 N O8 P	0.0001	0.0001	0.0000	0.0000
PC (18:2e_19:0)	786.6435547	785.6317	M+H	21.056	pos	glycerophospholipids	2.3665	81.1	C45 H88 N O7 P	0.0086	0.0126	0.0073	0.0569
PC (22:3_22:4)	888.6438599	887.64177	M+H	20.085	pos	glycerophospholipids	1.5373	70.1	C52 H90 N O8 P	0.0085	0.0015	0.0425	0.0111
PC (14:0e_20:3)	742.5713501	741.5658	M+H	14.706	pos	glycerophospholipids	1.9425	84	C42 H80 N O7 P	0.0027	0.0001	0.0015	0.0000
PC (20:2_20:3)	836.618103	835.61078	M+H	17.796	pos	glycerophospholipids	2.0041	61	C48 H86 N O8 P	0.0016	0.1573	0.0038	0.0072
PC (19:1_19:2)	812.6137695	811.60529	M+H	17.749	pos	glycerophospholipids	4.7010	66.8	C46 H86 N O8 P	0.0043	0.0092	0.0199	0.0539
PC (15:0_20:5)	766.5402832	765.53301	M+H	14.579	pos	glycerophospholipids	2.8150	74.3	C43 H76 N O8 P	0.1813	0.0417	0.1686	0.0664
PC (22:3e_20:3)	848.6549072	847.64446	M+H	19.368	pos	glycerophospholipids	1.2161	83.6	C50 H90 N O7 P	0.0004	0.0001	0.0007	0.0009
PC (21:0_20:4)	852.6500244	851.64264	M+H	21.486	pos	glycerophospholipids	2.6238	69.9	C49 H90 N O8 P	0.0146	0.0039	0.0134	0.0135
PC (18:0e_14:0)	720.5808716	719.5836	M+H	17.316	pos	glycerophospholipids	0.9859	83	C40 H82 N O7 P	0.0122	0.0006	0.0230	0.0002
PC (17:1_18:5)	764.5224609	763.51653	M+H	13.447	pos	glycerophospholipids	1.7354	71.4	C43 H74 N O8 P	0.0092	0.0127	0.0470	0.0240
PC (18:1_19:2)	798.6022949	797.59469	M+H	14.814	pos	glycerophospholipids	1.5481	69.4	C45 H84 N O8 P	0.0001	0.0007	0.0001	0.0001
PC (20:4e_20:2)	820.6239014	819.61442	M+H	20.519	pos	glycerophospholipids	0.2797	75.7	C48 H86 N O7 P	0.0052	0.0099	0.0059	0.0021
PC (17:2_16:4)	736.4909058	735.48575	M+H	13.631	pos	glycerophospholipids	2.5088	71	C41 H70 N O8 P	0.0003	0.0150	0.0003	0.0002
PC (22:5_22:5)	882.6181641	881.59618	M+H	15.664	pos	glycerophospholipids	3.0907	79.8	C52 H84 N O8 P	0.0005	0.0002	0.0048	0.0014
PC (18:2_20:5)	804.5565186	803.54804	M+H	14.351	pos	glycerophospholipids	1.9102	76.5	C46 H78 N O8 P	0.2337	2.0870	0.4334	2.6689
PC (22:6e_17:1)	804.588623	803.58491	M+H	18.142	pos	glycerophospholipids	2.5130	81.7	C47 H82 N O7 P	0.0132	0.0246	0.0207	0.0052
PC (14:0e_22:1)	774.6395874	773.63188	M+H	21.139	pos	glycerophospholipids	2.6359	85.8	C44 H88 N O7 P	0.0238	0.0265	0.0449	0.0322

PC (16:0e_2:0)	524.3707275	523.3638	M+H	4.803	pos	glycerophospholipids	0.0197	82.7	C26 H54 N O7 P	0.4485	0.0245	0.2599	0.0823
PC (17:1_22:6)	818.5686035	817.56342	M+H	19.538	pos	glycerophospholipids	1.5471	77.9	C47 H80 N O8 P	0.0030	0.0002	0.0087	0.0001
PC (22:6e_16:4)	784.5278931	783.52138	M+H	12.177	pos	glycerophospholipids	1.3907	82.1	C46 H74 N O7 P	0.0066	0.0006	0.0141	0.0044
PC (4:0_22:6)	638.3841553	637.37688	M+H	3.521	pos	glycerophospholipids	3.9625	70.3	C34 H56 N O8 P	0.0000	0.0000	0.0000	0.0000
PC (18:5_20:5)	798.5061646	797.49822	M+H	12.418	pos	glycerophospholipids	1.6738	71	C46 H72 N O8 P	0.0009	0.0002	0.0044	0.0003
PC (16:2_16:3)	724.4873047	723.48455	M+H	11.448	pos	glycerophospholipids	0.8918	70.9	C40 H70 N O8 P	0.0132	0.0163	0.1020	0.0061
PC (22:6e_13:1)	748.5271606	747.51714	M+H	18.636	pos	glycerophospholipids	4.2144	82.9	C43 H74 N O7 P	0.0005	0.0003	0.0013	0.0002
PC (16:2_17:2)	740.5311279	739.51415	M+H	16.008	pos	glycerophospholipids	1.4266	71.4	C41 H74 N O8 P	0.0113	0.0006	0.0095	0.0015
PC (14:0e_20:2)	744.5921021	743.58599	M+H	17.980	pos	glycerophospholipids	4.1682	83.6	C42 H82 N O7 P	0.0395	0.2399	0.0817	0.0396
PC (18:2e_16:1)	742.576355	741.56827	M+H	15.810	pos	glycerophospholipids	1.3882	83.9	C42 H80 N O7 P	0.0083	0.0008	0.0086	0.0004
PC (2:0_16:2)	534.3279419	533.31344	M+H	1.120	pos	glycerophospholipids	3.1611	56.2	C26 H48 N O8 P	0.0002	0.0113	0.0001	0.0002
PC (22:0_24:2)	926.7565308	925.75046	M+H	24.629	pos	glycerophospholipids	0.5445	69.2	C54 H104 N O8 P	0.0458	0.0118	0.0541	0.0286
PC (18:1e_18:0)	774.6394043	773.63091	M+H	22.264	pos	glycerophospholipids	1.3821	68.2	C44 H88 N O7 P	0.0035	0.0016	0.0054	0.0019
PC (18:3_20:5)	802.5244751	801.53038	M+H	13.235	pos	glycerophospholipids	0.5926	77.8	C46 H76 N O8 P	0.0392	0.0534	0.1587	0.0254
PC (22:4_22:5)	884.6184692	883.61119	M+H	16.988	pos	glycerophospholipids	2.3593	78.1	C52 H86 N O8 P	0.0024	0.0003	0.0048	0.0002
PC (16:0_18:3)	756.5531616	755.54849	M+H	15.856	pos	glycerophospholipids	2.6271	74.4	C42 H78 N O8 P	0.2260	2.2981	0.1567	0.7611
PC (17:0_18:1)	774.5903931	773.59215	M+H	18.633	pos	glycerophospholipids	1.6873	80.3	C43 H84 N O8 P	0.0741	0.0058	0.0095	0.0026
PC (12:0_12:0)	622.4460449	621.43802	M+H	9.940	pos	glycerophospholipids	1.7144	75.8	C32 H64 N O8 P	0.0025	0.0019	0.0187	0.0006
PC (14:0_16:1)	704.524353	703.5157	M+H	14.532	pos	glycerophospholipids	0.7037	74.2	C38 H74 N O8 P	1.0054	0.1451	1.1707	0.1396
PC (18:0e_20:1)	802.6680298	801.66221	M+H	22.583	pos	glycerophospholipids	1.3336	77.3	C46 H92 N O7 P	0.0189	0.0054	0.0595	0.0254
PC (16:1_22:5)	806.5710449	805.56212	M+H	13.638	pos	glycerophospholipids	0.0436	71.4	C46 H80 N O8 P	0.0183	0.0039	0.0148	0.0034
PC (18:0_19:0)	804.6468506	803.64083	M+H	20.302	pos	glycerophospholipids	0.5283	71.1	C45 H90 N O8 P	0.0159	0.0038	0.0064	0.0062
PC (20:5_20:5)	826.5351563	825.53155	M+H	12.733	pos	glycerophospholipids	0.8419	83.3	C48 H76 N O8 P	1.0231	0.1184	2.1423	0.6482
PC (16:1_16:2)	728.5210571	727.51335	M+H	16.016	pos	glycerophospholipids	2.5497	71.2	C40 H74 N O8 P	0.0015	0.0001	0.0005	0.0004
PC (24:1_18:2)	868.6812134	867.67363	M+H	22.054	pos	glycerophospholipids	2.2179	75.2	C50 H94 N O8 P	0.1449	0.0219	0.1745	0.8289
PC (22:0_18:1)	844.6810913	843.67263	M+H	22.937	pos	glycerophospholipids	1.0957	77.6	C48 H94 N O8 P	0.2860	0.0130	0.4659	0.6409
PC (18:0e_18:0)	776.6550903	775.64708	M+H	22.410	pos	glycerophospholipids	2.0488	79	C44 H90 N O7 P	0.0033	0.0025	0.0083	0.0155
PC (16:0e_17:1)	732.5794678	731.58496	M+H	18.795	pos	glycerophospholipids	2.8287	81.2	C41 H82 N O7 P	0.1626	0.3565	0.2707	0.0779
PC (15:0_18:3)	742.5401001	741.53267	M+H	14.786	pos	glycerophospholipids	2.4476	80.4	C41 H76 N O8 P	0.0439	0.4670	0.0250	0.1014
PC (14:0e_20:0)	748.6137085	747.61543	M+H	21.150	pos	glycerophospholipids	1.6576	82.5	C42 H86 N O7 P	0.0583	0.0494	0.0817	0.2449
PC (16:2_20:5)	776.5252686	775.51799	M+H	13.128	pos	glycerophospholipids	3.5912	71.7	C44 H74 N O8 P	0.0006	0.0003	0.0017	0.0002
PC (20:1_22:5)	862.6307983	861.62352	M+H	19.154	pos	glycerophospholipids	1.4338	76.4	C50 H88 N O8 P	0.0655	0.0028	0.0421	0.0040

PC (16:0_24:1)	844.6782837	843.67101	M+H	22.695	pos	glycerophospholipids	0.8245	81.5	C48 H94 N O8 P	0.2101	0.0065	0.1509	0.0910
PC (17:1_18:2)	770.5718384	769.56422	M+H	16.303	pos	glycerophospholipids	2.6831	75.4	C43 H80 N O8 P	0.0353	0.2020	0.0368	0.1046
PC (14:0e_19:0)	734.6053467	733.59927	M+H	20.442	pos	glycerophospholipids	0.9942	82.3	C41 H84 N O7 P	0.0194	0.0209	0.0133	0.0258
PC (4:0_18:5)	584.3342285	583.32691	M+H	1.232	pos	glycerophospholipids	0.8472	62.4	C30 H50 N O8 P	0.0077	0.0037	0.0020	0.0004
PC (18:1e_5:0)	592.4332275	591.42691	M+H	10.599	pos	glycerophospholipids	0.8793	69.6	C31 H62 N O7 P	0.0116	0.0008	0.0075	0.0021
PC (20:3_22:6)	856.5718384	855.57879	M+H	16.153	pos	glycerophospholipids	1.1510	70.8	C50 H82 N O8 P	0.0449	0.0238	0.0732	0.0188
PC (16:0_22:6)	806.5718384	805.56296	M+H	16.544	pos	glycerophospholipids	0.9991	75.9	C46 H80 N O8 P	2.5972	0.6873	1.7841	2.6211
PC (17:0_18:0)	776.6040649	775.60861	M+H	20.626	pos	glycerophospholipids	0.6386	74.3	C43 H86 N O8 P	0.2562	0.0278	0.1626	0.1563
PC (18:3_18:3)	778.5400391	777.53282	M+H	13.179	pos	glycerophospholipids	2.5272	79.2	C44 H76 N O8 P	0.0080	0.8146	0.0109	0.0383
PC (18:1e_16:0)	746.5947266	745.6	M+H	19.733	pos	glycerophospholipids	1.9573	85	C42 H84 N O7 P	0.8161	2.3011	1.2274	1.1668
PC (17:0_16:1)	746.5718384	745.56572	M+H	15.522	pos	glycerophospholipids	4.7814	72	C41 H80 N O8 P	0.2084	0.4508	0.3781	0.1981
PC (14:0e_16:1)	690.5450439	689.53699	M+H	15.751	pos	glycerophospholipids	1.5222	83.2	C38 H76 N O7 P	0.0341	0.0574	0.1146	0.0062
PC (16:1_16:1)	730.5431519	729.53146	M+H	14.791	pos	glycerophospholipids	0.8293	80.3	C40 H76 N O8 P	0.9030	0.1184	0.8606	0.0992
PC (13:0_20:5)	738.5074463	737.50025	M+H	12.510	pos	glycerophospholipids	0.9425	73.5	C41 H72 N O8 P	0.0063	0.0064	0.0503	0.0037
PC (17:2_22:6)	816.5657959	815.54881	M+H	17.484	pos	glycerophospholipids	2.8262	75	C47 H78 N O8 P	0.0003	0.0004	0.0002	0.0003
PC (20:5e_20:5)	812.5731201	811.55242	M+H	13.406	pos	glycerophospholipids	1.0222	80.8	C48 H78 N O7 P	0.0003	0.0001	0.0013	0.0001
PC (18:0_22:6)	834.6000366	833.594	M+H	18.654	pos	glycerophospholipids	0.6535	77.5	C48 H84 N O8 P	1.3371	0.1010	0.9344	0.8038
PC (19:2_22:5)	846.5984497	845.59157	M+H	19.935	pos	glycerophospholipids	2.2295	69	C49 H84 N O8 P	0.0029	0.0016	0.0007	0.0008
PC (15:0_20:4)	768.5529175	767.54677	M+H	15.946	pos	glycerophospholipids	0.3451	71.3	C43 H78 N O8 P	0.3300	0.0958	0.1907	0.0551
PC (14:0_18:3)	728.524231	727.51651	M+H	13.730	pos	glycerophospholipids	1.7938	76.1	C40 H74 N O8 P	0.0499	0.1628	0.0775	0.0799
PC (20:1_20:2)	840.6499634	839.64125	M+H	20.686	pos	glycerophospholipids	1.0058	70.6	C48 H90 N O8 P	0.0982	0.1111	0.0738	0.1430
PC (15:0_22:6)	792.5530396	791.54692	M+H	15.501	pos	glycerophospholipids	0.5242	72.5	C45 H78 N O8 P	0.6839	0.1081	0.4424	0.2102
PC (14:0e_18:2)	716.5587158	715.55223	M+H	15.313	pos	glycerophospholipids	0.8938	84.3	C40 H78 N O7 P	0.0884	0.0021	0.0724	0.0007
PC (14:0_15:0)	692.5219727	691.5158	M+H	15.266	pos	glycerophospholipids	0.8605	75.4	C37 H74 N O8 P	0.1329	0.0485	0.0978	0.0395
PC (4:0_16:4)	558.3204346	557.31296	M+H	1.142	pos	glycerophospholipids	2.1637	55	C28 H48 N O8 P	0.0057	0.0101	0.0014	0.0004
PC (18:4_20:5)	800.5377197	799.51565	M+H	11.941	pos	glycerophospholipids	0.5566	72.5	C46 H74 N O8 P	0.0380	0.0083	0.1733	0.0125
PC (16:0_17:2)	744.5560303	743.54871	M+H	18.249	pos	glycerophospholipids	2.9654	71.6	C41 H78 N O8 P	0.0003	0.0001	0.0001	0.0000
PC (26:0_18:1)	900.7437744	899.73552	M+H	24.779	pos	glycerophospholipids	1.3494	67.7	C52 H102 N O8 P	0.0386	0.0178	0.0956	0.1317
PC (14:0e_14:0)	664.5162964	663.52137	M+H	15.434	pos	glycerophospholipids	1.6272	83.2	C36 H74 N O7 P	0.0114	0.0365	0.0272	0.0103
PC (18:0_18:3)	784.5846558	783.57736	M+H	17.984	pos	glycerophospholipids	0.5682	75.5	C44 H82 N O8 P	0.1275	0.4912	0.0804	0.5823
PC (16:1_18:2)	756.5463867	755.54796	M+H	15.307	pos	glycerophospholipids	1.9256	76.6	C42 H78 N O8 P	0.2088	1.0276	0.4337	1.3688
PC (12:0_16:1)	676.491394	675.48425	M+H	12.420	pos	glycerophospholipids	0.5110	75.9	C36 H70 N O8 P	0.0134	0.0097	0.0695	0.0034

PC (18:2_18:2)	782.5692749	781.5623	M+H	16.965	pos	glycerophospholipids	0.1853	77.3	C44 H80 N O8 P	1.9197	1.3878	1.2240	0.6896
PC (19:0_16:1)	774.6030884	773.59383	M+H	17.552	pos	glycerophospholipids	0.4844	73.1	C43 H84 N O8 P	0.1474	0.0022	0.1376	0.0071
PC (21:0_18:1)	830.6657104	829.65832	M+H	22.398	pos	glycerophospholipids	2.7294	75.7	C47 H92 N O8 P	0.1200	0.0182	0.1531	0.1775
PC (13:0_18:2)	716.5245361	715.51714	M+H	13.978	pos	glycerophospholipids	2.7044	74.1	C39 H74 N O8 P	0.0084	0.0127	0.0123	0.0241
PC (15:0_18:1)	746.5689697	745.56288	M+H	17.570	pos	glycerophospholipids	0.9722	73.9	C41 H80 N O8 P	3.8071	1.9177	2.1492	0.7669
PC (22:6e_2:0)	596.3720703	595.36448	M+H	2.906	pos	glycerophospholipids	1.1595	68	C32 H54 N O7 P	0.0054	0.0031	0.1000	0.0018
PC (18:0_22:4)	838.6311035	837.62486	M+H	20.222	pos	glycerophospholipids	0.1249	78	C48 H88 N O8 P	0.3732	0.0252	0.3222	0.0430
PC (14:0_16:0)	706.5377808	705.53138	M+H	16.329	pos	glycerophospholipids	0.7441	82.5	C38 H76 N O8 P	0.5932	0.2148	0.3800	0.3379
PC (18:0_22:5)	836.6156616	835.61077	M+H	18.985	pos	glycerophospholipids	1.9921	78.1	C48 H86 N O8 P	0.1497	0.0880	0.0983	0.0270
PC (17:2_20:5)	790.5377808	789.5329	M+H	13.372	pos	glycerophospholipids	2.5901	76.1	C45 H76 N O8 P	0.0091	0.0105	0.0352	0.0112
PC (21:0_22:6)	876.6453247	875.64134	M+H	21.255	pos	glycerophospholipids	1.0673	73.6	C51 H90 N O8 P	0.0448	0.0017	0.0450	0.0409
PC (20:0_22:6)	862.6307373	861.62504	M+H	20.512	pos	glycerophospholipids	0.3303	75.8	C50 H88 N O8 P	0.1427	0.0093	0.1625	0.0480
PC (18:3_18:4)	776.5221558	775.51457	M+H	14.883	pos	glycerophospholipids	0.8188	69.8	C44 H74 N O8 P	0.0043	0.0049	0.0013	0.0034
PC (18:2_18:3)	780.553833	779.54837	M+H	14.495	pos	glycerophospholipids	2.3923	74	C44 H78 N O8 P	0.0073	2.8953	0.0160	1.0204
PC (17:0_18:2)	772.5861816	771.5789	M+H	16.027	pos	glycerophospholipids	1.4189	75.1	C43 H82 N O8 P	0.0164	0.0017	0.0290	0.0224
PC (22:2_22:6)	886.6309814	885.62503	M+H	19.045	pos	glycerophospholipids	0.3101	79.7	C52 H88 N O8 P	0.0198	0.0027	0.0364	0.0016
PC (18:1_22:4)	836.6186523	835.60976	M+H	18.439	pos	glycerophospholipids	0.7835	71.6	C48 H86 N O8 P	0.0582	0.0154	0.0773	0.0086
PC (15:0_18:4)	740.5245361	739.5161	M+H	13.798	pos	glycerophospholipids	1.2103	74.9	C41 H74 N O8 P	0.0325	0.0318	0.0438	0.0096
PC (16:1_18:1)	758.5719604	757.56165	M+H	17.447	pos	glycerophospholipids	0.6668	78.3	C42 H80 N O8 P	0.6439	0.1921	0.3155	0.2010
PC (19:2_20:5)	818.5562744	817.5642	M+H	14.109	pos	glycerophospholipids	2.5012	70.1	C47 H80 N O8 P	0.0334	0.0018	0.0032	0.0027
PC (12:0_22:6)	750.5072632	749.5001	M+H	12.349	pos	glycerophospholipids	0.7273	76.9	C42 H72 N O8 P	0.0065	0.0037	0.0430	0.0044
LPC 22:0	580.4361572	579.42682	M+H	11.399	pos	glycerophospholipids	0.7422	85	C30 H62 N O7 P	0.0083	0.0007	0.0049	0.0036
LPC 21:1	564.3919678	563.3959	M+H	8.051	pos	glycerophospholipids	1.4380	76.2	C29 H58 N O7 P	0.0174	0.0016	0.0237	0.0005
LPC 12:0	440.2786865	439.27058	M+H	1.347	pos	glycerophospholipids	1.5723	70.8	C20 H42 N O7 P	0.0016	0.0013	0.0018	0.0006
LPC 22:5	570.3548584	569.34866	M+H	2.435	pos	glycerophospholipids	0.9140	76.4	C30 H52 N O7 P	0.0668	0.0516	0.2176	0.0427
LPC 18:0	524.3707275	523.3641	M+H	5.214	pos	glycerophospholipids	0.5929	75.6	C26 H54 N O7 P	0.4937	0.1013	0.2905	0.1697
LPC 18:2	520.3411865	519.33376	M+H	2.323	pos	glycerophospholipids	2.4463	75.3	C26 H50 N O7 P	0.0899	3.2800	0.1534	1.4757
LPC 22:6	568.3408813	567.33305	M+H	2.117	pos	glycerophospholipids	0.9878	75.1	C30 H50 N O7 P	0.5133	0.2815	1.1425	0.3585
LPC 24:4	600.4036255	599.39635	M+H	5.156	pos	glycerophospholipids	2.1024	69.6	C32 H58 N O7 P	0.0013	0.0010	0.0016	0.0001
LPC 17:0	510.3569336	509.34966	M+H	3.045	pos	glycerophospholipids	2.9849	85.2	C25 H52 N O7 P	0.0146	0.0048	0.0113	0.0025
LPC 23:0	594.4511719	593.44353	M+H	12.732	pos	glycerophospholipids	2.5107	80.4	C31 H64 N O7 P	0.0013	0.0004	0.0006	0.0011
LPC 20:1	550.3882446	549.37917	M+H	5.465	pos	glycerophospholipids	0.4910	77.3	C28 H56 N O7 P	0.1081	0.0504	0.0930	0.0175

LPC 16:2	492.3096619	491.30199	M+H	1.633	pos	glycerophospholipids	1.6295	75.3	C24 H46 N O7 P	0.0034	0.0065	0.0059	0.0022
LPC 20:0	552.4019775	551.39659	M+H	8.358	pos	glycerophospholipids	2.7207	77.6	C28 H58 N O7 P	0.0126	0.0038	0.0118	0.0054
LPC 19:0	538.3868408	537.38002	M+H	7.180	pos	glycerophospholipids	1.0798	74.9	C27 H56 N O7 P	0.0628	0.0197	0.0887	0.0180
LPC 16:0	496.3395081	495.33227	M+H	2.876	pos	glycerophospholipids	0.4433	82.2	C24 H50 N O7 P	0.5215	0.3201	0.7040	0.2795
LPC 18:1	522.3568115	521.3491	M+H	3.315	pos	glycerophospholipids	1.8421	76.6	C26 H52 N O7 P	1.6039	4.6531	2.2019	1.2932
LPC 21:0	566.4177246	565.41174	M+H	10.134	pos	glycerophospholipids	1.7688	78.9	C29 H60 N O7 P	0.0027	0.0010	0.0018	0.0015
LPC 21:2	562.3867188	561.37935	M+H	4.984	pos	glycerophospholipids	0.1599	71.7	C29 H56 N O7 P	0.0066	0.0017	0.0064	0.0006
LPC 19:1	536.3725586	535.3647	M+H	4.285	pos	glycerophospholipids	1.7003	77.4	C27 H54 N O7 P	0.0802	0.1140	0.0985	0.0264
LPC 26:2	632.4669189	631.45898	M+H	12.327	pos	glycerophospholipids	2.0428	73.1	C34 H66 N O7 P	0.0027	0.0009	0.0024	0.0130
LPC 17:1	508.3410034	507.3332	M+H	2.596	pos	glycerophospholipids	1.4003	76.1	C25 H50 N O7 P	0.1056	0.1247	0.1703	0.0246
LPC 15:1	480.3010559	479.30126	M+H	1.685	pos	glycerophospholipids	0.1472	75.4	C23 H46 N O7 P	0.0025	0.0023	0.0030	0.0010
LPC 15:0	482.3238525	481.31736	M+H	2.359	pos	glycerophospholipids	1.0814	75.2	C23 H48 N O7 P	0.1115	0.0514	0.0847	0.0314
LPC 17:2	506.3244019	505.31656	M+H	2.191	pos	glycerophospholipids	0.5531	74.7	C25 H48 N O7 P	0.0115	0.0025	0.0052	0.0013
LPC 14:0	468.3097534	467.30167	M+H	1.908	pos	glycerophospholipids	1.0284	76.4	C22 H46 N O7 P	0.0331	0.0096	0.0495	0.0138
LPC 22:2	576.4037476	575.3956	M+H	6.636	pos	glycerophospholipids	0.8866	76.1	C30 H58 N O7 P	0.0228	0.0027	0.0382	0.0013
LPC 26:1	634.4799805	633.47321	M+H	14.295	pos	glycerophospholipids	0.2054	74.9	C34 H68 N O7 P	0.0201	0.0043	0.0150	0.0112
LPC 24:1	606.4511108	605.44257	M+H	11.331	pos	glycerophospholipids	0.8754	80.7	C32 H64 N O7 P	0.0296	0.0020	0.0175	0.0112
LPC 24:0	608.4667969	607.45947	M+H	14.040	pos	glycerophospholipids	2.9301	81.5	C32 H66 N O7 P	0.0023	0.0005	0.0010	0.0039
LPC 24:2	604.4354248	603.4276	M+H	9.870	pos	glycerophospholipids	2.0053	76.3	C32 H62 N O7 P	0.0042	0.0013	0.0038	0.0218
LPC 18:4	516.3099365	515.30242	M+H	1.110	pos	glycerophospholipids	2.3880	79.1	C26 H46 N O7 P	0.0002	0.0014	0.0001	0.0001
LPC 18:3	518.3255615	517.31799	M+H	1.707	pos	glycerophospholipids	2.2240	84.2	C26 H48 N O7 P	0.0093	0.6477	0.0126	0.0389
LPC 22:1	578.4173584	577.41108	M+H	9.118	pos	glycerophospholipids	0.5890	77.7	C30 H60 N O7 P	0.0372	0.0043	0.0379	0.0119
LPC 16:1	494.3251953	493.31742	M+H	2.065	pos	glycerophospholipids	1.1767	75.8	C24 H48 N O7 P	0.3194	0.3014	0.4288	0.0862
LPC 22:4	572.3683472	571.36445	M+H	3.256	pos	glycerophospholipids	1.1557	75.5	C30 H54 N O7 P	0.0436	0.0285	0.1567	0.0087
LPC 22:3	574.3882446	573.38056	M+H	4.397	pos	glycerophospholipids	1.9537	75.3	C30 H56 N O7 P	0.0031	0.0036	0.0077	0.0005
LPC 20:5	542.3256836	541.31753	M+H	1.686	pos	glycerophospholipids	1.2756	75.5	C28 H48 N O7 P	0.2967	0.4617	0.7256	0.2094
LPC 20:4	544.3410645	543.33326	M+H	2.240	pos	glycerophospholipids	1.4180	73.1	C28 H50 N O7 P	0.2395	1.1043	0.6489	0.1928
LPC 20:3	546.3569946	545.3491	M+H	2.716	pos	glycerophospholipids	1.7610	75.3	C28 H52 N O7 P	0.0205	0.1102	0.0456	0.0453
LPC 20:2	548.3725586	547.36465	M+H	3.706	pos	glycerophospholipids	1.5717	76.2	C28 H54 N O7 P	0.1160	0.2329	0.1839	0.2098
PE (18:2_20:2)	766.5370483	767.54975	M+H	18.095	neg	glycerophospholipids	4.2276	70	C43 H78 N O8 P	0.0002	0.0108	0.0001	0.0088
PE (17:1_20:5)	748.4951782	749.50294	M-H	15.944	neg	glycerophospholipids	4.5165	76.4	C42 H72 N O8 P	0.0203	0.0042	0.0520	0.0080
PE (14:1e_20:5)	692.4688721	693.47613	M-H	14.915	neg	glycerophospholipids	4.0230	83.3	C39 H68 N O7 P	0.0029	0.0080	0.0087	0.0036

PE (18:1_17:2)	726.5112915	727.51857	M-H	17.695	neg	glycerophospholipids	4.6254	66.8	C40 H74 N O8 P	0.0000	0.0000	0.0000	0.0000
PE (20:0_18:2)	770.5733643	771.58117	M-H	19.104	neg	glycerophospholipids	4.3609	53.8	C43 H82 N O8 P	0.0345	0.1507	0.0505	0.4067
PE (16:2e_22:6)	744.5009155	745.50836	M-H	16.041	neg	glycerophospholipids	4.9895	72.7	C43 H72 N O7 P	0.0060	0.0026	0.0299	0.0063
PE (14:1e_20:4)	694.4842529	695.49174	M-H	16.279	neg	glycerophospholipids	3.9538	82.5	C39 H70 N O7 P	0.0014	0.0046	0.0020	0.0008
PE (2:0_18:0)	522.3225098	523.32979	M-H	5.044	neg	glycerophospholipids	4.5589	73.8	C25 H50 N O8 P	0.0048	0.0016	0.0011	0.0004
PE (21:0_18:1)	786.6051636	787.61244	M-H	22.594	neg	glycerophospholipids	4.2339	62.4	C44 H86 N O8 P	0.0005	0.0002	0.0008	0.0005
PE (15:0_18:0)	704.5272217	705.53438	M-H	19.754	neg	glycerophospholipids	4.9962	63.8	C38 H76 N O8 P	0.0004	0.0002	0.0004	0.0003
PE (15:0_20:4)	724.4959106	725.50264	M-H	15.960	neg	glycerophospholipids	4.2524	70.3	C40 H72 N O8 P	0.0080	0.0125	0.0319	0.0251
PE (18:1_19:1)	756.5578003	757.56508	M-H	19.563	neg	glycerophospholipids	3.8609	74.7	C42 H80 N O8 P	0.0006	0.0027	0.0003	0.0003
PE (18:1e_20:4)	750.5471802	751.55473	M-H	22.319	neg	glycerophospholipids	4.1774	85.4	C43 H78 N O7 P	0.0001	0.0031	0.0000	0.0001
PE (22:1_24:1)	882.6965942	883.70387	M-H	24.571	neg	glycerophospholipids	0.9780	74	C51 H98 N O8 P	0.0000	0.0000	0.0000	0.0000
PE (16:0_26:0)	830.6688232	831.67581	M-H	24.783	neg	glycerophospholipids	4.9351	78.3	C47 H94 N O8 P	0.0001	0.0000	0.0002	0.0001
PE (18:0e_20:5)	750.5459595	751.55534	M-H	16.846	neg	glycerophospholipids	4.9891	79.9	C43 H78 N O7 P	0.0692	0.0921	0.1373	0.1502
PE (18:0e_22:5)	778.5797729	779.58638	M-H	20.652	neg	glycerophospholipids	4.4760	77.1	C45 H82 N O7 P	0.0021	0.0010	0.0076	0.0190
PE (18:1_26:2)	852.6523438	853.65997	M-H	23.184	neg	glycerophospholipids	4.5855	84.9	C49 H92 N O8 P	0.0007	0.0000	0.0006	0.0029
PE (16:1_20:1)	742.5425415	743.54982	M-H	18.698	neg	glycerophospholipids	4.4582	53.1	C41 H78 N O8 P	0.0037	0.0267	0.0013	0.0021
PE (15:0_18:3)	698.479248	699.48659	M-H	15.180	neg	glycerophospholipids	3.8388	80.7	C38 H70 N O8 P	0.0001	0.0010	0.0001	0.0001
PE (26:0_18:1)	856.6845703	857.69145	M-H	24.871	neg	glycerophospholipids	4.7737	88.9	C49 H96 N O8 P	0.0003	0.0000	0.0011	0.0012
PE (2:0_16:0)	494.2912292	495.29832	M-H	2.867	neg	glycerophospholipids	4.4740	69.5	C23 H46 N O8 P	0.0010	0.0009	0.0006	0.0004
PE (20:5_20:5)	782.4802246	783.48719	M-H	13.088	neg	glycerophospholipids	4.1930	82.8	C45 H70 N O8 P	0.0119	0.0050	0.0463	0.0123
PE (14:0e_16:1)	646.4743652	647.49147	M-H	16.318	neg	glycerophospholipids	3.8299	57.2	C35 H70 N O7 P	0.0000	0.0002	0.0001	0.0000
PE (18:1e_20:5)	748.5317383	749.53904	M-H	21.658	neg	glycerophospholipids	4.1354	82.1	C43 H76 N O7 P	0.0002	0.0039	0.0001	0.0003
PE (18:0e_14:0)	676.53125	677.53856	M-H	20.014	neg	glycerophospholipids	3.8664	74.5	C37 H76 N O7 P	0.0002	0.0002	0.0001	0.0005
PE (18:4e_20:5)	742.4848022	743.49208	M-H	14.297	neg	glycerophospholipids	4.1558	82.3	C43 H70 N O7 P	0.0000	0.0005	0.0001	0.0003
PE (19:0_18:2)	756.5578613	757.56514	M-H	20.189	neg	glycerophospholipids	3.9401	58	C42 H80 N O8 P	0.0014	0.0096	0.0024	0.0067
PE (20:3_20:5)	786.5106812	787.51833	M-H	15.310	neg	glycerophospholipids	3.9682	61.1	C45 H74 N O8 P	0.0011	0.0077	0.0030	0.0013
PE (18:1_22:5)	790.5428467	791.55	M-H	21.486	neg	glycerophospholipids	4.4153	66.8	C45 H78 N O8 P	0.0001	0.0001	0.0000	0.0000
PE (18:0e_18:3)	726.5478516	727.55457	M-H	19.552	neg	glycerophospholipids	4.0953	81.3	C41 H78 N O7 P	0.0001	0.0008	0.0001	0.0004
PE (16:0e_16:1)	674.5154419	675.52344	M-H	18.528	neg	glycerophospholipids	4.6626	83.4	C37 H74 N O7 P	0.0004	0.0001	0.0012	0.0002
PE (18:1e_17:1)	714.5480957	715.55499	M-H	20.768	neg	glycerophospholipids	4.7509	74.3	C40 H78 N O7 P	0.0236	0.0299	0.0288	0.0119
PE (18:1e_20:0)	758.6113892	759.61753	M-H	23.506	neg	glycerophospholipids	4.3960	83	C43 H86 N O7 P	0.0001	0.0000	0.0005	0.0003
PE (18:0_16:1)	716.526062	717.53334	M-H	15.885	neg	glycerophospholipids	3.4632	54.8	C39 H76 N O8 P	0.0012	0.0026	0.0005	0.0006

PE (14:1e_16:1)	644.4691772	645.47639	M-H	16.008	neg	glycerophospholipids	4.7250	84.3	C35 H68 N O7 P	0.0117	0.0031	0.0489	0.0170
PE (16:0_17:2)	700.4950562	701.50233	M-H	17.326	neg	glycerophospholipids	3.9560	58.4	C38 H72 N O8 P	0.0000	0.0000	0.0000	0.0000
PE (17:0_16:1)	702.5089722	703.51667	M-H	15.470	neg	glycerophospholipids	2.0825	52.7	C38 H74 N O8 P	0.0435	0.0127	0.0403	0.0056
PE (18:0_17:1)	730.5427246	731.54997	M-H	19.949	neg	glycerophospholipids	4.7364	53	C40 H78 N O8 P	0.0044	0.0032	0.0038	0.0023
PE (18:0e_20:2)	756.5933838	757.60066	M-H	19.931	neg	glycerophospholipids	2.7974	58.8	C43 H84 N O7 P	0.0000	0.0000	0.0000	0.0001
PE (22:0_20:5)	820.59021	821.5972	M-H	21.683	neg	glycerophospholipids	4.5579	69.4	C47 H84 N O8 P	0.0004	0.0002	0.0008	0.0005
PE (14:1e_18:2)	670.4841919	671.4916	M-H	16.530	neg	glycerophospholipids	3.8866	87.9	C37 H70 N O7 P	0.0002	0.0026	0.0003	0.0017
PE (20:2_20:5)	788.5259399	789.53328	M-H	14.246	neg	glycerophospholipids	3.0714	65	C45 H76 N O8 P	0.0002	0.0006	0.0002	0.0004
PE (18:0e_20:4)	752.5539551	753.57075	M-H	18.086	neg	glycerophospholipids	4.6571	79.7	C43 H80 N O7 P	0.0291	0.1207	0.0420	0.1304
PE (18:2e_24:1)	810.6425781	811.64932	M-H	23.588	neg	glycerophospholipids	4.7178	59.8	C47 H90 N O7 P	0.0006	0.0001	0.0011	0.0012
PE (18:3_20:5)	758.4797363	759.48691	M-H	13.377	neg	glycerophospholipids	3.9569	81.5	C43 H70 N O8 P	0.0004	0.0229	0.0016	0.0030
PE (23:0_18:1)	814.637085	815.64447	M-H	23.592	neg	glycerophospholipids	4.9832	52.7	C46 H90 N O8 P	0.0006	0.0001	0.0010	0.0008
PE (16:2e_18:2)	696.5001831	697.50753	M-H	16.766	neg	glycerophospholipids	4.1429	82.2	C39 H72 N O7 P	0.0002	0.0133	0.0003	0.0011
PE (24:1_24:1)	910.7310791	911.73848	M-H	25.275	neg	glycerophospholipids	4.5782	78.9	C53 H102 N O8 P	0.0002	0.0000	0.0001	0.0000
PE (18:1e_18:3)	724.5318604	725.53909	M-H	19.419	neg	glycerophospholipids	4.3411	86.2	C41 H76 N O7 P	0.0019	0.0488	0.0012	0.0069
PE (16:2e_20:5)	718.4844971	719.49188	M-H	15.166	neg	glycerophospholipids	4.0164	79.2	C41 H70 N O7 P	0.0033	0.0110	0.0070	0.0021
PE (14:1e_18:3)	668.4686279	669.4759	M-H	15.122	neg	glycerophospholipids	3.8237	88.4	C37 H68 N O7 P	0.0001	0.0025	0.0001	0.0001
PE (18:1e_22:2)	782.6110229	783.61786	M-H	22.719	neg	glycerophospholipids	4.6825	83.7	C45 H86 N O7 P	0.0004	0.0002	0.0029	0.0004
PE (17:0_15:1)	688.4950562	689.50269	M-H	20.179	neg	glycerophospholipids	4.5469	51.5	C37 H72 N O8 P	0.0000	0.0001	0.0000	0.0000
PE (18:1e_18:0)	730.5787964	731.58648	M-H	22.493	neg	glycerophospholipids	4.9064	86.6	C41 H82 N O7 P	0.0006	0.0003	0.0019	0.0020
PE (17:0_18:0)	732.5588379	733.56441	M-H	20.517	neg	glycerophospholipids	3.0738	51.4	C40 H80 N O8 P	0.0000	0.0001	0.0000	0.0000
PE (18:2_26:2)	850.6363525	851.64381	M-H	22.523	neg	glycerophospholipids	3.9976	57.7	C49 H90 N O8 P	0.0004	0.0001	0.0002	0.0016
PE (18:1e_22:5)	776.5630493	777.57062	M-H	20.453	neg	glycerophospholipids	4.3462	83.8	C45 H80 N O7 P	0.0100	0.0135	0.0515	0.0542
PE (18:2_20:5)	760.4951782	761.50248	M-H	14.716	neg	glycerophospholipids	3.8412	64.6	C43 H72 N O8 P	0.0041	0.0700	0.0072	0.0390
PE (20:1_26:1)	882.6973267	883.70698	M-H	23.697	neg	glycerophospholipids	4.4973	53.5	C51 H98 N O8 P	0.0514	0.0012	0.0837	0.0698
PE (18:2e_22:6)	772.5325928	773.53981	M-H	18.109	neg	glycerophospholipids	5.0025	76.3	C45 H76 N O7 P	0.0289	0.0122	0.0217	0.0159
PE (18:1_21:1)	784.572937	785.59648	M-H	19.804	neg	glycerophospholipids	3.8502	66.5	C44 H84 N O8 P	0.0001	0.0010	0.0001	0.0001
PE (26:1_20:5)	874.6375122	875.64446	M-H	22.694	neg	glycerophospholipids	4.6304	67	C51 H90 N O8 P	0.0004	0.0000	0.0006	0.0001
PE (18:2_18:3)	736.4866333	737.50276	M-H	14.886	neg	glycerophospholipids	4.3459	72	C41 H72 N O8 P	0.0001	0.0213	0.0001	0.0009
PE (16:1e_22:6)	746.5167236	747.52366	M-H	18.004	neg	glycerophospholipids	4.5078	75.6	C43 H74 N O7 P	0.0773	0.1472	0.1620	0.0959
PE (20:1_20:5)	790.5424194	791.54885	M-H	15.808	neg	glycerophospholipids	2.9624	52.9	C45 H78 N O8 P	0.0053	0.0045	0.0007	0.0012
PE (15:0_16:1)	674.4794922	675.48689	M-H	15.993	neg	glycerophospholipids	4.4193	83.3	C36 H70 N O8 P	0.0069	0.0005	0.0080	0.0004

PE (18:3e_20:5)	744.5008545	745.50801	M-H	15.797	neg	glycerophospholipids	4.5201	76.1	C43 H72 N O7 P	0.0076	0.0211	0.0078	0.0082
PE (18:0_22:6)	790.5332642	791.5502	M-H	19.144	neg	glycerophospholipids	4.6680	82.1	C45 H78 N O8 P	0.0614	0.0100	0.0462	0.0334
PE (16:1e_16:1)	672.5001221	673.50757	M-H	18.155	neg	glycerophospholipids	4.3500	86.8	C37 H72 N O7 P	0.0186	0.0335	0.0200	0.0051
PE (16:0e_20:5)	722.5158691	723.52342	M-H	17.504	neg	glycerophospholipids	4.3256	83.3	C41 H74 N O7 P	0.0088	0.0130	0.0305	0.0339
PE (18:0_20:5)	764.5369873	765.53403	M-H	18.396	neg	glycerophospholipids	4.1474	72.1	C43 H76 N O8 P	0.0732	0.0922	0.1009	0.0740
PE (18:1_18:3)	738.5111084	739.51845	M-H	16.455	neg	glycerophospholipids	4.3881	71.3	C41 H74 N O8 P	0.0004	0.0253	0.0003	0.0018
PE (23:0_18:2)	812.6210327	813.62831	M-H	22.958	neg	glycerophospholipids	4.3688	78.8	C46 H88 N O8 P	0.0000	0.0001	0.0001	0.0003
PE (18:1e_24:1)	812.6581421	813.66519	M-H	24.296	neg	glycerophospholipids	4.9764	85.8	C47 H92 N O7 P	0.0006	0.0000	0.0029	0.0007
PE (16:0_16:0)	690.5114746	691.51836	M-H	18.837	neg	glycerophospholipids	4.5625	86.5	C37 H74 N O8 P	0.0011	0.0004	0.0007	0.0012
PE (16:0_24:0)	802.6373291	803.64426	M-H	23.942	neg	glycerophospholipids	4.7963	86.2	C45 H90 N O8 P	0.0003	0.0000	0.0004	0.0005
PE (20:0_20:2)	798.6037598	799.6126	M-H	20.566	neg	glycerophospholipids	4.3705	58.9	C45 H86 N O8 P	0.0491	0.0116	0.0575	0.0479
PE (16:1e_18:1)	700.5315552	701.53887	M-H	19.955	neg	glycerophospholipids	4.1760	85.6	C39 H76 N O7 P	0.0302	0.1887	0.0249	0.0444
PE (18:0e_22:4)	780.5953369	781.60229	M-H	21.463	neg	glycerophospholipids	4.7970	86.4	C45 H84 N O7 P	0.0014	0.0003	0.0066	0.0033
PE (16:0_23:0)	788.6207275	789.62826	M-H	23.483	neg	glycerophospholipids	4.4383	57.2	C44 H88 N O8 P	0.0001	0.0000	0.0001	0.0001
PE (24:0_20:5)	848.6218262	849.62872	M-H	22.870	neg	glycerophospholipids	4.6663	78.3	C49 H88 N O8 P	0.0001	0.0000	0.0003	0.0001
PE (16:0e_18:2)	700.5303955	701.53916	M-H	18.895	neg	glycerophospholipids	4.5894	87.3	C39 H76 N O7 P	0.0002	0.0028	0.0007	0.0080
PE (16:1_17:1)	700.4950562	701.50276	M-H	15.800	neg	glycerophospholipids	4.5689	76.1	C38 H72 N O8 P	0.0006	0.0007	0.0002	0.0003
PE (20:0_20:4)	794.5736084	795.58082	M-H	21.072	neg	glycerophospholipids	3.7894	70	C45 H82 N O8 P	0.0010	0.0039	0.0018	0.0010
PE (16:1_18:1)	714.5111084	715.51828	M-H	16.778	neg	glycerophospholipids	4.2977	81.3	C39 H74 N O8 P	0.0032	0.0205	0.0014	0.0012
PE (18:2e_20:5)	746.5161743	747.52388	M-H	17.294	neg	glycerophospholipids	4.8021	82.2	C43 H74 N O7 P	0.0242	0.0216	0.0381	0.0324
PE (18:1_22:2)	796.5904541	797.59728	M-H	20.916	neg	glycerophospholipids	4.7953	61.1	C45 H84 N O8 P	0.0007	0.0011	0.0068	0.0011
PE (18:0_18:2)	742.5420532	743.54982	M-H	19.595	neg	glycerophospholipids	4.4582	77.3	C41 H78 N O8 P	0.0012	0.0207	0.0014	0.0150
PE (18:1e_21:2)	768.5950928	769.60225	M-H	22.182	neg	glycerophospholipids	4.8198	53.1	C44 H84 N O7 P	0.0002	0.0002	0.0011	0.0001
PE (16:1_18:3)	710.4794922	711.48698	M-H	14.367	neg	glycerophospholipids	4.3222	84.2	C39 H70 N O8 P	0.0002	0.0016	0.0002	0.0001
PE (18:1e_24:0)	814.6740112	815.68079	M-H	25.190	neg	glycerophospholipids	4.9027	90.9	C47 H94 N O7 P	0.0000	0.0000	0.0002	0.0001
PE (18:3e_22:6)	770.5165405	771.52367	M-H	16.636	neg	glycerophospholipids	4.3805	74.5	C45 H74 N O7 P	0.0003	0.0008	0.0007	0.0011
PE (18:1e_18:2)	726.5473633	727.55465	M-H	18.003	neg	glycerophospholipids	4.2052	83.1	C41 H78 N O7 P	0.0020	0.0352	0.0034	0.0292
PE (18:1e_16:1)	700.5316772	701.53847	M-H	17.381	neg	glycerophospholipids	3.6058	73.6	C39 H76 N O7 P	0.0005	0.0010	0.0006	0.0011
PE (15:0_18:2)	700.4950562	701.50238	M-H	16.711	neg	glycerophospholipids	4.0272	78.3	C38 H72 N O8 P	0.0007	0.0043	0.0004	0.0004
PE (16:0_19:0)	732.5583496	733.56563	M-H	21.239	neg	glycerophospholipids	4.7370	69.4	C40 H80 N O8 P	0.0002	0.0001	0.0002	0.0001
PE (22:0_18:2)	798.6052856	799.61255	M-H	22.412	neg	glycerophospholipids	4.3079	67	C45 H86 N O8 P	0.0001	0.0001	0.0003	0.0012
PE (18:1e_22:1)	784.6262207	785.63354	M-H	23.440	neg	glycerophospholipids	4.7086	80.6	C45 H88 N O7 P	0.0005	0.0001	0.0024	0.0004

PE (16:0e_16:0)	676.5328979	677.53897	M-H	17.475	neg	glycerophospholipids	4.4715	55.2	C37 H76 N O7 P	0.0043	0.0131	0.0071	0.0089
PE (17:1_18:2)	726.510437	727.51791	M-H	17.177	neg	glycerophospholipids	3.7182	78.7	C40 H74 N O8 P	0.0001	0.0034	0.0001	0.0006
PE (20:2_22:6)	814.5428467	815.54973	M-H	17.574	neg	glycerophospholipids	3.9543	55.6	C47 H78 N O8 P	0.0014	0.0018	0.0027	0.0031
PE (20:4_22:5)	812.5263062	813.53402	M-H	16.592	neg	glycerophospholipids	3.8904	60.5	C47 H76 N O8 P	0.0009	0.0011	0.0011	0.0002
PE (17:1_18:3)	724.494751	725.50203	M-H	15.387	neg	glycerophospholipids	3.4116	61.9	C40 H72 N O8 P	0.0000	0.0003	0.0000	0.0000
PE (18:1_20:3)	766.5408936	767.54841	M-H	15.899	neg	glycerophospholipids	2.4818	52.7	C43 H78 N O8 P	0.0010	0.0024	0.0013	0.0009
PE (20:1_20:4)	792.5528564	793.56547	M-H	19.041	neg	glycerophospholipids	4.1772	51.5	C45 H80 N O8 P	0.0024	0.0227	0.0017	0.0018
PE (16:0_20:0)	746.5744019	747.58139	M-H	21.868	neg	glycerophospholipids	4.7952	68.8	C41 H82 N O8 P	0.0004	0.0001	0.0004	0.0004
PE (18:1e_20:2)	754.5794067	755.58632	M-H	21.627	neg	glycerophospholipids	4.5388	80.7	C43 H82 N O7 P	0.0019	0.0053	0.0077	0.0082
PE (19:0_20:5)	778.5421753	779.54951	M-H	18.953	neg	glycerophospholipids	3.8547	51.7	C44 H78 N O8 P	0.0024	0.0079	0.0025	0.0011
PE (18:1e_26:2)	838.6732178	839.68065	M-H	24.473	neg	glycerophospholipids	4.5958	76.9	C49 H94 N O7 P	0.0001	0.0000	0.0004	0.0003
PE (16:1_22:6)	760.4961548	761.5031	M-H	15.138	neg	glycerophospholipids	4.6554	72.1	C43 H72 N O8 P	0.0050	0.0016	0.0177	0.0016
PE (18:0e_15:0)	690.5473022	691.5547	M-H	20.796	neg	glycerophospholipids	4.4965	70.4	C38 H78 N O7 P	0.0002	0.0002	0.0001	0.0002
PE (24:1_20:5)	846.6060791	847.61321	M-H	21.565	neg	glycerophospholipids	4.8426	72.9	C49 H86 N O8 P	0.0019	0.0001	0.0023	0.0005
PE (20:1_20:1)	798.605957	799.61299	M-H	21.963	neg	glycerophospholipids	4.8582	62.9	C45 H86 N O8 P	0.0077	0.0005	0.0080	0.0027
PE (18:3_18:3)	734.4798584	735.48712	M-H	13.542	neg	glycerophospholipids	4.3715	79.6	C41 H70 N O8 P	0.0001	0.0062	0.0001	0.0001
PE (18:1e_22:0)	786.642334	787.64937	M-H	24.398	neg	glycerophospholipids	4.9250	87	C45 H90 N O7 P	0.0000	0.0000	0.0003	0.0002
PE (17:1_26:1)	840.6530762	841.66016	M-H	23.462	neg	glycerophospholipids	4.8767	59	C48 H92 N O8 P	0.0019	0.0001	0.0018	0.0006
PE (19:0_20:4)	780.5575562	781.56495	M-H	20.323	neg	glycerophospholipids	3.5760	79.9	C44 H80 N O8 P	0.0020	0.0071	0.0024	0.0016
PE (19:1_18:2)	754.5422363	755.54937	M-H	19.168	neg	glycerophospholipids	3.7918	69.3	C42 H78 N O8 P	0.0001	0.0027	0.0001	0.0008
PE (18:1e_21:1)	770.6109009	771.61789	M-H	23.211	neg	glycerophospholipids	4.7942	80.7	C44 H86 N O7 P	0.0001	0.0001	0.0007	0.0000
PE (16:0e_20:4)	724.5316162	725.53932	M-H	18.739	neg	glycerophospholipids	4.6581	77.2	C41 H76 N O7 P	0.0043	0.0081	0.0071	0.0152
PE (17:2_20:5)	746.4795532	747.4866	M-H	13.946	neg	glycerophospholipids	3.6057	75.7	C42 H70 N O8 P	0.0002	0.0006	0.0002	0.0000
PE (20:0_18:1)	772.5890503	773.59704	M-H	20.329	neg	glycerophospholipids	4.6339	72.2	C43 H84 N O8 P	0.3171	0.1668	0.2913	0.3113
PE (19:2_20:5)	774.5114746	775.51867	M-H	15.877	neg	glycerophospholipids	4.4681	62	C44 H74 N O8 P	0.0040	0.0056	0.0187	0.0052
PE (18:2_20:4)	762.5111084	763.51823	M-H	16.001	neg	glycerophospholipids	3.9620	84.6	C43 H74 N O8 P	0.0080	0.0325	0.0180	0.0626
PE (14:0e_20:5)	694.4848022	695.49185	M-H	15.279	neg	glycerophospholipids	4.1119	75.1	C39 H70 N O7 P	0.0004	0.0030	0.0019	0.0006
PE (25:0_18:1)	842.6688232	843.67583	M-H	24.468	neg	glycerophospholipids	4.8886	53	C48 H94 N O8 P	0.0002	0.0000	0.0003	0.0002
PE (16:1e_18:2)	698.5155029	699.5231	M-H	18.649	neg	glycerophospholipids	4.0166	85.3	C39 H74 N O7 P	0.0049	0.0829	0.0050	0.0575
PE (16:0_20:1)	744.5581665	745.56544	M-H	20.545	neg	glycerophospholipids	4.4059	85.5	C41 H80 N O8 P	0.0032	0.0033	0.0038	0.0062
PE (18:0_22:4)	794.574646	795.58142	M-H	20.559	neg	glycerophospholipids	4.5436	78.2	C45 H82 N O8 P	0.0047	0.0006	0.0082	0.0011
PE (17:0_22:4)	780.5567017	781.56509	M-H	19.377	neg	glycerophospholipids	3.7551	58.9	C44 H80 N O8 P	0.0004	0.0014	0.0006	0.0002

PE (18:1e_20:3)	752.5639038	753.5708	M-H	20.850	neg	glycerophospholipids	4.7235	82.9	C43 H80 N O7 P	0.0009	0.0014	0.0022	0.0026
PE (18:0e_18:2)	728.564209	729.57035	M-H	20.655	neg	glycerophospholipids	4.2621	86.5	C41 H80 N O7 P	0.0022	0.0023	0.0014	0.0274
PE (16:1_20:5)	734.480896	735.4871	M-H	14.231	neg	glycerophospholipids	4.3443	82.5	C41 H70 N O8 P	0.0059	0.0025	0.0206	0.0012
PE (18:0_18:3)	740.5264282	741.53388	M-H	18.538	neg	glycerophospholipids	4.0794	84.1	C41 H76 N O8 P	0.0003	0.0019	0.0003	0.0014
PE (18:1e_17:0)	716.5636597	717.57068	M-H	21.684	neg	glycerophospholipids	4.7932	78.2	C40 H80 N O7 P	0.0066	0.0015	0.0075	0.0043
PE (18:1e_26:1)	840.6896362	841.69664	M-H	25.070	neg	glycerophospholipids	4.9887	86.6	C49 H96 N O7 P	0.0001	0.0000	0.0009	0.0001
PE (16:0_22:0)	774.6057739	775.61276	M-H	22.992	neg	glycerophospholipids	4.7120	86.9	C43 H86 N O8 P	0.0004	0.0000	0.0005	0.0005
PE (17:1_18:1)	728.5264282	729.53391	M-H	18.621	neg	glycerophospholipids	4.1876	68.4	C40 H76 N O8 P	0.0010	0.0075	0.0006	0.0019
PE (18:0_18:1)	744.5598755	745.56548	M-H	18.485	neg	glycerophospholipids	4.4595	61.5	C41 H80 N O8 P	0.4360	0.7106	0.3756	0.5619
PE (18:0_20:1)	772.5877686	773.59505	M-H	20.634	neg	glycerophospholipids	2.0614	58.6	C43 H84 N O8 P	0.0016	0.0008	0.0065	0.0145
PE (17:0_20:4)	752.5273438	753.53414	M-H	18.516	neg	glycerophospholipids	4.3594	81.9	C42 H76 N O8 P	0.0117	0.0096	0.0079	0.0042
PE (18:1_20:2)	768.5592651	769.56523	M-H	17.234	neg	glycerophospholipids	3.9956	56.1	C43 H80 N O8 P	0.0088	0.2780	0.0142	0.2276
PE (20:4_22:6)	810.5118408	811.51858	M-H	15.279	neg	glycerophospholipids	4.1589	79.2	C47 H74 N O8 P	0.0156	0.0042	0.0336	0.0110
PE (17:0_18:1)	730.5427246	731.54941	M-H	17.502	neg	glycerophospholipids	3.9709	61.2	C40 H78 N O8 P	0.1559	0.1284	0.1689	0.0477
PE (20:4_20:4)	786.5111694	787.51823	M-H	15.666	neg	glycerophospholipids	3.8412	68.2	C45 H74 N O8 P	0.0114	0.0082	0.0191	0.0374
PE (18:0e_18:1)	730.5796509	731.586	M-H	19.694	neg	glycerophospholipids	4.2502	83.3	C41 H82 N O7 P	0.0373	0.1964	0.0790	0.0888
PE (14:1e_20:1)	702.5448608	701.53283	M+H	18.945	pos	glycerophospholipids	4.4337	53.3	C39 H76 N O7 P	0.0085	0.0051	0.0050	0.0072
PE (14:0e_22:2)	730.576416	729.56909	M+H	20.726	pos	glycerophospholipids	2.5350	54	C41 H80 N O7 P	0.0019	0.0030	0.0050	0.0464
PE (24:0_17:1)	816.6470947	815.64103	M+H	23.650	pos	glycerophospholipids	0.7657	56.9	C46 H90 N O8 P	0.0017	0.0002	0.0022	0.0015
PE (20:3_22:6)	814.5372314	813.53174	M+H	16.038	pos	glycerophospholipids	1.0878	59.8	C47 H76 N O8 P	0.0092	0.0004	0.0210	0.0013
PE (14:0e_18:0)	678.5426636	677.53524	M+H	20.072	pos	glycerophospholipids	1.0337	52.5	C37 H76 N O7 P	0.0006	0.0002	0.0003	0.0007
PE (18:2_22:6)	788.5237427	787.51636	M+H	15.690	pos	glycerophospholipids	1.4667	76.8	C45 H74 N O8 P	0.0069	0.0244	0.0099	0.0517
PE (18:4_20:4)	760.4933472	759.48552	M+H	13.470	pos	glycerophospholipids	2.1267	50.3	C43 H70 N O8 P	0.0026	0.0496	0.0073	0.0087
PE (19:1_20:4)	780.5565186	779.54867	M+H	19.397	pos	glycerophospholipids	2.7771	59.2	C44 H78 N O8 P	0.0054	0.0068	0.0062	0.0029
PE (14:0_22:6)	736.4906616	735.4857	M+H	14.952	pos	glycerophospholipids	2.4408	66.6	C41 H70 N O8 P	0.0017	0.0003	0.0042	0.0005
PE (14:1e_18:0)	676.5300293	675.52221	M+H	18.614	pos	glycerophospholipids	2.8418	64.1	C37 H74 N O7 P	0.0002	0.0002	0.0009	0.0002
PE (19:1_22:6)	804.5526123	803.54685	M+H	18.245	pos	glycerophospholipids	0.4292	68.7	C46 H78 N O8 P	0.0025	0.0007	0.0054	0.0018
PE (16:2e_18:1)	700.5291748	699.52185	M+H	18.707	pos	glycerophospholipids	2.2296	75.9	C39 H74 N O7 P	0.0060	0.0998	0.0068	0.0740
PE (20:1_20:2)	798.6005859	797.59277	M+H	20.985	pos	glycerophospholipids	0.8592	57.5	C45 H84 N O8 P	0.0062	0.0012	0.0048	0.0065
PE (18:2e_17:0)	716.5582886	715.55101	M+H	21.430	pos	glycerophospholipids	0.8112	63.5	C40 H78 N O7 P	0.0222	0.0050	0.0089	0.0044
PE (15:0_24:1)	788.6159668	787.60869	M+H	22.433	pos	glycerophospholipids	0.5273	51.9	C44 H86 N O8 P	0.0022	0.0003	0.0015	0.0005
PE (17:1_24:1)	814.6317139	813.62483	M+H	22.550	pos	glycerophospholipids	0.0917	63.3	C46 H88 N O8 P	0.0114	0.0005	0.0053	0.0023

PE (22:4_20:5)	814.538208	813.53286	M+H	16.699	pos	glycerophospholipids	2.4645	62	C47 H76 N O8 P	0.0009	0.0011	0.0013	0.0003
PE (18:2e_16:0)	702.5458374	701.53539	M+H	20.624	pos	glycerophospholipids	0.7846	68.3	C39 H76 N O7 P	0.0073	0.0006	0.0039	0.0007
PE (18:3e_18:0)	728.5610962	727.55291	M+H	20.513	pos	glycerophospholipids	1.8137	72.9	C41 H78 N O7 P	0.0208	0.0543	0.0208	0.1472
PE (16:0_17:1)	704.5219727	703.51559	M+H	18.181	pos	glycerophospholipids	0.5473	79.2	C38 H74 N O8 P	0.0192	0.0067	0.0093	0.0020
PE (16:2e_22:5)	748.5296631	747.52112	M+H	18.073	pos	glycerophospholipids	1.1099	77	C43 H74 N O7 P	0.2265	0.1038	0.3581	0.1642
PE (18:3e_18:1)	726.5454712	725.5377	M+H	19.488	pos	glycerophospholipids	2.4252	81.7	C41 H76 N O7 P	0.0036	0.0357	0.0025	0.0129
PE (15:0_20:0)	734.5689697	733.56169	M+H	21.015	pos	glycerophospholipids	0.6341	65.2	C40 H80 N O8 P	0.0037	0.0003	0.0017	0.0013
PE (18:5e_20:0)	752.5610962	751.55286	M+H	18.935	pos	glycerophospholipids	1.6892	70	C43 H78 N O7 P	0.0084	0.0025	0.0254	0.0173
PE (18:4e_20:0)	754.5742798	753.57041	M+H	20.041	pos	glycerophospholipids	4.2060	56.2	C43 H80 N O7 P	0.2210	0.1637	0.3767	0.2925
PE (18:2_19:2)	754.5407715	753.53327	M+H	18.245	pos	glycerophospholipids	3.2049	51.1	C42 H76 N O8 P	0.0133	0.0057	0.0108	0.0022
PE (14:0e_20:0)	706.5767822	705.56833	M+H	21.520	pos	glycerophospholipids	1.5441	53.5	C39 H80 N O7 P	0.0018	0.0002	0.0013	0.0018
PE (14:0e_16:2)	646.4823608	645.47395	M+H	16.080	pos	glycerophospholipids	0.9448	73.4	C35 H68 N O7 P	0.0022	0.0005	0.0026	0.0002
PE (15:0_22:6)	750.5096436	749.50013	M+H	16.051	pos	glycerophospholipids	0.7673	71	C42 H72 N O8 P	0.0176	0.0003	0.0207	0.0022
PE (14:1e_22:6)	720.4958496	719.4896	M+H	15.984	pos	glycerophospholipids	0.8475	52.7	C41 H70 N O7 P	0.0048	0.0018	0.0101	0.0020
PE (18:3e_22:2)	780.5899658	779.58337	M+H	21.324	pos	glycerophospholipids	0.6150	79.8	C45 H82 N O7 P	0.0236	0.0076	0.0820	0.0105
PE (16:2e_22:3)	752.56073	751.55349	M+H	19.637	pos	glycerophospholipids	2.5275	72.9	C43 H78 N O7 P	0.0184	0.0104	0.0276	0.0787
PE (15:0_20:5)	724.4906616	723.48517	M+H	15.165	pos	glycerophospholipids	1.7487	65.2	C40 H70 N O8 P	0.0031	0.0015	0.0081	0.0014
PE (20:2_20:2)	796.5847168	795.57846	M+H	20.618	pos	glycerophospholipids	0.8230	64	C45 H82 N O8 P	0.0124	0.0017	0.0185	0.0009
PE (16:2e_18:0)	702.545105	701.53799	M+H	19.310	pos	glycerophospholipids	2.9216	53.5	C39 H76 N O7 P	0.0009	0.0037	0.0008	0.0003
PE (16:0e_15:1)	662.5116577	661.50481	M+H	18.914	pos	glycerophospholipids	0.2566	56.9	C36 H72 N O7 P	0.0014	0.0004	0.0003	0.0004
PE (14:1e_26:4)	780.5924072	779.58377	M+H	20.718	pos	glycerophospholipids	1.1280	50.9	C45 H82 N O7 P	0.0058	0.0011	0.0193	0.0244
PE (20:3_20:3)	792.5531006	791.54647	M+H	19.202	pos	glycerophospholipids	0.0443	67.8	C45 H78 N O8 P	0.1845	0.0171	0.1370	0.0338
PE (16:0_22:5)	766.5402832	765.53144	M+H	18.432	pos	glycerophospholipids	0.7641	66.4	C43 H76 N O8 P	0.1466	0.1674	0.1280	0.0780
PE (14:0e_21:1)	718.5742798	717.5676	M+H	21.023	pos	glycerophospholipids	0.5010	52.8	C40 H80 N O7 P	0.0057	0.0004	0.0043	0.0022
PE (18:0e_20:3)	756.5900269	755.5841	M+H	21.697	pos	glycerophospholipids	1.6006	63	C43 H82 N O7 P	0.0034	0.0033	0.0096	0.0075
PE (16:2e_22:2)	754.5762939	753.56843	M+H	20.592	pos	glycerophospholipids	1.5785	60.1	C43 H80 N O7 P	0.0053	0.0051	0.0052	0.0159
PE (16:0e_24:4)	782.6057739	781.59964	M+H	21.517	pos	glycerophospholipids	1.4065	50.6	C45 H84 N O7 P	0.0030	0.0008	0.0111	0.0037
PE (14:0e_24:4)	754.5741577	753.56688	M+H	19.938	pos	glycerophospholipids	0.4784	51.7	C43 H80 N O7 P	0.0042	0.0003	0.0004	0.0018
PE (18:1_24:2)	826.6342773	825.62629	M+H	22.319	pos	glycerophospholipids	1.8587	65.7	C47 H88 N O8 P	0.0066	0.0028	0.0075	0.0185
PE (18:1_20:1)	772.5878296	771.58055	M+H	20.738	pos	glycerophospholipids	3.5574	68	C43 H82 N O8 P	0.0061	0.0033	0.0054	0.0024
PE (22:5_22:6)	838.5409546	837.53114	M+H	16.247	pos	glycerophospholipids	0.3403	53.3	C49 H76 N O8 P	0.0060	0.0009	0.0045	0.0026
PE (22:0_18:1)	802.6343384	801.62635	M+H	23.176	pos	glycerophospholipids	1.9892	73.2	C45 H88 N O8 P	0.0073	0.0003	0.0099	0.0113

PE (18:1_26:1)	856.6785889	855.67255	M+H	23.981	pos	glycerophospholipids	0.9868	64.9	C49 H94 N O8 P	0.0154	0.0021	0.0169	0.0065
PE (18:1_20:5)	764.5247192	763.51627	M+H	16.409	pos	glycerophospholipids	1.3949	68.5	C43 H74 N O8 P	0.1197	0.2399	0.0903	0.0764
PE (14:0_20:5)	710.4777222	709.46935	M+H	14.072	pos	glycerophospholipids	1.5437	67.2	C39 H68 N O8 P	0.0020	0.0014	0.0088	0.0015
PE (22:6_22:6)	836.5217896	835.51597	M+H	14.923	pos	glycerophospholipids	0.9157	54.2	C49 H74 N O8 P	0.0920	0.0033	0.1438	0.0747
PE (16:0_18:3)	714.5088501	713.50137	M+H	16.378	pos	glycerophospholipids	2.5439	76.1	C39 H72 N O8 P	0.0035	0.0519	0.0010	0.0068
PE (17:0_22:6)	778.5367432	777.53063	M+H	18.173	pos	glycerophospholipids	0.2894	75.1	C44 H76 N O8 P	0.0567	0.0033	0.0348	0.0148
PE (19:0_18:1)	760.5844116	759.57713	M+H	21.130	pos	glycerophospholipids	0.8889	60.5	C42 H82 N O8 P	0.0142	0.0020	0.0082	0.0042
PE (16:0_22:6)	764.5217896	763.51529	M+H	17.150	pos	glycerophospholipids	0.1114	76.5	C43 H74 N O8 P	0.3311	0.0242	0.2940	0.1000
PE (18:0_22:5)	794.5688477	793.56339	M+H	20.211	pos	glycerophospholipids	1.5561	71.1	C45 H80 N O8 P	0.0150	0.0073	0.0108	0.0050
PE (18:1_18:2)	742.5401611	741.53299	M+H	17.814	pos	glycerophospholipids	2.8791	80.2	C41 H76 N O8 P	0.0019	0.1067	0.0018	0.0160
PE (16:0_20:5)	738.508667	737.50058	M+H	16.258	pos	glycerophospholipids	1.3900	55.3	C41 H72 N O8 P	0.1258	0.0816	0.1548	0.1196
PE (16:1e_20:4)	724.5300903	723.52177	M+H	18.473	pos	glycerophospholipids	2.0451	63.7	C41 H74 N O7 P	0.0531	0.1814	0.0349	0.0326
PE (16:0_18:0)	720.5529785	719.54632	M+H	20.601	pos	glycerophospholipids	0.2572	75	C39 H78 N O8 P	0.0061	0.0003	0.0034	0.0018
PE (18:1_18:1)	744.5560303	743.54836	M+H	19.242	pos	glycerophospholipids	2.4947	76.3	C41 H78 N O8 P	0.0313	0.0599	0.0226	0.0115
PE (14:0_20:4)	712.4909058	711.48532	M+H	15.397	pos	glycerophospholipids	1.9890	60.2	C39 H70 N O8 P	0.0030	0.0019	0.0055	0.0011
PE (17:0_20:5)	752.5220947	751.5162	M+H	17.386	pos	glycerophospholipids	1.3240	64.7	C42 H74 N O8 P	0.0346	0.0124	0.0430	0.0129
PE (16:1_16:1)	688.493103	687.4851	M+H	15.349	pos	glycerophospholipids	1.7385	74.2	C37 H70 N O8 P	0.0041	0.0039	0.0051	0.0006
PE (18:0e_22:6)	778.5744019	777.56823	M+H	20.507	pos	glycerophospholipids	1.2725	67.5	C45 H80 N O7 P	0.0232	0.0088	0.0857	0.0228
PE (18:1_22:1)	800.6168213	799.60966	M+H	22.040	pos	glycerophospholipids	0.6937	70.9	C45 H86 N O8 P	0.0279	0.0013	0.0213	0.0063
PE (16:0_20:4)	740.524353	739.51658	M+H	17.500	pos	glycerophospholipids	1.8594	66.2	C41 H74 N O8 P	0.0620	0.0847	0.0521	0.0473
PE (20:5_22:6)	810.5064087	809.5006	M+H	14.068	pos	glycerophospholipids	1.2911	53.7	C47 H72 N O8 P	0.1387	0.0124	0.3963	0.1429
PE (16:1e_20:5)	722.5140991	721.50568	M+H	17.271	pos	glycerophospholipids	1.4411	68.3	C41 H72 N O7 P	0.2267	0.2087	0.2652	0.1538
PE (16:0_18:1)	718.5376587	717.53169	M+H	19.099	pos	glycerophospholipids	1.1637	73.9	C39 H76 N O8 P	0.0717	0.0763	0.0619	0.0168
PE (18:1e_18:1)	730.5738525	729.5681	M+H	21.481	pos	glycerophospholipids	1.1781	78.3	C41 H80 N O7 P	0.1363	0.0353	0.1247	0.0795
PE (20:4_20:5)	786.5082397	785.4999	M+H	14.469	pos	glycerophospholipids	0.4393	53.1	C45 H72 N O8 P	0.0440	0.0272	0.0815	0.0308
PE (16:1e_18:3)	698.513855	697.50616	M+H	17.412	pos	glycerophospholipids	2.1788	82.9	C39 H72 N O7 P	0.0064	0.1280	0.0021	0.0141
PE (16:0_16:1)	690.5063477	689.50003	M+H	17.220	pos	glycerophospholipids	0.6891	84.5	C37 H72 N O8 P	0.0377	0.0080	0.0289	0.0018
PE (20:5_22:5)	812.5247192	811.51609	M+H	15.365	pos	glycerophospholipids	1.0906	53	C47 H74 N O8 P	0.0651	0.0113	0.1207	0.0372
PE (18:1e_22:6)	776.5611	775.55268	M+H	20.035	pos	glycerophospholipids	1.4049	57.7	C45 H78 N O7 P	0.2208	0.1650	0.3706	0.2985
PE (18:1_24:1)	828.6473389	827.64106	M+H	23.065	pos	glycerophospholipids	0.7908	70.8	C47 H90 N O8 P	0.0712	0.0008	0.0521	0.0306
PE (14:0_16:1)	662.4752808	661.46914	M+H	15.065	pos	glycerophospholipids	1.3383	76.9	C35 H68 N O8 P	0.0034	0.0014	0.0036	0.0004
PE (18:0_20:4)	768.5541382	767.54772	M+H	19.532	pos	glycerophospholipids	1.5829	65.3	C43 H78 N O8 P	0.2074	0.2043	0.1532	0.1198

PE (20:1_22:6)	818.5716553	817.56323	M+H	19.184	pos	glycerophospholipids	1.3147	69.7	C47 H80 N O8 P	0.0076	0.0005	0.0177	0.0005
PE (18:1e_20:1)	758.6078491	757.59931	M+H	22.772	pos	glycerophospholipids	1.0155	72.3	C43 H84 N O7 P	0.0038	0.0010	0.0335	0.0010
PE (18:1_20:4)	766.5377197	765.53244	M+H	17.646	pos	glycerophospholipids	2.0704	68.3	C43 H76 N O8 P	0.0392	0.1627	0.0464	0.0243
PE (16:0_18:2)	716.5245361	715.51707	M+H	17.665	pos	glycerophospholipids	2.6066	75.6	C39 H74 N O8 P	0.0050	0.0668	0.0038	0.0463
PE (16:1e_16:0)	676.5294189	675.5209	M+H	19.880	pos	glycerophospholipids	0.9025	77.8	C37 H74 N O7 P	0.0175	0.0034	0.0118	0.0116
PE (24:0_18:1)	830.6655884	829.65718	M+H	24.108	pos	glycerophospholipids	1.3553	59	C47 H92 N O8 P	0.0050	0.0004	0.0071	0.0099
PE (18:1e_16:0)	704.560791	703.55238	M+H	21.365	pos	glycerophospholipids	1.1222	79.7	C39 H78 N O7 P	0.0504	0.0061	0.0386	0.0534
LPE 20:0	510.3555298	509.34825	M+H	9.550	pos	glycerophospholipids	0.2167	71.6	C25 H52 N O7 P	0.0009	0.0005	0.0012	0.0003
LPE 17:0	468.3181152	467.30101	M+H	3.349	pos	glycerophospholipids	0.3840	69.6	C22 H46 N O7 P	0.0011	0.0004	0.0007	0.0001
LPE 18:0	482.3240662	481.31749	M+H	5.867	pos	glycerophospholipids	1.3515	80.3	C23 H48 N O7 P	0.0757	0.0243	0.0841	0.0355
LPE 22:4	530.3254395	529.31816	M+H	3.477	pos	glycerophospholipids	2.4947	69.3	C27 H48 N O7 P	0.0010	0.0014	0.0030	0.0002
LPE 19:0	496.3395996	495.33236	M+H	6.862	pos	glycerophospholipids	0.2616	73.5	C24 H50 N O7 P	0.0049	0.0005	0.0026	0.0004
LPE 16:1	450.2644043	451.27171	M-H	2.073	neg	glycerophospholipids	4.0346	91	C21 H42 N O7 P	0.0050	0.0033	0.0046	0.0004
LPE 19:1	492.3112488	493.31853	M-H	4.090	neg	glycerophospholipids	3.4268	82	C24 H48 N O7 P	0.0002	0.0011	0.0002	0.0001
LPE 22:5	526.2953491	527.30265	M-H	2.935	neg	glycerophospholipids	2.7699	82.6	C27 H46 N O7 P	0.0025	0.0236	0.0068	0.0028
LPE 20:3	502.2958374	503.303	M-H	2.880	neg	glycerophospholipids	3.5974	59.9	C25 H46 N O7 P	0.0004	0.0037	0.0010	0.0006
LPE 17:1	464.2799377	465.28721	M-H	2.722	neg	glycerophospholipids	3.5905	87.1	C22 H44 N O7 P	0.0017	0.0018	0.0024	0.0004
LPE 24:1	562.3903809	563.39734	M-H	12.099	neg	glycerophospholipids	3.9940	91.7	C29 H58 N O7 P	0.0009	0.0003	0.0006	0.0003
LPE 16:0	452.2802429	453.28727	M-H	3.201	neg	glycerophospholipids	3.8180	89.8	C21 H44 N O7 P	0.0156	0.0147	0.0217	0.0115
LPE 18:2	476.2796631	477.28693	M-H	2.387	neg	glycerophospholipids	2.9136	90.8	C23 H44 N O7 P	0.0010	0.0706	0.0013	0.0076
LPE 18:3	474.2639465	475.27122	M-H	1.790	neg	glycerophospholipids	2.7999	89.2	C23 H42 N O7 P	0.0005	0.0195	0.0003	0.0005
LPE 18:1	478.2952271	479.30277	M-H	3.503	neg	glycerophospholipids	3.2976	89.2	C23 H46 N O7 P	0.0366	0.0704	0.0885	0.0407
LPE 20:2	504.311554	505.31882	M-H	3.921	neg	glycerophospholipids	3.9193	72.4	C25 H48 N O7 P	0.0031	0.0052	0.0028	0.0045
LPE 20:1	506.3268738	507.33406	M-H	5.944	neg	glycerophospholipids	3.0955	90.2	C25 H50 N O7 P	0.0086	0.0139	0.0183	0.0021
LPE 20:5	498.2645264	499.27156	M-H	1.772	neg	glycerophospholipids	3.3463	84.8	C25 H42 N O7 P	0.0334	0.1352	0.0548	0.0234
LPE 20:4	500.2796326	501.28711	M-H	2.321	neg	glycerophospholipids	3.1332	89.6	C25 H44 N O7 P	0.0141	0.1739	0.0232	0.0104
LPE 22:6	524.2800293	525.28704	M-H	2.148	neg	glycerophospholipids	2.8568	80.6	C27 H44 N O7 P	0.0332	0.0327	0.0502	0.0192
PS (20:3_20:3)	808.5164185	835.53837	M-H	17.720	neg	glycerophospholipids	2.4364	58	C46 H78 N O10 P	0.0013	0.0003	0.0021	0.0046
PS (19:2_19:2)	892.6112671	811.53732	M-H	18.121	neg	glycerophospholipids	1.2146	58.9	C44 H78 N O10 P	0.0020	0.0023	0.0033	0.0102
PS (18:0_20:5)	780.4857178	809.52424	M-H	16.862	neg	glycerophospholipids	4.3924	70.9	C44 H76 N O10 P	0.0006	0.0002	0.0033	0.0042
PS (24:1_20:4)	870.6265259	893.61854	M-H	21.173	neg	glycerophospholipids	4.4262	81.5	C50 H88 N O10 P	0.0000	0.0000	0.0001	0.0001
PS (16:0_20:5)	806.5007324	781.49302	M-H	14.657	neg	glycerophospholipids	4.6525	54.6	C42 H72 N O10 P	0.0001	0.0000	0.0003	0.0006

PS (18:1_24:1)	826.4706421	871.63421	M-H	22.013	neg	glycerophospholipids	4.5608	82	C48 H90 N O10 P	0.0004	0.0001	0.0005	0.0008
PS (18:1_20:5)	834.53302	807.50801	M-H	14.829	neg	glycerophospholipids	3.6852	65.1	C44 H74 N O10 P	0.0001	0.0001	0.0006	0.0006
PS (20:5_20:5)	810.5327148	827.47729	M-H	11.730	neg	glycerophospholipids	4.2973	75.5	C46 H70 N O10 P	0.0000	0.0000	0.0002	0.0003
PS (18:0_22:6)	852.4855347	835.54014	M-H	17.652	neg	glycerophospholipids	4.5548	86.8	C46 H78 N O10 P	0.0004	0.0001	0.0020	0.0037
PS (18:0_20:4)	872.6422119	811.53988	M-H	18.025	neg	glycerophospholipids	4.3691	82.2	C44 H78 N O10 P	0.0012	0.0026	0.0030	0.0069
PS (20:5_22:6)	878.5016479	853.49308	M-H	12.571	neg	glycerophospholipids	4.3303	84.1	C48 H72 N O10 P	0.0001	0.0000	0.0006	0.0017
PS (24:0_18:1)	836.5455322	873.64948	M+H	23.142	neg	glycerophospholipids	4.1152	68	C48 H92 N O10 P	0.0000	0.0000	0.0000	0.0002
PS (22:6_22:6)	812.5426025	879.5089	M+H	13.432	neg	glycerophospholipids	4.3954	80.7	C50 H74 N O10 P	0.0001	0.0000	0.0002	0.0008
PI (16:0_20:4)	857.5215454	858.52921	M-H	15.898	neg	glycerophospholipids	3.9379	68.5	C45 H79 O13 P	0.0012	0.0002	0.0112	0.0142
PI (18:0_20:5)	883.5376587	884.54491	M-H	16.753	neg	glycerophospholipids	3.8785	71.3	C47 H81 O13 P	0.0018	0.0032	0.0046	0.0115
PI (20:2_20:5)	907.5377808	908.54491	M-H	15.109	neg	glycerophospholipids	3.7761	61.2	C49 H81 O13 P	0.0001	0.0002	0.0002	0.0003
PI (18:3_20:5)	877.4909668	878.49813	M-H	11.887	neg	glycerophospholipids	4.0990	59.9	C47 H75 O13 P	0.0000	0.0001	0.0001	0.0001
PI (20:4_20:5)	903.5073853	904.51416	M-H	12.889	neg	glycerophospholipids	4.4011	72.1	C49 H77 O13 P	0.0001	0.0001	0.0005	0.0002
PI (16:0_22:6)	881.5222168	882.52949	M-H	15.500	neg	glycerophospholipids	4.1481	58.7	C47 H79 O13 P	0.0000	0.0000	0.0002	0.0003
PI (18:1_20:5)	881.5229492	882.52978	M-H	14.737	neg	glycerophospholipids	4.4767	70.8	C47 H79 O13 P	0.0007	0.0011	0.0020	0.0055
PI (20:5_20:5)	901.4916382	902.49872	M-H	11.663	neg	glycerophospholipids	4.6437	66.4	C49 H75 O13 P	0.0001	0.0000	0.0005	0.0002
PI (18:0_22:6)	909.5531006	910.56068	M-H	17.585	neg	glycerophospholipids	3.8994	58.7	C49 H83 O13 P	0.0000	0.0000	0.0001	0.0002
PI (17:0_20:5)	869.5192261	870.52819	M-H	15.601	neg	glycerophospholipids	2.7119	69.8	C46 H79 O13 P	0.0003	0.0003	0.0003	0.0006
PI (18:0_20:4)	885.5532837	886.56079	M-H	17.933	neg	glycerophospholipids	4.1291	72	C47 H83 O13 P	0.0015	0.0039	0.0031	0.0093
PI (18:1_20:4)	883.5375366	884.54504	M-H	15.973	neg	glycerophospholipids	4.0255	72.6	C47 H81 O13 P	0.0038	0.0022	0.0078	0.0203
PI (17:0_20:4)	871.5354614	872.54372	M-H	16.887	neg	glycerophospholipids	2.5680	70.7	C46 H81 O13 P	0.0002	0.0005	0.0003	0.0004
PI (16:0_20:5)	855.5059814	856.51341	M-H	14.552	neg	glycerophospholipids	3.7721	70.9	C45 H77 O13 P	0.0013	0.0006	0.0021	0.0053
PI (18:2_20:5)	879.5065918	880.51379	M-H	13.184	neg	glycerophospholipids	4.1009	67.6	C47 H77 O13 P	0.0001	0.0005	0.0005	0.0029
PI (20:5_22:6)	927.5074463	928.5142	M-H	12.502	neg	glycerophospholipids	4.3304	64.3	C51 H77 O13 P	0.0000	0.0000	0.0001	0.0001
PG (18:2_18:3)	767.4898682	768.49697	M-H	13.729	neg	glycerophospholipids	3.6888	54.3	C42 H73 O10 P	0.0002	0.0010	0.0001	0.0002
PG (16:1_20:5)	765.4749756	766.48163	M-H	13.106	neg	glycerophospholipids	4.1030	77.6	C42 H71 O10 P	0.0009	0.0001	0.0010	0.0000
PG (16:1_18:1)	745.5061035	746.51148	M-H	15.781	neg	glycerophospholipids	2.2703	78.5	C40 H75 O10 P	0.0044	0.0100	0.0034	0.0027
PG (18:1_22:6)	819.5215454	820.52882	M-H	15.967	neg	glycerophospholipids	4.1250	55.7	C46 H77 O10 P	0.0019	0.0001	0.0008	0.0001
PG (16:0_20:3)	771.5213013	772.52842	M-H	16.762	neg	glycerophospholipids	3.8636	81.8	C42 H77 O10 P	0.0002	0.0077	0.0001	0.0002
PG (16:1_22:6)	791.4907837	792.49754	M-H	13.964	neg	glycerophospholipids	4.2964	77.7	C44 H73 O10 P	0.0019	0.0001	0.0016	0.0000
PG (16:1_16:1)	717.4746704	718.4812	M-H	14.014	neg	glycerophospholipids	3.7787	73	C38 H71 O10 P	0.0012	0.0013	0.0009	0.0001
PG (18:2_18:2)	769.5055542	770.5129	M-H	14.986	neg	glycerophospholipids	4.0425	79.3	C42 H75 O10 P	0.0004	0.0007	0.0001	0.0011

PG (22:6_22:6)	865.5049438	866.51222	M-H	13.738	neg	glycerophospholipids	2.8099	58.6	C50 H75 O10 P	0.0001	0.0000	0.0001	0.0000
PA (18:0_20:4)	723.5001221	724.50777	M-H	18.850	neg	glycerophospholipids	4.7813	89.8	C41 H73 O8 P	0.0004	0.0013	0.0001	0.0004
CL (18:2_18:2_18:2_18:3)	1445.954224	1446.96166	M-H	24.548	neg	glycerophospholipids	3.5134	88	C81 H140 O17 P2	0.0000	0.0015	0.0000	0.0020
CL (18:1_18:3_18:2_22:6)	1495.969116	1496.97714	M-H	24.804	neg	glycerophospholipids	3.2824	81.4	C85 H142 O17 P2	0.0001	0.0000	0.0002	0.0005
CL (18:2_18:3_18:2_18:3)	1443.93811	1444.94578	M-H	24.168	neg	glycerophospholipids	3.3592	83.2	C81 H138 O17 P2	0.0000	0.0017	0.0000	0.0003
CL (18:1_18:2_18:1_18:3)	1449.985596	1450.993	M-H	25.278	neg	glycerophospholipids	3.5311	85.6	C81 H144 O17 P2	0.0001	0.0001	0.0001	0.0010
CL (18:1_18:2_18:1_18:2)	1452.002441	1453.009	M-H	25.603	neg	glycerophospholipids	3.7670	70	C81 H146 O17 P2	0.0002	0.0000	0.0003	0.0001
CL (18:1_20:3_18:2_20:3)	1502.017944	1503.02356	M-H	25.557	neg	glycerophospholipids	2.9164	80.3	C85 H148 O17 P2	0.0000	0.0000	0.0001	0.0005
CL (18:1_18:2_18:1_20:3)	1478.016479	1479.02394	M-H	25.756	neg	glycerophospholipids	3.2207	76.8	C83 H148 O17 P2	0.0001	0.0000	0.0001	0.0007
CL (16:1_18:1_18:2_18:2)	1423.971069	1424.97801	M-H	25.121	neg	glycerophospholipids	4.0588	78.2	C79 H142 O17 P2	0.0001	0.0000	0.0002	0.0001
CL (18:2_18:3_18:3_18:3)	1441.922974	1442.93012	M-H	23.762	neg	glycerophospholipids	3.3570	86.5	C81 H136 O17 P2	0.0000	0.0009	0.0000	0.0000
CL (18:1_20:3_18:1_20:3)	1504.032349	1505.03996	M-H	25.866	neg	glycerophospholipids	3.4108	54.9	C85 H150 O17 P2	0.0000	0.0000	0.0001	0.0010
CL (16:1_18:1_16:1_20:5)	1419.940186	1420.94672	M-H	24.625	neg	glycerophospholipids	4.0774	87.3	C79 H138 O17 P2	0.0001	0.0000	0.0003	0.0000
CL (16:1_18:2_18:2_18:2)	1421.953735	1422.96123	M-H	24.744	neg	glycerophospholipids	3.2705	87.9	C79 H140 O17 P2	0.0000	0.0002	0.0001	0.0004
CL (16:1_20:5_18:1_18:2)	1445.955566	1446.96243	M-H	24.849	neg	glycerophospholipids	4.0455	79.9	C81 H140 O17 P2	0.0003	0.0003	0.0007	0.0001
CL (18:2_18:2_18:2_18:2)	1447.969971	1448.97768	M-H	24.899	neg	glycerophospholipids	3.7638	79	C81 H142 O17 P2	0.0001	0.0006	0.0001	0.0063
CL (18:1_18:2_18:2_22:6)	1497.982666	1498.99072	M-H	25.372	neg	glycerophospholipids	1.8970	75.4	C85 H144 O17 P2	0.0001	0.0000	0.0002	0.0004
CL (16:1_18:1_18:1_18:2)	1425.986694	1426.99371	M-H	25.467	neg	glycerophospholipids	4.0880	86.7	C79 H144 O17 P2	0.0002	0.0000	0.0004	0.0000
CL (18:1_20:3_18:2_18:2)	1476.001465	1477.00878	M-H	25.401	neg	glycerophospholipids	3.5569	80.6	C83 H146 O17 P2	0.0001	0.0000	0.0001	0.0023
CL (18:1_18:3_18:2_20:5)	1469.952515	1470.96007	M-H	24.550	neg	glycerophospholipids	2.3751	82.7	C83 H140 O17 P2	0.0001	0.0001	0.0001	0.0005
TAG (15:0_16:1_18:2)	832.7379761	814.70689	M+NH4	26.329	pos	Glycerolipids	2.2699	68.3	C52 H94 O6	0.0303	0.3132	0.0453	0.1182
TAG (16:0_16:3_18:2)	842.7255249	824.69044	M+NH4	26.093	pos	Glycerolipids	1.2724	58.4	C53 H92 O6	0.1973	0.0959	0.2689	0.0405
TAG (14:0_14:0_20:5)	814.6937866	796.65758	M+NH4	25.283	pos	Glycerolipids	0.6408	56.8	C51 H88 O6	0.0169	0.0490	0.1635	0.0061
TAG (12:0_15:1_18:3)	774.6602783	756.62645	M+NH4	24.311	pos	Glycerolipids	0.4499	52.8	C48 H84 O6	0.0007	0.0144	0.0160	0.0007
TAG (16:0_17:1_18:1)	862.7878418	844.754	M+NH4	28.148	pos	Glycerolipids	2.3783	59.9	C54 H100 O6	0.1258	0.3193	0.0995	0.2155
TAG (16:0_16:0_18:2)	848.7694092	830.73679	M+NH4	24.912	pos	Glycerolipids	0.5407	66.7	C53 H98 O6	0.0112	0.0113	0.0054	0.0074
TAG (15:0_16:0_18:3)	832.7407227	814.70416	M+NH4	26.623	pos	Glycerolipids	1.0810	63.7	C52 H94 O6	0.0559	0.2525	0.0248	0.1098
TAG (18:3_18:3_18:3)	890.7228394	872.68908	M+NH4	24.903	pos	Glycerolipids	0.3560	59.3	C57 H92 O6	0.0532	2.1718	0.0031	0.0127
DAG (17:0_18:1)	626.5734863	608.53976	M+NH4	22.204	pos	Glycerolipids	2.9326	54.1	C38 H72 O5	0.1227	0.0312	0.0737	0.0808
DAG (16:0_21:0)	656.6184692	638.58479	M+NH4	24.257	pos	Glycerolipids	0.2123	64.9	C40 H78 O5	0.0131	0.0032	0.0062	0.0115
DAG (16:2_22:6)	654.5085449	636.47473	M+NH4	17.490	pos	Glycerolipids	1.0136	63.7	C41 H64 O5	0.0042	0.0010	0.0009	0.0002
DAG (16:0_20:0)	642.6032715	624.56983	M+NH4	22.709	pos	Glycerolipids	0.8878	71.2	C39 H76 O5	0.0264	0.1776	0.1122	0.0322

DAG (22:0_20:5)	716.6204834	698.58613	M+NH4	23.557	pos	Glycerolipids	1.7241	61.7	C45 H78 O5	0.0045	0.0009	0.0134	0.0074
DAG (23:0_18:3)	706.6361084	688.60237	M+NH4	24.167	pos	Glycerolipids	2.6058	67.5	C44 H80 O5	0.0008	0.0090	0.0006	0.0011
DAG (16:0_18:0)	614.5713501	596.53781	M+NH4	22.715	pos	Glycerolipids	0.2773	75.3	C37 H72 O5	0.0800	0.0197	0.0418	0.0850
DAG (24:0_18:1)	724.6831665	706.64934	M+NH4	25.663	pos	Glycerolipids	2.5673	68.1	C45 H86 O5	0.0074	0.0019	0.0082	0.0131
DAG (20:3_22:6)	708.5581055	690.5243	M+NH4	18.952	pos	Glycerolipids	2.8597	54.1	C45 H70 O5	0.0115	0.0039	0.0098	0.0172
DAG (15:0_20:4)	620.524231	602.49047	M+NH4	19.273	pos	Glycerolipids	0.9215	54.3	C38 H66 O5	0.0087	0.0108	0.0046	0.0025
SM (d20:1_12:0)	675.5430298	674.53683	M+H	14.258	pos	Sphingolipids	0.8232	83.3	C37 H75 N2 O6 P	2.0153	0.5853	1.3080	0.9522
SM (d19:3_22:0)	797.6551514	796.64723	M+H	19.652	pos	Sphingolipids	1.7634	80.6	C46 H89 N2 O6 P	0.0015	0.0021	0.0017	0.0042
SM (d22:2_16:0)	757.6244507	756.61521	M+H	18.387	pos	Sphingolipids	0.9053	81.9	C43 H85 N2 O6 P	0.1278	0.0215	0.1300	0.0156
SM (d30:2_12:0)	813.6865845	812.67803	M+H	21.531	pos	Sphingolipids	1.1132	78.7	C47 H93 N2 O6 P	0.0633	0.0031	0.0653	0.0794
SM (d25:0_17:0)	817.7180786	816.71015	M+H	23.216	pos	Sphingolipids	2.1116	77.2	C47 H97 N2 O6 P	0.0012	0.0030	0.0034	0.0250
SM (d23:1_12:0)	717.5924683	716.58496	M+H	17.698	pos	Sphingolipids	2.4213	76.7	C40 H81 N2 O6 P	0.0693	0.4477	0.0509	0.0433
SM (d14:0_20:0)	705.5809937	704.5832	M+H	17.275	pos	Sphingolipids	0.0354	83	C39 H81 N2 O6 P	0.0391	0.1234	0.0177	0.0145
SM (d20:1_16:1)	729.5900269	728.58374	M+H	16.480	pos	Sphingolipids	0.7069	81.4	C41 H81 N2 O6 P	0.1483	0.0551	0.0370	0.0129
SM (d26:3_12:0)	755.6083374	754.60105	M+H	16.892	pos	Sphingolipids	2.8823	80.8	C43 H83 N2 O6 P	0.0090	0.0216	0.0033	0.0080
SM (d18:3_24:0)	811.6703491	810.66258	M+H	20.442	pos	Sphingolipids	1.3628	60	C47 H91 N2 O6 P	0.0005	0.0003	0.0004	0.0026
SM (d23:0_12:1)	717.5924683	716.58482	M+H	17.445	pos	Sphingolipids	2.2259	80.1	C40 H81 N2 O6 P	0.1289	0.3411	0.1385	0.2496
SM (d16:0_12:1)	619.4823608	618.47415	M+H	9.294	pos	Sphingolipids	0.7688	78	C33 H67 N2 O6 P	0.0019	0.0002	0.0032	0.0009
SM (d23:3_17:0)	783.6401978	782.63214	M+H	18.770	pos	Sphingolipids	2.5106	79.5	C45 H87 N2 O6 P	0.0055	0.0059	0.0065	0.0169
SM (d25:2_14:0)	771.637085	770.63106	M+H	19.397	pos	Sphingolipids	1.1482	80.9	C44 H87 N2 O6 P	0.1016	0.0123	0.0650	0.0583
SM (d26:0_14:0)	789.6848145	788.67754	M+H	21.968	pos	Sphingolipids	0.5258	80.4	C45 H93 N2 O6 P	0.0062	0.0186	0.0040	0.0044
SM (d15:0_26:0)	803.6995239	802.69196	M+H	22.715	pos	Sphingolipids	1.0158	78.4	C46 H95 N2 O6 P	0.0015	0.0035	0.0034	0.0078
SM (d23:3_16:0)	769.6242065	768.61651	M+H	17.836	pos	Sphingolipids	2.5825	80.2	C44 H85 N2 O6 P	0.0004	0.0022	0.0001	0.0007
SM (d17:0_18:2)	715.5775757	714.56706	M+H	16.017	pos	Sphingolipids	0.7205	81.3	C40 H79 N2 O6 P	0.0078	0.0015	0.0053	0.0030
SM (d30:1_12:0)	815.7022705	814.69365	M+H	21.988	pos	Sphingolipids	1.0735	82.7	C47 H95 N2 O6 P	0.0559	0.0079	0.0251	0.0530
SM (d14:2_19:0)	687.5430908	686.53678	M+H	13.731	pos	Sphingolipids	0.7359	78.1	C38 H75 N2 O6 P	0.0039	0.0006	0.0024	0.0015
SM (d21:1_18:0)	773.6550293	772.64677	M+H	21.166	pos	Sphingolipids	1.2228	85.2	C44 H89 N2 O6 P	0.1164	0.0910	0.1804	0.2444
SM (d25:2_12:0)	743.6057129	742.59934	M+H	17.482	pos	Sphingolipids	0.6262	79	C42 H83 N2 O6 P	0.0645	0.0171	0.0217	0.0170
SM (d26:0_13:0)	775.6680298	774.66251	M+H	21.589	pos	Sphingolipids	1.3357	81.2	C44 H91 N2 O6 P	0.0130	0.0463	0.0194	0.0167
SM (d19:2_22:1)	797.6548462	796.6479	M+H	19.382	pos	Sphingolipids	2.6044	75.7	C46 H89 N2 O6 P	0.0005	0.0024	0.0003	0.0017
SM (d27:1_13:1)	785.6554565	784.64653	M+H	19.426	pos	Sphingolipids	0.8982	83.3	C45 H89 N2 O6 P	0.0090	0.0016	0.0044	0.0428
SM (d27:3_12:1)	767.6065063	766.59926	M+H	16.459	pos	Sphingolipids	0.5022	79.1	C44 H83 N2 O6 P	0.0003	0.0024	0.0001	0.0005

SM (d20:3_16:0)	727.5770874	726.56973	M+H	14.778	pos	Sphingolipids	2.9662	76.8	C41 H79 N2 O6 P	0.0017	0.0041	0.0007	0.0014
SM (d21:0_12:0)	691.5767212	690.56866	M+H	16.143	pos	Sphingolipids	1.5713	81.4	C38 H79 N2 O6 P	0.0296	0.0872	0.0185	0.0210
SM (d22:3_20:0)	811.6704712	810.66357	M+H	20.404	pos	Sphingolipids	2.5840	60	C47 H91 N2 O6 P	0.0038	0.0003	0.0014	0.0009
SM (d18:0_15:1)	689.5588379	688.55156	M+H	14.757	pos	Sphingolipids	0.5298	81	C38 H77 N2 O6 P	0.0121	0.0027	0.0129	0.0041
SM (d14:1_23:0)	745.621521	744.61428	M+H	18.187	pos	Sphingolipids	0.3291	81.7	C42 H85 N2 O6 P	0.0187	0.0014	0.0023	0.0010
SM (d27:1_14:0)	801.684082	800.6772	M+H	21.380	pos	Sphingolipids	0.0933	82.7	C46 H93 N2 O6 P	0.0417	0.0039	0.0232	0.0188
SM (d29:2_12:0)	799.668396	798.66215	M+H	20.909	pos	Sphingolipids	0.8448	81.4	C46 H91 N2 O6 P	0.0800	0.0080	0.0894	0.0541
SM (d24:1_12:0)	731.6082764	730.60088	M+H	18.448	pos	Sphingolipids	2.7443	85.1	C41 H83 N2 O6 P	0.0457	0.1880	0.0398	0.0353
SM (d28:1_12:0)	787.6679077	786.66205	M+H	20.750	pos	Sphingolipids	0.7306	83.5	C45 H91 N2 O6 P	0.2185	0.0055	0.1043	0.1353
SM (d26:1_12:0)	759.6392212	758.63135	M+H	20.414	pos	Sphingolipids	1.5487	84.1	C43 H87 N2 O6 P	0.1536	0.5081	0.2046	0.2365
SM (d24:0_12:0)	733.6240234	732.61584	M+H	19.425	pos	Sphingolipids	1.7948	81.2	C41 H85 N2 O6 P	0.1536	0.1282	0.1134	0.2629
SM (d15:1_14:0)	633.4986572	632.48996	M+H	10.712	pos	Sphingolipids	1.0047	74.7	C34 H69 N2 O6 P	0.0028	0.0005	0.0029	0.0005
SM (d16:1_16:1)	673.529541	672.521	M+H	13.585	pos	Sphingolipids	0.5581	76.7	C37 H73 N2 O6 P	0.0028	0.0009	0.0009	0.0018
SM (d14:3_23:0)	741.59021	740.58293	M+H	16.436	pos	Sphingolipids	0.3983	52.3	C42 H81 N2 O6 P	0.0001	0.0005	0.0000	0.0002
SM (d23:1_14:0)	745.6213379	744.61539	M+H	19.831	pos	Sphingolipids	1.1616	83.2	C42 H85 N2 O6 P	0.2486	0.4708	0.1704	0.2094
SM (d22:2_12:0)	701.5609741	700.5537	M+H	14.911	pos	Sphingolipids	2.5340	81.5	C39 H77 N2 O6 P	0.0171	0.0105	0.0118	0.0118
SM (d20:2_18:0)	757.6238403	756.61575	M+H	17.601	pos	Sphingolipids	1.6190	80.4	C43 H85 N2 O6 P	0.0024	0.0031	0.0007	0.0083
SM (d18:1_23:1)	799.6702881	798.66227	M+H	20.257	pos	Sphingolipids	0.9951	77.2	C46 H91 N2 O6 P	0.0012	0.0003	0.0006	0.0024
SM (d28:0_12:0)	789.6868286	788.67875	M+H	22.236	pos	Sphingolipids	2.0600	80.4	C45 H93 N2 O6 P	0.0092	0.0169	0.0264	0.0501
SM (d26:2_13:0)	771.6391602	770.63196	M+H	18.997	pos	Sphingolipids	2.3161	79.1	C44 H87 N2 O6 P	0.0069	0.0034	0.0065	0.0021
SM (d23:0_13:1)	771.6391602	730.59958	M+H	17.210	pos	Sphingolipids	0.9650	80.7	C41 H83 N2 O6 P	0.0134	0.0032	0.0031	0.0009
SM (d18:1_12:0)	647.5125122	646.50524	M+H	11.875	pos	Sphingolipids	0.4105	82.5	C35 H71 N2 O6 P	0.0870	0.0127	0.1026	0.0316
SM (d18:2_25:0)	827.7022705	826.69499	M+H	22.150	pos	Sphingolipids	2.6789	67.2	C48 H95 N2 O6 P	0.0028	0.0002	0.0031	0.0017
SM (d18:2_22:1)	783.6353149	782.63158	M+H	18.149	pos	Sphingolipids	1.7951	76.2	C45 H87 N2 O6 P	0.0011	0.0004	0.0004	0.0017
SM (d29:0_16:1)	857.7493896	856.74219	M+H	22.121	pos	Sphingolipids	2.8765	73.7	C50 H101 N2 O6 P	0.0061	0.0108	0.0033	0.0030
SM (d22:1_12:0)	703.5768433	702.56946	M+H	16.575	pos	Sphingolipids	2.6832	83.1	C39 H79 N2 O6 P	0.2353	1.1528	0.1894	0.2582
SM (d26:0_16:1)	815.7023926	814.69386	M+H	22.870	pos	Sphingolipids	1.3313	71.4	C47 H95 N2 O6 P	0.0053	0.0015	0.0111	0.0154
SM (d19:0_13:0)	677.5610962	676.55305	M+H	15.021	pos	Sphingolipids	1.6631	82.3	C37 H77 N2 O6 P	0.1362	0.2409	0.0894	0.1292
SM (d29:2_12:1)	797.647644	796.64745	M+H	19.570	pos	Sphingolipids	2.0396	78.1	C46 H89 N2 O6 P	0.0004	0.0001	0.0000	0.0002
SM (d26:0_12:1)	759.6392212	758.6308	M+H	20.687	pos	Sphingolipids	0.8237	80.1	C43 H87 N2 O6 P	0.1848	0.0435	0.1826	0.1408
SM (d17:0_14:1)	661.5297241	660.52115	M+H	13.086	pos	Sphingolipids	0.7953	82.1	C36 H73 N2 O6 P	0.0445	0.0071	0.0502	0.0110
SM (d21:0_12:1)	689.5584717	688.55296	M+H	15.431	pos	Sphingolipids	1.5034	83.5	C38 H77 N2 O6 P	0.4425	0.3889	0.1901	0.3569

SM (d14:3_26:0)	783.6353149	782.62804	M+H	18.145	pos	Sphingolipids	2.7282	76.2	C45 H87 N2 O6 P	0.0012	0.0004	0.0005	0.0016
SM (d14:0_24:0)	761.6550903	760.64695	M+H	20.902	pos	Sphingolipids	1.4787	81.6	C43 H89 N2 O6 P	0.0922	0.1331	0.1096	0.1908
Cer_NS (d17:1_18:0)	552.536499	551.52889	M+H	19.053	pos	Sphingolipids	2.0759	73	C35 H69 N O3	0.0011	0.0040	0.0010	0.0046
Cer_NS (d16:1_18:2)	534.489563	533.48237	M+H	14.969	pos	Sphingolipids	2.9525	76.3	C34 H63 N O3	0.0006	0.0006	0.0008	0.0029
Cer_NS (d17:1_22:1)	606.5838623	605.57589	M+H	20.868	pos	Sphingolipids	1.9729	69.2	C39 H75 N O3	0.0005	0.0001	0.0008	0.0006
Cer_NS (d14:1_22:1)	564.534729	563.52791	M+H	18.649	pos	Sphingolipids	0.2927	85.1	C36 H69 N O3	0.0108	0.0040	0.0039	0.0009
Cer_NS (d16:1_22:1)	592.5679321	591.56066	M+H	20.324	pos	Sphingolipids	2.7297	86.6	C38 H73 N O3	0.0058	0.0014	0.0143	0.0021
Cer_NS (d17:1_20:0)	580.5661621	579.55885	M+H	20.764	pos	Sphingolipids	0.3368	72.5	C37 H73 N O3	0.0032	0.0056	0.0124	0.0055
Cer_NS (d17:1_24:0)	636.6289063	635.62244	M+H	23.561	pos	Sphingolipids	1.2500	81.6	C41 H81 N O3	0.0051	0.0014	0.0144	0.0206
Cer_NS (d14:1_16:1)	480.4415894	479.43402	M+H	12.245	pos	Sphingolipids	0.3657	80.6	C30 H57 N O3	0.0121	0.0009	0.0162	0.0005
Cer_NS (d14:1_14:0)	454.4258118	453.41892	M+H	11.818	pos	Sphingolipids	1.5998	71.1	C28 H55 N O3	0.0035	0.0005	0.0109	0.0007
Cer_NS (d16:1_18:0)	538.5212402	537.51378	M+H	18.421	pos	Sphingolipids	3.1348	88	C34 H67 N O3	0.0131	0.0200	0.0177	0.0398
Cer_NS (d15:1_22:0)	580.5681763	579.56089	M+H	21.319	pos	Sphingolipids	3.1831	79.2	C37 H73 N O3	0.0271	0.0659	0.0353	0.0619
Cer_NS (d14:1_23:1)	578.550354	577.54323	M+H	19.650	pos	Sphingolipids	0.2859	77.1	C37 H71 N O3	0.0114	0.0039	0.0074	0.0041
Cer_NS (d16:1_23:0)	608.5993652	607.59127	M+H	22.544	pos	Sphingolipids	1.5219	83.8	C39 H77 N O3	0.0400	0.0128	0.1107	0.1359
Cer_NS (d14:1_21:0)	552.5368042	551.52863	M+H	19.782	pos	Sphingolipids	1.6045	85.9	C35 H69 N O3	0.0225	0.0615	0.0366	0.0131
Cer_NS (d14:1_15:0)	468.4425964	467.43474	M+H	12.940	pos	Sphingolipids	1.9154	71.5	C29 H57 N O3	0.0005	0.0001	0.0009	0.0001
Cer_NS (d17:1_24:1)	634.6131592	633.6066	M+H	22.345	pos	Sphingolipids	0.9542	75.5	C41 H79 N O3	0.0253	0.0014	0.0355	0.0185
Cer_NS (d16:1_24:0)	622.6151733	621.60702	M+H	23.116	pos	Sphingolipids	1.6483	82.1	C40 H79 N O3	0.0485	0.0056	0.1311	0.1243
Cer_NS (d15:1_24:1)	606.5814209	605.57536	M+H	21.144	pos	Sphingolipids	1.0977	72.1	C39 H75 N O3	0.0439	0.0043	0.0344	0.0189
Cer_NS (d18:1_24:0)	650.6466064	649.6377	M+H	23.994	pos	Sphingolipids	0.6226	82.9	C42 H83 N O3	0.0039	0.0008	0.0114	0.0208
Cer_NS (d14:1_24:0)	594.5817871	593.57536	M+H	22.144	pos	Sphingolipids	1.1199	81.1	C38 H75 N O3	0.0826	0.0106	0.1773	0.0717
Cer_NS (d14:1_18:2)	506.4585266	505.4509	M+H	12.827	pos	Sphingolipids	2.7802	76.6	C32 H59 N O3	0.0018	0.0027	0.0020	0.0045
Cer_NS (d14:1_20:2)	578.4811401	533.48286	M+FA-H	14.882	neg	Sphingolipids	3.8710	83.6	C34 H63 N O3	0.0010	0.0006	0.0013	0.0039
Cer_NS (d14:2_22:0)	608.5279541	563.52975	M+FA-H	19.827	neg	Sphingolipids	3.5578	82.6	C36 H69 N O3	0.0002	0.0078	0.0004	0.0005
Cer_NS (d14:1_19:1)	566.4816284	521.48263	M-H	15.843	neg	Sphingolipids	3.5191	80.3	C33 H63 N O3	0.0012	0.0003	0.0007	0.0008
Cer_NS (d16:1_18:1)	580.4968872	535.49867	M+FA-H	16.461	neg	Sphingolipids	4.1551	79.7	C34 H65 N O3	0.0056	0.0017	0.0068	0.0029
Cer_NS (d16:1_21:0)	578.5540161	579.56112	M-H	21.273	neg	Sphingolipids	3.5800	88.1	C37 H73 N O3	0.0136	0.0783	0.0284	0.0534
Cer_NS (d16:2_16:2)	548.4343262	503.43612	M+FA-H	11.324	neg	Sphingolipids	4.5196	71.6	C32 H57 N O3	0.0006	0.0013	0.0007	0.0004
Cer_NS (d14:1_20:0)	582.5123901	537.51431	M-H	18.638	neg	Sphingolipids	4.1208	88.6	C34 H67 N O3	0.0945	0.1686	0.2288	0.0964
Cer_NS (d14:1_19:0)	522.4909058	523.49825	M-H	17.531	neg	Sphingolipids	3.4481	75.4	C33 H65 N O3	0.0284	0.0597	0.0399	0.0348
Cer_NS (d14:1_22:0)	564.538147	565.5456	M-H	20.613	neg	Sphingolipids	3.8987	87.7	C36 H71 N O3	0.2754	1.0243	0.5166	0.6996

Cer_NS (d16:1_24:2)	616.5686646	617.57731	M-H	20.581	neg	Sphingolipids	4.2339	90.6	C40 H75 N O3	0.0017	0.0066	0.0030	0.0149
Cer_NS (d14:1_23:0)	578.5544434	579.56152	M-H	21.394	neg	Sphingolipids	4.2701	90.1	C37 H73 N O3	0.1268	0.1648	0.2673	0.2872
Cer_NS (d14:1_24:2)	634.5438843	589.54568	M+FA-H	18.986	neg	Sphingolipids	3.8757	67	C38 H71 N O3	0.0017	0.0104	0.0020	0.0025
Cer_NS (d16:1_24:1)	618.5858154	619.5932	M-H	21.713	neg	Sphingolipids	4.6074	89.8	C40 H77 N O3	0.0934	0.0251	0.2388	0.1845
Cer_NS (d18:1_24:1)	692.6224976	647.62462	M+FA-H	22.808	neg	Sphingolipids	4.5930	92.4	C42 H81 N O3	0.0159	0.0039	0.0232	0.0285
Cer_NS (d14:1_24:1)	590.5540771	591.56167	M-H	20.460	neg	Sphingolipids	4.4371	87.6	C38 H73 N O3	0.1088	0.0641	0.0730	0.0716
Cer_NS (d15:1_20:0)	550.5227051	551.53019	M-H	19.717	neg	Sphingolipids	4.4330	90.3	C35 H69 N O3	0.0295	0.0952	0.0607	0.0380
Cer_NS (d14:1_18:0)	508.4748535	509.48255	M-H	16.389	neg	Sphingolipids	3.4449	91.3	C32 H63 N O3	0.1350	0.0626	0.1340	0.1514
Cer_NS (d14:1_16:0)	480.4438477	481.45139	M-H	14.054	neg	Sphingolipids	3.9366	91.1	C30 H59 N O3	0.0156	0.0090	0.0213	0.0103
Cer_NS (d16:1_22:0)	638.5753784	593.57725	M-H	21.857	neg	Sphingolipids	4.3040	87.9	C38 H75 N O3	0.0760	0.1385	0.1874	0.2858
Cer_NDS (d14:0_24:2)	592.5678711	591.56063	M+H	19.616	pos	Sphingolipids	2.6790	64	C38 H73 N O3	0.0010	0.0008	0.0003	0.0019
Cer_NDS (d17:0_22:0)	610.6151123	609.60663	M+H	22.880	pos	Sphingolipids	1.0410	79	C39 H79 N O3	0.0151	0.0144	0.0149	0.0171
Cer_NDS (d14:0_23:1)	580.565979	579.5587	M+H	20.123	pos	Sphingolipids	0.5956	70.9	C37 H73 N O3	0.0029	0.0006	0.0009	0.0006
Cer_NDS (d14:0_23:0)	582.581604	581.5751	M+H	21.828	pos	Sphingolipids	0.6959	73.6	C37 H75 N O3	0.0209	0.0019	0.0102	0.0160
Cer_NDS (d16:0_20:0)	568.5687866	567.56151	M+H	20.806	pos	Sphingolipids	4.3428	84.2	C36 H73 N O3	0.0004	0.0004	0.0002	0.0003
Cer_NDS (d18:0_22:0)	624.6287231	623.62176	M+H	23.412	pos	Sphingolipids	0.1837	76.9	C40 H81 N O3	0.0233	0.0060	0.0295	0.0547
Cer_NDS (d18:0_24:2)	648.6307983	647.62334	M+H	22.380	pos	Sphingolipids	2.6166	72.2	C42 H81 N O3	0.0027	0.0005	0.0027	0.0203
Cer_NDS (d16:0_23:1)	608.59729	607.59091	M+H	21.551	pos	Sphingolipids	0.9294	76.2	C39 H77 N O3	0.0217	0.0012	0.0107	0.0102
Cer_NDS (d14:0_24:1)	594.5818481	593.57452	M+H	19.841	pos	Sphingolipids	0.2953	81.5	C38 H75 N O3	0.0112	0.0006	0.0031	0.0020
Cer_NDS (d18:0_24:0)	652.6621094	651.6542	M+H	24.270	pos	Sphingolipids	1.9250	82.4	C42 H85 N O3	0.0019	0.0008	0.0039	0.0172
Cer_NDS (d16:0_19:0)	554.550293	553.54361	M+H	20.117	pos	Sphingolipids	0.3882	82	C35 H71 N O3	0.0016	0.0012	0.0007	0.0005
Cer_NDS (d20:0_24:1)	678.6776123	677.67034	M+H	24.122	pos	Sphingolipids	2.5741	66.3	C44 H87 N O3	0.0006	0.0002	0.0011	0.0018
Cer_NDS (d18:0_24:1)	650.6465454	649.63841	M+H	23.212	pos	Sphingolipids	1.7155	79.7	C42 H83 N O3	0.0234	0.0010	0.0212	0.0452
Cer_NDS (d15:0_22:0)	582.581543	581.57465	M+H	20.815	pos	Sphingolipids	0.0778	74.4	C37 H75 N O3	0.0036	0.0002	0.0018	0.0017
Cer_NDS (d16:0_23:0)	608.6011963	609.60887	M-H	22.810	neg	Sphingolipids	4.7155	82.8	C39 H79 N O3	0.0152	0.0584	0.0247	0.0325
Cer_NDS (d16:0_24:0)	622.6171265	623.62459	M-H	23.351	neg	Sphingolipids	4.7217	81.2	C40 H81 N O3	0.0156	0.0301	0.0255	0.0520
Cer_NDS (d17:0_24:0)	682.6381836	637.6402	M+FA-H	23.675	neg	Sphingolipids	4.5551	84	C41 H83 N O3	0.0022	0.0151	0.0043	0.0074
Cer_NDS (d14:0_22:0)	566.553894	567.5615	M-H	21.055	neg	Sphingolipids	4.3252	82.2	C36 H73 N O3	0.1064	0.0432	0.1029	0.1765
Cer_NDS (d16:0_22:0)	594.5853271	595.593	M-H	22.231	neg	Sphingolipids	4.4572	81.2	C38 H77 N O3	0.0573	0.0490	0.0843	0.1182
Cer_NDS (d16:0_24:1)	620.6008911	621.60899	M-H	22.118	neg	Sphingolipids	4.8175	81.2	C40 H79 N O3	0.0957	0.0065	0.0822	0.0997
Cer_NDS (d14:0_19:0)	570.512207	525.51426	M-H	18.161	neg	Sphingolipids	4.1198	72.8	C33 H67 N O3	0.0046	0.0091	0.0032	0.0036
Cer_NDS (d14:0_18:0)	510.4909668	511.49853	M-H	17.046	neg	Sphingolipids	4.0764	82.6	C32 H65 N O3	0.0146	0.0124	0.0115	0.0113

Cer_NDS (d17:0_24:1)	634.6173096	635.6245	M-H	22.655	neg	Sphingolipids	4.4910	79.6	C41 H81 N O3	0.0211	0.0165	0.0244	0.0201
Cer_NDS (d14:0_20:0)	538.522583	539.52983	M-H	19.257	neg	Sphingolipids	3.8644	83.2	C34 H69 N O3	0.0150	0.0630	0.0222	0.0368
Cer_ADS (d16:0_23:0)	624.5959473	625.60362	M-H	22.347	neg	Sphingolipids	4.3318	62.2	C39 H79 N O4	0.0004	0.0419	0.0009	0.0015
Cer_ADS (d16:0_21:0)	596.5644531	597.57196	M-H	21.029	neg	Sphingolipids	3.9328	57.9	C37 H75 N O4	0.0001	0.0074	0.0001	0.0004
Cer_NP (t18:1_22:0)	682.6018677	637.60373	M+FA-H	21.598	neg	Sphingolipids	4.4228	89.3	C40 H79 N O4	0.0018	0.0018	0.0012	0.0085
Cer_NP (t18:0_22:0)	638.6114502	639.61908	M-H	22.293	neg	Sphingolipids	3.9397	74.6	C40 H81 N O4	0.0012	0.0247	0.0021	0.0012
Cer_NP (t18:1_24:0)	710.6334839	665.63523	M+FA-H	22.983	neg	Sphingolipids	4.5368	90.8	C42 H83 N O4	0.0001	0.0026	0.0001	0.0009
Cer_NP (t18:0_16:0)	600.5230713	555.52485	M+FA-H	17.289	neg	Sphingolipids	3.9428	86.3	C34 H69 N O4	0.0002	0.0002	0.0004	0.0008
Cer_NP (t18:0_24:1)	710.6334229	665.63521	M+FA-H	22.200	neg	Sphingolipids	4.5068	91.7	C42 H83 N O4	0.0013	0.0008	0.0023	0.0014
Cer_NP (t18:0_20:0)	656.5861206	611.58801	M+FA-H	21.108	neg	Sphingolipids	4.4966	86.1	C38 H77 N O4	0.0004	0.0019	0.0015	0.0014
Cer_NP (t18:1_23:0)	696.6176147	651.61934	M+FA-H	22.414	neg	Sphingolipids	4.2662	67.5	C41 H81 N O4	0.0001	0.0061	0.0001	0.0006
Cer_NP (t14:0_22:0)	628.5549316	583.55667	M+FA-H	19.769	neg	Sphingolipids	4.6443	94.2	C36 H73 N O4	0.0005	0.0013	0.0017	0.0014
Cer_NP (t14:0_20:0)	600.5236816	555.52542	M+FA-H	17.593	neg	Sphingolipids	4.9689	91.5	C34 H69 N O4	0.0003	0.0009	0.0013	0.0005
Cer_NP (t18:0_23:0)	652.6277466	653.63518	M-H	22.863	neg	Sphingolipids	4.5436	88.1	C41 H83 N O4	0.0005	0.0568	0.0011	0.0007
Cer_NP (t14:0_18:0)	572.4915161	527.49338	M-H	15.303	neg	Sphingolipids	3.8303	93	C32 H65 N O4	0.0015	0.0012	0.0012	0.0021
Cer_AS (d16:1_23:0)	622.5804443	623.58795	M-H	21.935	neg	Sphingolipids	4.3139	66.3	C39 H77 N O4	0.0019	0.0084	0.0018	0.0258
Cer_AS (d15:1_24:1)	620.56427	621.57213	M-H	20.434	neg	Sphingolipids	4.0544	62.9	C39 H75 N O4	0.0007	0.0004	0.0002	0.0054
Cer_AS (d14:1_20:0)	598.5075073	553.50872	M+FA-H	17.769	neg	Sphingolipids	3.0901	59.6	C34 H67 N O4	0.0001	0.0001	0.0001	0.0003
Cer_AS (d14:1_18:0)	524.4701538	525.47757	M-H	15.486	neg	Sphingolipids	3.5406	63	C32 H63 N O4	0.0005	0.0008	0.0002	0.0051
Cer_AS (d14:1_22:0)	580.5332031	581.54088	M-H	19.888	neg	Sphingolipids	4.4197	65.8	C36 H71 N O4	0.0046	0.0063	0.0016	0.0085
Cer_AS (d16:1_24:1)	634.5806274	635.58802	M-H	21.129	neg	Sphingolipids	4.3425	64.5	C40 H77 N O4	0.0076	0.0013	0.0029	0.0287
Cer_AS (d16:1_24:0)	636.5963745	637.60366	M-H	22.549	neg	Sphingolipids	4.3130	65.9	C40 H79 N O4	0.0019	0.0020	0.0023	0.0210
Cer_AS (d25:1_13:0)	654.5706177	609.57246	M+FA-H	21.248	neg	Sphingolipids	4.6756	68.9	C38 H75 N O4	0.0023	0.0045	0.0015	0.0123
Cer_AS (d17:1_24:1)	694.6019897	649.60382	M-H	21.518	neg	Sphingolipids	4.4797	82.1	C41 H79 N O4	0.0207	0.0014	0.0096	0.0170
Cer_AS (d17:1_16:0)	584.4915161	539.49321	M-H	15.970	neg	Sphingolipids	3.4300	70.7	C33 H65 N O4	0.0000	0.0005	0.0000	0.0001
Cer_AP (t18:1_16:0)	614.5023193	569.50404	M-H	15.455	neg	Sphingolipids	3.7151	84	C34 H67 N O5	0.0001	0.0014	0.0000	0.0007
Cer_AP (t18:0_24:0)	682.6382446	683.64595	M-H	22.992	neg	Sphingolipids	4.6445	66.9	C42 H85 N O5	0.0010	0.0229	0.0016	0.0041
Cer_AP (t18:0_15:0)	556.4962769	557.50404	M-H	15.005	neg	Sphingolipids	3.7951	65.3	C33 H67 N O5	0.0000	0.0029	0.0000	0.0001
HexCer_NDS (d16:0_24:0)	786.6842651	785.67567	M+H	22.558	pos	Sphingolipids	1.5288	74.7	C46 H91 N O8	0.0264	0.0039	0.0329	0.0541
HexCer_NDS (d14:0_21:0)	716.6044312	715.59715	M+H	19.034	pos	Sphingolipids	1.3016	84.6	C41 H81 N O8	0.0018	0.0017	0.0009	0.0007
HexCer_NDS (d14:0_18:0)	674.5560913	673.54976	M+H	15.769	pos	Sphingolipids	0.7299	86.1	C38 H75 N O8	0.0082	0.0076	0.0050	0.0021
HexCer_NDS (d17:0_24:0)	800.6967163	799.69057	M+H	23.027	pos	Sphingolipids	0.5640	79.5	C47 H93 N O8	0.0037	0.0023	0.0064	0.0058

HexCer_NDS (d14:0_19:0)	688.5720825	687.56497	M+H	16.877	pos	Sphingolipids	0.0750	81	C39 H77 N O8	0.0044	0.0021	0.0014	0.0011
HexCer_NDS (d16:0_23:1)	770.6498413	769.64331	M+H	20.519	pos	Sphingolipids	0.1835	79.2	C45 H87 N O8	0.0234	0.0003	0.0083	0.0033
HexCer_NDS (d16:0_24:1)	730.6184692	783.65952	M+H	21.198	pos	Sphingolipids	0.8948	83.1	C46 H89 N O8	0.1325	0.0018	0.0841	0.1027
HexCer_NDS (d15:0_22:0)	744.6337891	743.62651	M+H	20.450	pos	Sphingolipids	1.3564	71.5	C43 H85 N O8	0.0004	0.0001	0.0001	0.0002
HexCer_NDS (d14:0_22:1)	728.6026611	727.59538	M+H	17.936	pos	Sphingolipids	1.1525	63.8	C42 H81 N O8	0.0061	0.0023	0.0019	0.0011
HexCer_NDS (d17:0_22:0)	772.6654053	771.65897	M+H	21.970	pos	Sphingolipids	0.1959	82.1	C45 H89 N O8	0.0190	0.0052	0.0169	0.0106
HexCer_NDS (d16:0_22:1)	756.6340942	755.62755	M+H	19.850	pos	Sphingolipids	0.0414	83.8	C44 H85 N O8	0.0779	0.0032	0.0259	0.0166
HexCer_NDS (d18:0_24:2)	810.6835327	809.67612	M+H	21.402	pos	Sphingolipids	2.0392	77.7	C48 H91 N O8	0.0028	0.0004	0.0037	0.0165
HexCer_NDS (d14:0_22:0)	730.6184692	729.61255	M+H	19.985	pos	Sphingolipids	0.9339	88	C42 H83 N O8	0.1483	0.0087	0.0764	0.0933
HexCer_NDS (d14:0_23:0)	744.6343994	743.62812	M+H	20.811	pos	Sphingolipids	0.8086	89	C43 H85 N O8	0.0233	0.0018	0.0156	0.0126
HexCer_NDS (d18:0_24:1)	812.6994629	811.69136	M+H	22.343	pos	Sphingolipids	1.5290	85.8	C48 H93 N O8	0.0288	0.0076	0.0332	0.0611
HexCer_NDS (d16:0_22:0)	758.6528931	757.64416	M+H	21.319	pos	Sphingolipids	1.3083	85.7	C44 H87 N O8	0.0750	0.0113	0.0682	0.0863
HexCer_NDS (d18:0_24:0)	814.715332	813.70762	M+H	23.481	pos	Sphingolipids	2.2748	81.8	C48 H95 N O8	0.0027	0.0016	0.0056	0.0174
HexCer_NDS (d14:0_20:0)	702.5872803	701.58137	M+H	17.979	pos	Sphingolipids	1.1424	86.2	C40 H79 N O8	0.0189	0.0087	0.0085	0.0106
HexCer_NDS (d17:0_24:1)	798.6812744	797.67516	M+H	21.796	pos	Sphingolipids	0.8664	82.1	C47 H91 N O8	0.0239	0.0003	0.0198	0.0097
HexCer_NDS (d16:0_24:2)	782.6497803	781.64417	M+H	20.072	pos	Sphingolipids	1.2810	63.8	C46 H87 N O8	0.0051	0.0767	0.0357	0.0215
HexCer_NDS (d14:0_24:0)	756.6376343	757.64491	M-H	21.501	pos	Sphingolipids	2.2983	68.2	C44 H87 N O8	0.0001	0.0000	0.0000	0.0001
HexCer_NDS (d21:0_12:0)	732.5665894	687.56831	M+FA-H	16.802	neg	Sphingolipids	4.9327	73.8	C39 H77 N O8	0.0031	0.0047	0.0014	0.0014
HexCer_NS (d14:1_24:2)	752.6049805	751.5977	M+H	17.647	pos	Sphingolipids	1.9711	51.4	C44 H81 N O8	0.0006	0.0002	0.0004	0.0009
HexCer_NS (d14:1_24:1)	754.6184692	753.61204	M+H	19.292	pos	Sphingolipids	0.2274	81.2	C44 H83 N O8	0.0360	0.0016	0.0241	0.0046
HexCer_NS (d16:1_22:2)	752.6055908	751.59831	M+H	21.533	pos	Sphingolipids	2.7827	68.6	C44 H81 N O8	0.0001	0.0003	0.0001	0.0001
HexCer_NS (d16:1_23:1)	768.6369629	767.62966	M+H	21.211	pos	Sphingolipids	2.7895	71.9	C45 H85 N O8	0.0010	0.0045	0.0006	0.0037
HexCer_NS (d15:1_24:1)	768.6346436	767.62737	M+H	19.965	pos	Sphingolipids	0.1937	70.5	C45 H85 N O8	0.0046	0.0002	0.0035	0.0011
HexCer_NS (d16:1_24:1)	782.6498413	781.64376	M+H	20.757	pos	Sphingolipids	0.7564	77	C46 H87 N O8	0.0877	0.0035	0.0741	0.0372
HexCer_NS (d16:1_18:0)	700.574707	699.56743	M+H	17.025	pos	Sphingolipids	3.5902	73.3	C40 H77 N O8	0.0031	0.0020	0.0025	0.0007
HexCer_NS (d14:1_21:0)	714.5893555	713.58208	M+H	18.427	pos	Sphingolipids	2.1182	85.3	C41 H79 N O8	0.0019	0.0036	0.0014	0.0006
HexCer_NS (d16:1_23:0)	770.6502686	769.64381	M+H	21.594	pos	Sphingolipids	0.8332	84.2	C45 H87 N O8	0.0099	0.0037	0.0144	0.0062
HexCer_NS (d17:1_24:0)	798.6818848	797.67461	M+H	22.704	pos	Sphingolipids	0.1769	79	C47 H91 N O8	0.0015	0.0006	0.0024	0.0014
HexCer_NS (d18:1_24:1)	810.6813354	809.67523	M+H	21.965	pos	Sphingolipids	0.9400	75.2	C48 H91 N O8	0.0308	0.0179	0.0223	0.0207
HexCer_NS (d14:1_22:1)	726.5875854	725.58027	M+H	17.263	pos	Sphingolipids	0.4114	75.9	C42 H79 N O8	0.0051	0.0005	0.0020	0.0004
HexCer_NS (d16:1_22:0)	756.6343384	755.62832	M+H	20.899	pos	Sphingolipids	1.0605	85	C44 H85 N O8	0.0586	0.0063	0.0657	0.0409
HexCer_NS (d16:1_24:0)	784.6657104	783.65986	M+H	22.228	pos	Sphingolipids	1.3286	86.6	C46 H89 N O8	0.0196	0.0037	0.0333	0.0282

HexCer_NS (d14:1_18:0)	672.5426025	671.53457	M+H	15.081	pos	Sphingolipids	1.4172	81.4	C38 H73 N O8	0.0313	0.0109	0.0394	0.0200
HexCer_NS (d14:1_19:0)	686.5562744	685.54956	M+H	16.216	pos	Sphingolipids	0.4254	82.9	C39 H75 N O8	0.0045	0.0049	0.0027	0.0024
HexCer_NS (d14:1_22:0)	728.6031494	727.59715	M+H	19.463	pos	Sphingolipids	1.2802	87.5	C42 H81 N O8	0.0754	0.0155	0.0650	0.0478
HexCer_NS (d17:1_24:1)	796.6549683	795.65907	M+H	21.354	pos	Sphingolipids	0.3157	56.1	C47 H89 N O8	0.0128	0.0011	0.0116	0.0064
HexCer_NS (d18:1_24:0)	810.6850586	811.694	M-H	23.114	neg	Sphingolipids	4.7815	63	C48 H93 N O8	0.0001	0.0001	0.0002	0.0006
HexCer_NS (d14:1_24:0)	754.6230469	755.63074	M-H	21.077	neg	Sphingolipids	4.2631	65	C44 H85 N O8	0.0001	0.0001	0.0003	0.0002
HexCer_NS (d14:1_20:0)	698.5596924	699.5684	M-H	17.275	neg	Sphingolipids	4.9768	55.1	C40 H77 N O8	0.0204	0.0311	0.0208	0.0235
GlcADG (13:1_18:3)	721.4561157	722.46339	M-H	7.947	neg	Sterol Lipids	3.9823	52	C40 H66 O11	0.0001	0.0015	0.0001	0.0002
GlcADG (13:1_18:1)	725.487793	726.49518	M-H	11.265	neg	Sterol Lipids	4.6345	50.3	C40 H70 O11	0.0000	0.0014	0.0000	0.0000
